# Evaluation of a Cluster-Randomized Controlled Trial of a Package of Community-Based Maternal and Newborn Interventions in Mirzapur, Bangladesh

**DOI:** 10.1371/journal.pone.0009696

**Published:** 2010-03-24

**Authors:** Gary L. Darmstadt, Yoonjoung Choi, Shams E. Arifeen, Sanwarul Bari, Syed M. Rahman, Ishtiaq Mannan, Habibur Rahman Seraji, Peter J. Winch, Samir K. Saha, A. S. M. Nawshad Uddin Ahmed, Saifuddin Ahmed, Nazma Begum, Anne C. C. Lee, Robert E. Black, Mathuram Santosham, Derrick Crook, Abdullah H. Baqui, for the Bangladesh Projahnmo-2 (Mirzapur) Study Group

**Affiliations:** 1 Department of International Health, Bloomberg School of Public Health, Johns Hopkins University, Baltimore, Maryland, United States of America; 2 Public Health Sciences Division, International Centre for Diarrhoeal Disease Research, Bangladesh (ICDDR,B), Dhaka, Bangladesh; 3 Department of Microbiology, Bangladesh Institute of Child Health, Dhaka Shishu Hospital, Dhaka, Bangladesh; 4 Department of Pediatrics, Kumudini Women's Medical College, Mirzapur, Tangail, Bangladesh; 5 Department of Population and Family Health Sciences, Bloomberg School of Public Health, Johns Hopkins University, Baltimore, Maryland, United States of America; 6 Department of Microbiology, John Radcliffe Hospital, Oxford University, Oxford, United Kingdom; Institute of Clinical Effectiveness and Health Policy, Argentina

## Abstract

**Background:**

To evaluate a delivery strategy for newborn interventions in rural Bangladesh.

**Methods:**

A cluster-randomized controlled trial was conducted in Mirzapur, Bangladesh. Twelve unions were randomized to intervention or comparison arm. All women of reproductive age were eligible to participate. In the intervention arm, community health workers identified pregnant women; made two antenatal home visits to promote birth and newborn care preparedness; made four postnatal home visits to negotiate preventive care practices and to assess newborns for illness; and referred sick neonates to a hospital and facilitated compliance. Primary outcome measures were antenatal and immediate newborn care behaviours, knowledge of danger signs, care seeking for neonatal complications, and neonatal mortality.

**Findings:**

A total of 4616 and 5241 live births were recorded from 9987 and 11153 participants in the intervention and comparison arm, respectively. High coverage of antenatal (91% visited twice) and postnatal (69% visited on days 0 or 1) home visitations was achieved. Indicators of care practices and knowledge of maternal and neonatal danger signs improved. Adjusted mortality hazard ratio in the intervention arm, compared to the comparison arm, was 1.02 (95% CI: 0.80–1.30) at baseline and 0.87 (95% CI: 0.68–1.12) at endline. Primary causes of death were birth asphyxia (49%) and prematurity (26%). No adverse events associated with interventions were reported.

**Conclusion:**

Lack of evidence for mortality impact despite high program coverage and quality assurance of implementation, and improvements in targeted newborn care practices suggests the intervention did not adequately address risk factors for mortality. The level and cause-structure of neonatal mortality in the local population must be considered in developing interventions. Programs must ensure skilled care during childbirth, including management of birth asphyxia and prematurity, and curative postnatal care during the first two days of life, in addition to essential newborn care and infection prevention and management.

**Trial Registration:**

Clinicaltrials.gov NCT00198627

## Introduction

Neonatal mortality declined by approximately 20% over the last decade in Bangladesh, however, the rate of decline was less than in the postneonatal and 1–4 year-old periods.[1], [2] Neonatal deaths now account for almost half of under-5 child deaths in Bangladesh and efforts to reduce neonatal mortality are crucial to achieving Millennium Development Goal 4 for child survival.[1]–[4] Since 90% of births and most neonatal deaths still occur at home,[1], [3] community-level interventions must be introduced while linking with the healthcare system for treatment of life-threatening newborn illness.[5]–[9]

Several recent community-based trials of packages of maternal and neonatal interventions in low resource settings in South Asia have shown statistically significant reductions in neonatal mortality, employing a variety of healthcare delivery approaches. The focus of the interventions, however, has been primarily on averting deaths due to serious infections. Home-based health education and routine neonatal assessment and antibiotic treatment of serious infections by community health workers (CHWs) decreased mortality in rural India[10], [11] and rural northeastern Bangladesh,[12] although regulatory approval for and availability of CHWs for home-based treatment of illness is lacking in most settings. A preventive maternal and neonatal care behavior change management program implemented by CHWs through home visits as well as community mobilization also reported a mortality reduction of 54% in a very high mortality area of Uttar Pradesh, India.[13] Lady health workers in Hala, Pakistan, promoted essential maternal and newborn care through home visits, community education group sessions, and linkages with local traditional birth attendants (TBAs), resulting in a 28% reduction in mortality.[14] Studies without home-based interventions also reported mortality reductions of about 30% through community-based participatory interventions in Nepal[15] and by improving TBAs' clean delivery practices and strengthening their linkages with primary health facilities in Larkana, Pakistan.[16]

To provide cost-effective essential preventive and curative services in low-resource settings, strategies must take into account the risk factors for and causes of mortality, the quality and accessibility of the health care system, and community perception and acceptance of the interventions.[17], [18] Community-based preventive care coupled with basic management of childhood illness and facilitated referral by CHWs is a potentially effective model where access to quality health care at facilities can be ensured.[19] We developed a preventive service delivery strategy in a rural area of central Bangladesh with good access to facility-based care to promote household newborn care practices through home visits by CHWs, and conducted routine, home-based illness surveillance coupled with facilitated referral of sick newborns to health facilities. A cluster-randomized controlled trial was conducted to examine its impact on knowledge and practice of newborn care and neonatal mortality.

## Methods

The protocol for this trial and supporting CONSORT checklist are available as supporting information; see Checklist S1 and Protocol S1.

### Study Population and Design

**Figure 1:**
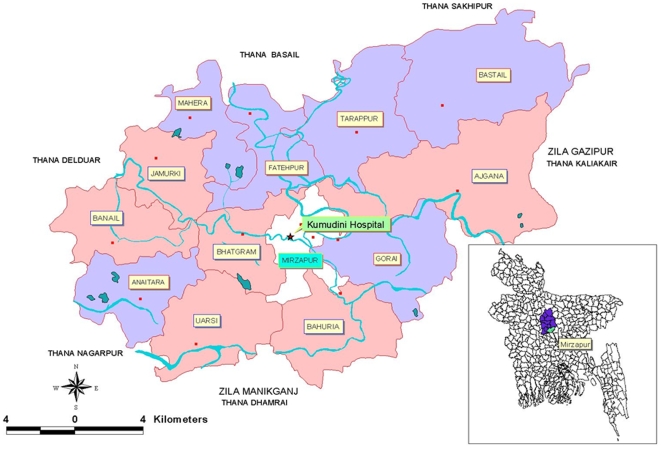
Distribution of study unions (clusters), Mirzapur sub-district, Tangail district, Bangladesh. Red circle: Union Head Quarter. Star: Kumudini Hospital. Light blue line: River/Beel. Pink shade: Intervention Area, Purple shade: Comparison Area.

Projahnmo-Mirzapur was a cluster-randomized, controlled intervention trial of a preventive and curative maternal-neonatal healthcare package, in which was nested surveillance for community-acquired neonatal bacteremia.[20]. The trial was implemented in Mirzapur, a sub-district of Tangail district, Dhaka division, Bangladesh, located 2 hours by car from the capital city of Dhaka, during January 2004– December 2006. The neonatal mortality rate (NMR) was estimated at 24 per 1000 live births in 2002. The area was served by Kumudini Hospital – a 750-bed, private, referral-level hospital, located in a central urban union which was excluded from the study. The remaining population of about 292,000 was divided into 12 rural unions, which were randomly allocated to either comparison or intervention arm using a computer-generated pseudo-random number sequence without stratification or matching (Figure 1). Blinding was unachievable given the nature of the intervention. Newborns in the comparison arm received the usual health services provided by the government, non-governmental organizations and private providers. In the intervention arm, each union had six CHW areas, each of which consisted of approximately 4000 population served by one CHW. The CHW-to-population ratio was similar to the primary healthcare worker-to-population ratio in the Bangladesh government health system, thus facilitating sustainability and scalability of the healthcare delivery strategy.[12] All married women of reproductive age (i.e., 15–49 years) in the intervention arm were eligible for enrolment, and were administered informed verbal consent by the CHW in their area.

### Design and Implementation of Interventions

Community-level interventions were developed based on findings from formative research on newborn care practices in the study population, conducted during November 2002– April 2003. Information on pregnancy, delivery, immediate newborn care and care seeking for newborn illness was collected through 26 unstructured interviews with women, husbands, mothers-in-law, and TBAs, and through semi-structured, in-depth interviews with 40 women and/or family members and 54 healthcare providers, including TBAs, health workers of BRAC or village associations, and village doctors.[21] Findings from formative research were used to design the communications and negotiation approach to promote safe and clean delivery and preventive, household newborn care practices (Table 1).

**Table 1:** Antenatal (birth and newborn care preparedness) and postnatal interventions at home by community health workers.

**PRENATAL: Two home visits scheduled at 12–16 weeks and 32–34 weeks to:**
1. Promote antenatal care, including:
(1) Making three antenatal care visits from a health centre or a satellite clinic
(2) Receiving two doses of tetanus toxoid vaccine
(3) Procuring adequate iron-folic acid (IFA) supplementation
(4) Eating extra food
(5) Care seeking for the following maternal danger signs:
- Prolonged labor
- Hemorrhage
- Fever
- Convulsion
- Edema of the face, hands or legs, or
- Blurred vision
2. Promote birth planning, including:
(1) Planning for delivery at a health facility
(2) If facility is not feasible, choosing a trained birth attendant; preparing the site of delivery in the house; obtaining birth kit or boiling the blade and the pieces of thread; planning for emergency transport; and saving money for emergency
3. Distribute: clean delivery kit, obtained from Bangladesh Rural Advancement Committee (NGO) free-of-charge, at the second antenatal visit for use by birth attendant
4. Promote newborn-care preparedness, including:
(1) Choosing a household member to take care of the newborn right after birth
(2) Drying and wrapping the baby from head to toe soon after delivery and before the delivery of placenta; using 2 pieces of cloth to wrap the newborn; holding the baby at all times during and immediately after the delivery; avoiding any contact of the newborn with the floor; not keeping the newborn in an unclean or cold place; applying gentle stimulation or refer for resuscitation of the newborn if he/she does not breathe immediately after birth; and practicing wrapping the baby using a doll during CHW visits
(3) Feeding colostrum to the newborn; initiating breastfeeding immediately after birth; practicing exclusive breastfeeding up to six months; and feeding the newborn frequently in the proper position day and night
(4) Delaying bathing of the newborn for 72 hours
(5) Umbilical area care: keeping the cord clean and dry; and avoiding applying anything to the umbilical stump
(6) Monitoring the baby for signs of infection; and seeking care immediately from CHW or health facility if the newborn has any of the following danger signs:
- No cry or breathing at birth,
- Convulsions
- Unconsciousness
- Difficulty breathing
- Feeling hot or cold to the touch
- Skin pustules or blisters
- Umbilical pus or redness
- Weak, abnormal or absent cry
- Lethargic or less than normal movement
- Yellow colour of the body, or
- Feeding problem
**POSTNATAL: Four home visits on postnatal days 0, 2, 5, and 8 to:**
1. Reinforce newborn care messages provided through prenatal visits
2. Provide counseling for routine breastfeeding and for breastfeeding difficulties
3. Surveillance of newborn illness: Identify sick neonates based on a clinical algorithm.[23] For identified sick neonates, recommend referral-level evaluation at Kumudini hospital or, if referral fails, continue monitoring according to the clinical algorithm.

CHWs were trained for 36 days on pregnancy surveillance, counseling and negotiation skills, essential newborn care, neonatal illness surveillance and management of illness based on a clinical algorithm adapted from Integrated Management of Childhood Illness. After initial training and evaluation, routine monitoring and refresher training were provided each fortnight.[22] Further information on recruitment, characteristics, training and monitoring of CHWs is presented elsewhere.[22] In addition, TBAs serving in the intervention unions (n = 84) attended a two-day orientation session on the aims and activities of the project, essential newborn care practices, and indications for referral of newborns and mothers.

Table 1 presents detailed information on interventions provided by CHWs in the intervention arm. CHWs identified pregnancies in their population through bimonthly household pregnancy surveillance. Birth and newborn care preparedness (BNCP) was promoted by CHWs through two antenatal home visits scheduled at 12–16 and 32–34 weeks of gestation. CHWs gave a labor notification card to each woman with instructions for a family member to seek out and present the card to the CHW when the pregnant woman started into labor. CHWs, notified by the card, attended the delivery whenever possible, or visited the mother and newborn infant as early as possible in the postnatal period. CHWs conducted three additional postnatal visits on days 2, 5 and 8 to promote preventive newborn care practices and to identify and refer sick neonates to Kumudini Hospital. During each of the postnatal visits, CHWs completed a standardized newborn assessment form, identified the presence of serious illnesses requiring referral to Kumudini Hospital – including illness indicative of infections, potentially requiring antibiotic treatment – and made referral to the hospital according to the clinical algorithm.[22] CHWs' classification of neonates with illness had high validity compared to physicians' classification.[22] Use of the clinical algorithm by CHWs during routine household surveillance was also validated in identifying severely ill neonates needing urgent referral to the hospital and those who subsequently died.[23] To eliminate potential barriers to care seeking for illness,[24] CHWs facilitated transport, if necessary, for neonates needing referral-level evaluation at Kumudini Hospital, and all care at the hospital was free-of-charge for referred neonates. The mean travel time to the hospital was about one hour,[22] and formative research suggested positive community perception of the quality of care at the hospital.[25] If the family refused to be referred, the CHW continued to encourage referral but managed the neonate in the home according to the algorithm, without use of injectable antibiotics.[22]

### Data

In order to examine intervention effectiveness, baseline and endline surveys were conducted in both study arms, using comparable questionnaires. Primary outcome measure was neonatal mortality; secondary outcomes included antenatal and immediate newborn care behaviours, knowledge of danger signs, and care seeking for neonatal complications. The surveys included all households with recently delivered women (RDW) (i.e., women who had a pregnancy outcome in the three calendar years before the survey), and collected household wealth and basic demographic information from all household members. To measure the mortality outcome, a hypothesized 40% reduction during the intervention period, a total sample size of 14,872 neonates was required, based on the baseline NMR of 28 per 1000 live births, power of 80% and an estimated design effect of 2.55 derived from the baseline data. Given a crude birth rate of 27 per 1000 population, 7884 live births were expected per year, and the surveys collected life-time pregnancy history from all eligible RDW at both baseline and endline. We anticipated that, after three months of initial intervention scale-up, a two-year period of enrolment during which the implementation of the intervention was stabilized would be sufficient. In addition, for all identified neonatal deaths during a defined period (see below), verbal autopsy data, including signs and symptoms of illness leading to deaths, were collected by separate interviewers who were trained in verbal autopsy data collection for six days.

To measure indicators of care practice and knowledge, the surveys also collected knowledge (K) of maternal and newborn care practices, household practice (P) of maternal-newborn preventive and curative care behaviours; and program coverage (C) among RDWs who had a pregnancy outcome in the last 12 months before each survey, hereafter referred to as KPC-RDW. At baseline, all eligible KPC-RDW were interviewed, while a sample of KPC-RDW was interviewed during the endline survey. The endline sample of KPC-RDW was randomly selected within each union, based on a sample size calculation to provide estimates for all KPC indicators assuming 50% prevalence with ±6% precision and response rate of 85% for each union.

Baseline household listing and mapping was conducted during March – June 2003, and households with at least one RDW who had a pregnancy outcome between 2000 and 2002 were identified. The baseline survey was conducted during April – July 2003. Response rates were 86.9% (14532/16725) among all RDW and 92.4% among all KPC-RDW (4636/5015). Verbal autopsy data were collected during September – December 2003, on average 14.8 months (SD: 3.6, n = 109) after the death, for neonatal deaths among those born in 2002 (response rate 88.6%, 109/123). The intervention was introduced and scaled up to the entire study area during December 2003– February 2004. Implementation continued through December 2006. Endline enumeration of households with RDW who had a pregnancy outcome between 2003 and 2005 was conducted during December 2005– April 2006. The endline survey was conducted during January – May 2006, before the end of the trial, in order to maintain community cooperation and minimize potential end-of-project effect in outcome measurement. The response rate was 87.8% (14731/16771) among all RDW. The KPC-RDW response rate was 94.0% (3519/3744). For all neonatal deaths among those born during the intervention period (2004–2005), verbal autopsy information was collected during April – August 2006 (response rate 86.4%, 222/257). The mean interval between a death and verbal autopsy data collection was 16.5 months (SD: 8.1, n = 222).

In addition, two interim adequacy surveys of knowledge and practice were conducted to monitor the coverage or adequacy of the intervention, and to guide adjustments in the implementation to optimize coverage and quality of the intervention. Random samples of households were selected from the baseline household listing for the two adequacy surveys. Sample size was calculated to provide estimates for selected KPC indicators, assuming 50% prevalence, ±10% precision, and response rate of 85% for each union. The first and second adequacy surveys were conducted during December 2004– January 2005 and August – September 2005, respectively. In total, 1141 and 1213 women who had a pregnancy outcome in the 12 months before each survey were enumerated in the first and second adequacy surveys, respectively. Response rates were 82.7% (1141/1380) for the first, and 86.5% (1194/1380) for the second adequacy survey. Informed verbal consent was administered by survey interviewers for all participants.

### Statistical Analysis

We analyzed the two adequacy surveys and the endline survey to estimate coverage changes in three consecutive 8-month periods in the intervention arm. Analyses were restricted to pregnancies which ended during the following 8-month periods, to avoid overlap between surveys: April – November 2004 (from adequacy survey 1), December 2004– July 2005 (from adequacy survey 2), and August 2005– March 2006 (from the endline survey). Coverage of the program was assessed in three areas: antenatal (whether a CHW visited the home at least once during pregnancy), delivery (whether a CHW attended at delivery), and postnatal (whether a CHW assessed a neonate at least once within the first 2, 7, and 28 days of life, respectively, and, among those who received postnatal visits, the mean time of first visit and the mean number of visits).

The baseline and endline surveys were analyzed to assess changes in three main outcomes in both comparison and intervention arms: reported maternal and newborn care practices, knowledge of maternal and newborn danger signs of illness, and neonatal mortality, controlled for basic demographic and socioeconomic characteristics. We first estimated means and proportions of RDW with selected background characteristics, by study arm and survey, including mother's age at birth (<20 years, 20–29 years, and ≥30 years), mother's educational attainment (< primary school completion *vs.* ≥ primary school completion), and household wealth status. A household wealth index score, based on the pooled data of baseline and endline surveys, was constructed using principal component analysis of household assets.[26] Households in each survey were ranked based on the index score and categorized into quintiles. The lowest and highest quintiles were classified as poor and rich, respectively, relative to the three middle quintiles.

Antenatal and neonatal care practices were measured among KPC-RDW. The last pregnancy was used as an index pregnancy if there were two or more pregnancies within the 12-month recall period. A woman was considered to have received routine antenatal care from a qualified provider (distinct from BNCP home visits by CHWs) if she had received ≥1 antenatal check-up either at a medical facility (i.e., satellite clinic, Union Health and Family Welfare Centre – a primary health facility serving approximately 20,000 population in the union, Upazila health complex – a first-level referral public hospital in each sub-district, qualified doctor's chamber, clinic or hospital) or by a qualified provider (i.e., doctor, nurse, Family Welfare Visitor – health personnel at a Union Health and Family Welfare Centre, or medical assistant). Among all home-born live births, seven selected immediate essential newborn care variables were measured, including sterile cord cut (i.e., the cord was cut by either a blade which was boiled before use or a blade from a clean delivery kit); drying/wiping the baby before delivery of the placenta; wrapping the baby before delivery of the placenta; delaying the first bath to the third day of life or later; initiating breastfeeding within one hour after delivery; breastfeeding prior to giving any food or liquid; and not applying anything to the cord immediately after cutting and tying it. Care seeking to a qualified provider (defined above) was measured among all neonates who had signs of complications based on maternal report. The baseline and endline surveys collected information on 11 and 19 complication signs, respectively, and we restricted analyses to neonates with ≥1 of 10 signs collected in both surveys (Table 2).

**Table 2:** Definition of neonatal complications used to measure conditional care seeking: baseline and endline survey.

Baseline survey*	Endline survey†
1. Fever	1. Fever (temp more than 101F)
2. Trouble breathing	2. Difficulty in breathing or fast breathing‡
3. Jaundice	3. Jaundice
4. Diarrhea	4. Diarrhea
5. Umbilical infection or discharge	5. Pus in the umbilicus or redness of the umbilicus‡
6. Convulsion	6. Convulsion
7. Stopped breast feeding	7. Poor feeding or unable to suck
8. Body became excessive cold	8. Hypothermia (temp 95.5–97.5 F)
9. Retention of urine	9. Doesn't pass urine
10. Unconsciousness	10. Unconscious

*Persistent vomiting was included in the baseline survey.

† Following additional signs were included in the endline survey: (1) red eye/passage of pus from eyes, (2) skin lesion with infection, (3) baby doesn't cry/breath, (4) chest in drawing, (5) doesn't pass stool, (6) cold/cough, and (7) others.

‡ Listed as 2 separate signs in the survey questionnaire.

Knowledge of maternal and neonatal danger signs was measured among KPC-RDW, using unprompted binary knowledge variables of 10 antenatal, 11 childbirth, 9 postpartum maternal, and 16 neonatal danger signs (Table 3). Four composite knowledge score variables were constructed for antenatal (range [0–10]), childbirth (0–11), postnatal maternal (0–9) and neonatal danger signs (0–16), by adding un-weighted positive answers for each of the individual signs within the category. Composite variables were treated as having missing values if the respondent had not completed all questions in each category.

**Table 3:** Individual signs included in the prenatal, labor/delivery, and postpartum danger sign knowledge scores.

	Danger signs
**Prenatal**	1. Severe headache
	2. Blurred vision
	3. Fetal movement absent
	4. High blood pressure
	5. Edema of the face/swelling
	6. Edema of the hands/leg swelling
	7. Convulsions
	8. Excessive vaginal bleeding
	9. Severe lower abdominal pain
	10. Leaking fluid (meconium stained)
**Labor/delivery**	1. Excessive vaginal bleeding
	2. Foul-smelling discharge
	3. High fever
	4. Baby's hand or feet coming out first
	5. Baby is in abnormal position
	6. Prolong labor (>12 hours)
	7. Retained placenta
	8. Rupture uterus
	9. Cord prolapse
	10. Cord around neck
	11. Convulsion
**Postpartum**	1. Excessive vaginal bleeding
	2. Foul-smelling discharge
	3. High fever
	4. Inverted nipples
	5. Tetanus
	6. Retained placenta
	7. Severe abdominal pain
	8. Convulsions
	9. Engorged breasts/swelling of breasts
**Neonatal**	1. Poor feeding or unable to suck
	2. Diarrhea
	3. Redness around the cord
	4. Red eye/discharging eyes
	5. Difficult breathing
	6. Yellow coloration of the skin/jaundice
	7. Hypothermia/shivering
	8. Blisters on skin/Skin lesion
	9. Baby doesn't cry
	10. Fever
	11. Unconscious
	12. Fast breathing
	13. Chest indrawing
	14. Doesn't pass urine
	15. Doesn't pass stool
	16. Convulsions

One additional prenatal danger signs (high fever) and 1 additional newborn danger sign (cold/cough) were included in the endline survey. We excluded those in creating the knowledge scores in order to maintain comparability across surveys.

To investigate differential changes in knowledge and practices, we conducted intention-to-treat analyses at the study arm level, using difference-in-difference test with interaction terms for time (baseline *vs.* endline) and study arm (comparison *vs.* intervention). We estimated predicted mean of each knowledge or practice indicator by time and study arm and compared the change between baseline and endline by study arm, controlling for maternal and household background characteristics described above. Linear probability regression models were used to test the null hypothesis that the difference-in-difference was zero.[27] Robust standard errors were adjusted for clustering on each union.

Neonatal mortality was examined using pregnancy history by all RDW. We assessed mortality data quality by examining distributions of the monthly number of live births, the monthly number of neonatal deaths, and age at deaths by year. We included live births in two-calendar-year-periods, January 2001– December 2002 from the baseline survey and January 2004– December 2005 from the endline survey, to control for the potential seasonal effect on mortality and to eliminate the 11-month pre-intervention period (January – November 2003) included in the 3-year pregnancy history recall period for the endline survey. We estimated NMR and 95% confidence intervals (CI) by time and study arm. We used a survival-time model with a Weibull survival distribution to estimate relative hazard of mortality between the study arms at baseline and at endline, adjusted for child sex and background characteristics described above. Robust standard errors adjusted for clustering on each union.

Finally, verbal autopsy data were analyzed to estimate cause-specific neonatal mortality rate by time and study arm, using neonatal deaths among those born in 2002 (baseline), and in 2004–2005 (endline). We applied a standard hierarchical algorithm to assign one primary cause out of the seven major causes of neonatal mortality in the order of: congenital malformation, tetanus, preterm birth, birth asphyxia, birth injury, sepsis or pneumonia, and diarrhea.[28]

A p-value of 0.05 was considered statistically significant, and all analyses of KPC indicators were adjusted for sampling weight. STATA 9.0 statistical software (Stata Corporation, College Station, TX, USA) was used for all analyses. The study was approved by the Committee on Human Research at the Johns Hopkins Bloomberg School of Public Health, the Ethical Review Committee and the Research Review Committee at ICDDR,B, the Ethical Review Committee at Dhaka Shishu Hospital and the Ethical Review Committee at Oxford University. The study was registered at clinicaltrials.gov, No. NCT00198627.

## Results

### Enrolment

**Figure 2:**
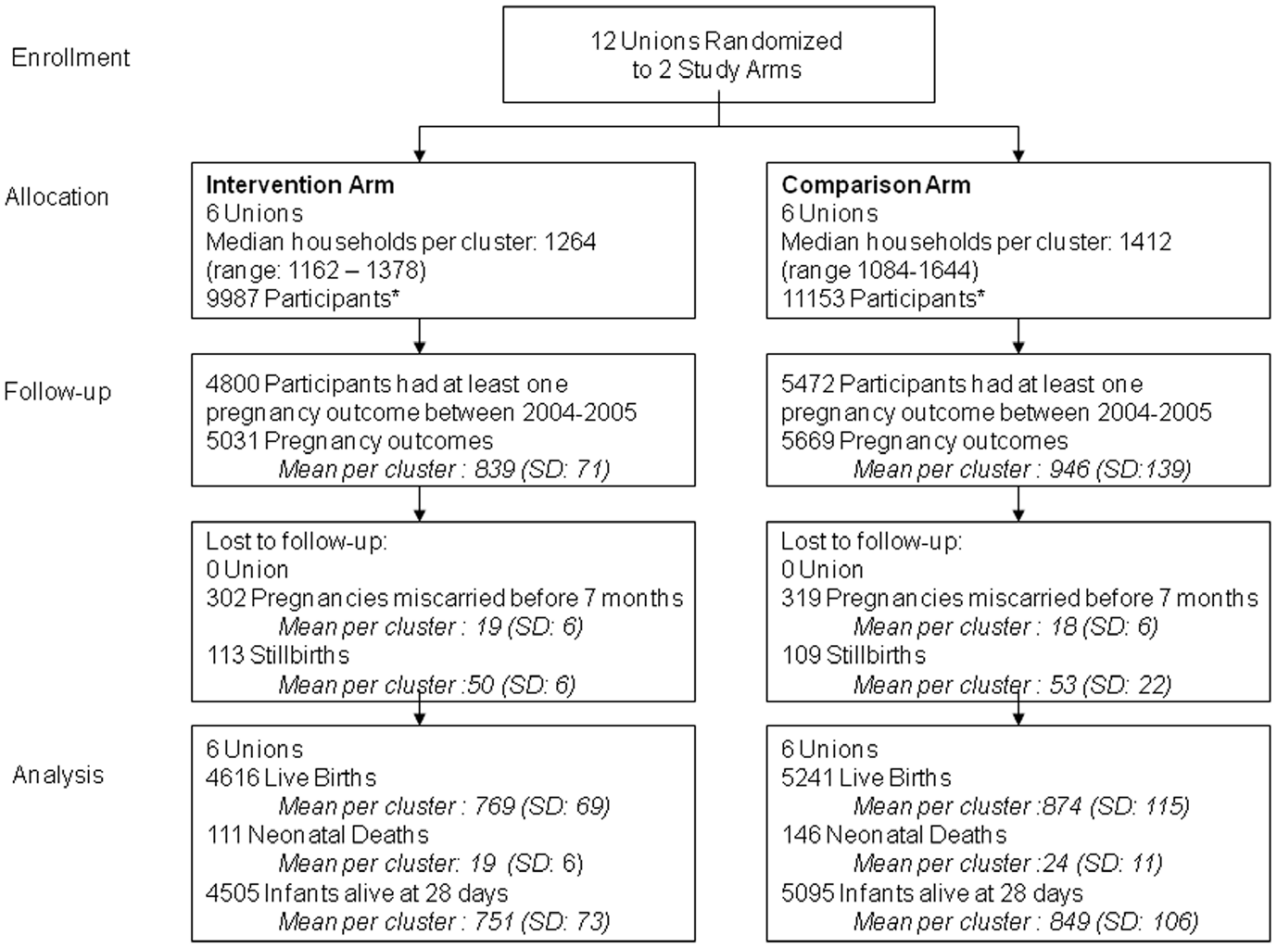
Trial profile for measurement of neonatal mortality. *Participants are women of reproductive age (15–49).

A total of 9987 women of reproductive age had 5031 pregnancy outcomes in the intervention arm, including 302 miscarriages, 113 stillbirths and 4616 live births (Figure 2). In the comparison arm, 11153 women of reproductive age had 5669 pregnancy outcomes, including 319 miscarriages, 109 stillbirths and 5241 live births. There were no differences in the rates of miscarriage and stillbirth between the two arms. Enrolment rates did not vary across unions.

### Coverage of the Intervention

In the intervention arm, percent of pregnant women receiving ≥1 BNCP visit reached over 90% during the first survey period (April – November 2004) and remained comparable in the subsequent two 8-month survey periods (Table 4). Similar rates were seen for receipt of two BNCP visits. Percent of home-deliveries attended by CHWs was 12% during April – November 2004, increased to 20% during December 2004– July 2005, but remained at 14% during August 2005– March 2006. Percent of home-born newborns assessed by CHWs within the first two and seven days of life improved from 54% to 69% and from 66% to 74%, respectively, over the survey periods. Among those who were assessed by CHWs at least once during the first 28 days of life, the average timing of the initial assessment decreased and the mean number of assessments increased over the periods.

**Table 4:** Changes in program coverage by community health workers in the intervention arm, among women who had a pregnancy outcome in the 8-month period before each survey.

Service	Adequacy survey 1		Adequacy survey 2		Endline survey	
	(Apr 2004–Nov 2004)		(Dec 2004–Jul 2005)		(Aug 2005–Mar 2006)	
**Antenatal BNCP, among all pregnant women**	(N = 565)		(N = 564)		(N = 1096)	
Home visit at least once	91.3	(89.0–93.7)	87.0	(84.2–89.8)	93.0	(91.5–94.6)
Home visits twice	86.9	(84.1–89.7)	83.8	(80.8–86.9)	91.0	(89.3–92.7)
**Delivery, among all women who delivered at home**	(N = 447)		(N = 385)		(N = 800)	
Labor notification to CHWs	28.8	(24.6–33.0)	44.4	(39.4–49.3)	34.8	(31.5–38.1)
Delivery attendance by CHWs	12.0	(9.0–15.0)	20.0	(16.0–24.0)	13.8	(11.4–16.2)
**Postnatal, among all women who delivered live births at home**	(N = 433)		(N = 379)		(N = 790)	
Home visits at least once during postnatal day 0–27	75.5	(71.4–79.5)	83.7	(79.9–87.4)	79.7	(76.9–82.5)
Home visits at least once during postnatal day 0–6	65.6	(61.1–70.1)	77.7	(73.4–81.9)	73.8	(70.7–76.9)
Home visits at least once during postnatal day 0–1	53.6	(48.8–58.3)	73.6	(69.1–78.1)	69.1	(65.9–72.4)
Time of first home visit (day)†	2.4	(1.9–3.0)	1.6	(1.0–2.2)	1.5	(1.1–1.8)
Total number of home visits†	2.6	(2.5–2.8)	2.8	(2.6–3.0)	3.2	(3.0–3.3)

BNCP: Birth and newborn care preparedness; CHWs: Community health workers.

†Among those who had at least 1 postnatal home visit during postnatal days 0–27: n = 325 (Adequacy survey 1); n = 318 (Adequacy survey 2); and n = 628 (Adequacy survey 1).

### Practice

Indicators of maternal and newborn care practice and knowledge were similar in the intervention and comparison arms at baseline. Adjusted for significant improvement in background socioeconomic characteristics in each arm (Table 5), proportions of women who received ≥1 routine antenatal check-up (distinct from antenatal BNCP visits by CHWs) from a qualified provider and took antenatal iron supplements increased significantly in the intervention arm (reaching 69% and 56%, respectively), but not in the comparison arm (49% and 43%, respectively) (Table 6). There was no change in the proportions of women who received ≥1 tetatus toxoid immunization during pregnancy in either study arm (approximately 75%), however, the proportion of women who received ≥2 tetanus toxoid immunizations during pregnancy decreased in both study arms (from about 55% to about 40%), likely associated with national shortage of the vaccine.[29]

**Table 5:** Maternal demographic and household economic characteristics by study arm and time, among all women had live births between 2001–2002 (baseline) and between 2004–2005 (endline).

	Comparison				Intervention			
	Baseline		Endline		Baseline		Endline	
	(n = 5166)		(n = 5143)		(n = 4822)		(n = 4498)	
	%	(95% CI)	%	(95% CI)	%	(95% CI)	%	(95% CI)
**Mother's age at birth (year)**								
mean	25.2	(25.0–25.3)	25.0	(24.8–25.1)	25.4	(25.2–25.5)	25.3	(25.1–25.4)
<20 years	14.4	(13.4–15.4)	15.6	(14.6–16.6)	12.7	(11.7–13.6)	13.1	(12.2–14.2)
≥30 years	20.2	(19.1–21.3)	17.9	(16.9–19.0)	20.3	(19.2–21.4)	18.7	(17.6–19.9)
≥35 years	6.2	(5.6–6.9)	6.5	(5.8–7.2)	6.3	(5.7–7.0)	6.5	(5.8–7.3)
**Maternal education**								
Ever attended school	61.7	(60.4–63.1)	72.4	(71.1–73.6)	64.7	(63.3–66.1)	75.6	(74.4–76.9)
Completed primary school	48.9	(47.5–50.3)	58.1	(56.7–59.4)	52.0	(50.6–53.5)	62.5	(61.1–63.9)
Completed high school	14.6	(13.6–15.6)	20.0	(18.9–21.1)	16.5	(15.4–17.6)	21.9	(20.7–23.1)
**Household wealth** *								
Wealth index score	−0.19	(−0.2–0.1)	0.44	(0.4–0.5)	−0.36	(−0.4–0.3)	0.44	(0.4–0.5)
Poor	22.7	(21.6–23.9)	15.0	(14.0–16.0)	25.7	(24.5–27.0)	14.7	(13.7–15.8)
Rich	17.6	(16.6–18.7)	26.2	(25.0–27.4)	15.7	(14.6–16.7)	26.5	(25.2–27.8)

*Wealth index created based on pooled baseline and endline surveys, using principal component analysis of durable goods, electricity, toilet facility, sources of drinking water, and housing materials.[26] Poor refers to the lowest quintile of the wealth index and rich is the highest quintile of the wealth index.

**Table 6:** Adjusted predicted mean of knowledge and practice indicators by study arm and time, among women who had a pregnancy outcome in the 1-year period before each survey.*

	Comparison		Intervention		
	Baseline	Endline	Baseline	Endline	
	(May 2002– Jul 2003)	(Feb 2005– Apr 2006)	(May 2002– Jul 2003)	(Feb 2005–Apr 2006)	
**PRACTICE (percent of target population practicing a behavior)**					
**Prenatal care and birth preparedness among all women**					
*Number of women*	*2644*	*1759*	*2371*	*1732*	
Had ≥1 antenatal care visits from a qualified provider†	47.8	49.1	47.4	68.8	∥
Received ≥1 tetanus immunization	76.4	73.8	77.1	77.5	
Received ≥2 tetanus immunizations	56.9	41.0	54.7	39.8	
Received iron supplementation	45.8	42.7	47.9	55.7	∥
**Facility-based delivery**					
*Number of women*	*2644*	*1759*	*2371*	*1732*	
Delivered at medical facilities	12.5	16.5	12.1	20.2	∥
**Immediate newborn care among all home-born live births**					
*Number of home-born live births*	*2006*	*1322*	*1805*	*1231*	
Sterile cord cut‡	59.2	66.9	63.3	95.1	∥
Not applying anything on the newly-cut cord	95.1	86.0	94.8	94.3	∥
Drying/wiping the baby before delivery of placenta	2.1	3.0	2.2	14.4	∥
Wrapping the baby before delivery of placenta	2.4	2.7	2.9	13.5	∥
Delaying bath to the 3rd day or later	1.5	13.4	1.6	77.8	∥
Breastfeeding initiation within 1 hour after birth	41.2	55.0	40.9	80.0	∥
Breastfeeding prior to any food/liquid	28.9	50.5	29.3	87.3	∥
**Care seeking among neonates with complications**					
*Number of neonates with 1 or more of the 10 complications* §	*812*	*400*	*733*	*355*	
Received any treatment	93.7	95.9	92.9	97.3	
Received treatment from a qualified provider†	27.4	34.6	30.7	55.7	∥
**KNOWLEDGE (mean danger sign knowledge scores) [range]**					
*Number of women*	*2644*	*1759*	*2371*	*1732*	
Maternal danger sign knowledge score: antenatal [0–10]	1.0	2.2	1.1	2.9	∥
Maternal danger sign knowledge score: labor/delivery [0–11]	1.1	1.9	1.2	2.4	∥
Maternal danger sign knowledge score: postpartum [0–9]	1.0	2.0	1.0	2.5	∥
Neonatal danger sign knowledge score [0–15]	2.3	2.4	2.3	2.8	∥

*Adjusted for mother's age at birth (<20 years, 20–29 years (reference group), or ≥30 years), maternal educational attainment (<primary school completion vs. ≥ primary school completion), and the household wealth (the lowest quintile, middle 3 quintiles (reference), or the highest quintile of the wealth index), using linear probability regression models.

† Includes check-up/treatment by a qualified provider (e.g., doctor, nurse, family welfare visitor, or medical assistant) or at a medical facility (e.g., satellite clinic, Family Welfare Centre, Upazila health complex, qualified doctor's chamber, clinic, or hospital).

‡ Blade from a clean delivery kit or other instrument that was boiled before use.

§ See Table 2 for the list of complications.

∥ Significant differential change over time by study arm (i.e., significant interaction) (p-value <0.05).

Percent of women who delivered at a health facility remained small, but increased significantly more in the intervention (from 12 to 20%) than the comparison (from 13% to 17%) arm (Table 6). Among all home-born neonates, sterile cord cut, delaying the first bath, early breastfeeding initiation and breastfeeding before any food or liquid increased in both arms, but the increases were substantially larger in the intervention arm than in the comparison arm, reaching about 80% or more (Table 6). Immediate drying and immediate wrapping of the baby improved only in the intervention arm, reaching about 14%. Finally, among neonates who had ≥1 of the 10 selected complication signs, care seeking from a qualified provider increased significantly more in the intervention arm (from 31% to 56%) than in the comparison arm (from 27% to 35%).

### Knowledge

Unprompted knowledge of maternal and neonatal danger signs increased significantly in both study arms between the baseline and endline, adjusted for improvement in background socioeconomic characteristics (Table 5). However, the improvements in the intervention arm were significantly larger than those in the comparison arm (Table 6). Nevertheless, intervention-arm women identified only about three signs among 15 neonatal danger signs at the endline, and recognition improved only in selected individual neonatal signs, including redness around or discharge from the umbilicus, body cold/shivering, skin lesions, and convulsion (results not shown).

### Mortality

NMR estimates did not vary significantly by time or study arm (Table 7). NMR was 24.8 (95% CI: 20.7–29.4) and 27.9 (95% CI: 23.5–32.8) in the comparison arm at baseline and endline, respectively, and was 25.2 (95% CI: 21.0–30.1) and 24.0 (95% CI: 19.8–29.0) in the intervention arm at baseline and endline, respectively. Adjusted mortality hazard ratio in the intervention arm, compared to the comparison arm, was 1.02 (95% CI: 0.80–1.30) at baseline and 0.87 (95% CI: 0.68–1.12) at endline. Verbal autopsy data ascertainment rate did not vary significantly by sex, age at death, or study arm (results not shown). Cause-specific neonatal mortality rates did not differ by time or study arm. The most common causes of death during the intervention period (2004–2005) in the intervention and comparison areas combined were birth asphyxia (109/222, 49%), prematurity (58/222, 26%) and infection (26/222, 12%) (Table 7).

**Table 7:** Levels and causes of neonatal mortality by time and study arm.

		Baseline				Endline		
		Comparison		Intervention		Comparison		Intervention
**LEVELS**								
Period (calendar years)		2001–2002		2001–2002		2004–2005		2004–2005
Number of live births	5292		4951		5241		4616	
Number of deaths	131		125		146		111	
Neonatal mortality rate (per 1000 live births) [95% CI]	24.8	[20.7–29.4]	25.2	[21.0–30.1]	27.9	[23.5–32.8]	24.0	[19.8–29.0]
Adjusted hazard ratio* [95% CI]	1	(reference)	1.02	[0.80–1.30]	1	(reference)	0.87	[0.68–1.12]
**CAUSES, based on a hierarchical algorithm†**								
Period (calendar years)		2002		2002		2004–2005		2004–2005
Number of live births	2636		2463		5241		4616	
Number of deaths	72		51		146		111	
Number of deaths with complete verbal autopsy data (% of total neonatal deaths)	67	(93.1)	42	(82.4)	129	(88.4)	93	(83.8)
Number of deaths by cause (% of neonatal deaths with complete verbal autopsy data [95% CI])								
Congenital malformations	6	(9.0 [3.4–18.5])	4	(9.5 [2.7–22.6])	10	(7.8 [3.8–13.8])	0	(0.0 [0.0–3.9]) ‡
Tetanus	3	(4.5 [0.9–12.5])	2	(4.8 [0.6–16.2])	3	(2.3 [0.5–6.6])	1	(1.1 [0.0–5.8])
Preterm	16	(23.9 [14.3–35.9])	15	(35.7 [21.6–52.0])	33	(25.6 [18.3–34.0])	25	(26.9 [18.2–37.1])
Birth asphyxia	20	(29.9 [19.3–42.3])	11	(26.2 [13.9–42.0])	63	(48.8 [39.9–57.8])	46	(49.5 [38.9–60.0])
Birth injury	0	(0.0 [0.0–5.4]) ‡	0	(0.0 [0.0–8.4]) ‡	0	(0.0 [0.0–2.8]) ‡	3	(3.2 [0.7–9.1])
Infection	11	(16.4 [8.5–27.5])	8	(19.0 [8.6–34.1])	15	(11.6 [6.7–18.5])	11	(11.8 [6.1–20.2])
Diarrhea	0	(0.0 [0.0–5.4]) ‡	0	(0.0 [0.0–8.4]) ‡	1	(0.8 [0.0–4.2])	1	(1.1 [0.0–5.8])
Cause not assigned	11	(16.4 [8.5–27.5])	2	(4.8 [0.6–16.2])	4	(3.1 [0.9–7.7])	6	(6.5 [2.4–13.5])
Cause-specific neonatal mortality rate (per 1000 live births) [95% CI]								
Congenital malformations	2.3	[0.8–5.0]	1.6	[0.4–4.2]	1.9	[0.9–3.5]	0.0	[0.0–0.8] ‡
Tetanus	1.1	[0.2–3.3]	0.8	[0.1–2.9]	0.6	[0.1–1.7]	0.2	[0.0–1.2]
Preterm	6.1	[3.5–9.9]	6.1	[3.4–10.0]	6.3	[4.3–8.8]	5.4	[3.5–8.0]
Birth asphyxia	7.6	[4.6–11.7]	4.5	[2.2–8.0]	12.0	[9.2–15.4]	10.0	[7.3–13.3]
Birth injury	0.0	[0.0–1.4] ‡	0.0	[0.0–1.5] ‡	0.0	[0.0–0.7] ‡	0.6	[0.1–1.9]
Infection	4.2	[2.1–7.5]	3.2	[1.4–6.4]	2.9	[1.6–4.7]	2.4	[1.2–4.3]
Diarrhea	0.0	[0.0–1.4] ‡	0.0	[0.0–1.5] ‡	0.2	[0.0–1.1]	0.2	[0.0–1.2]
Cause not assigned	4.2	[2.1–7.5]	0.8	[0.1–2.9]	0.8	[0.2–2.0]	1.3	[0.5–2.8]

*Analysis of baseline and endline combined, stratified by study arm (n = 10553, intervention arm, and n = 9567, intervention arm). Mortality risk at baseline (2001–2002) was the reference group in each arm. Estimates were controlled for: sex, mother's age at birth [<20 years, 20–29 years [reference group]), or ≥30 years]), maternal educational attainment [<primary school completion vs. ≥ primary school completion]), and the household wealth [the lowest quintile, middle 3 quintiles [reference]), or the highest quintile of the wealth index]), using a survival-time model with a Weibull survival distribution.

† Causes are presented in the order of the hierarchy. Case definitions for the seven causes are presented elsewhere in detail.[28]

‡ One-sided 97.5% confidence interval.

## Discussion

This cluster randomized controlled trial of a package of maternal and newborn healthcare interventions successfully achieved good coverage of antenatal (∼90%) and postnatal (∼70%) home visits by CHWs, and significantly improved several key newborn care practices and care seeking for newborn complications from qualified providers. Knowledge of maternal and newborn danger signs also improved, although to a limited extent. However, there was no evidence for an impact of the intervention on neonatal mortality. These results are in contrast to several recent trials which decreased neonatal mortality in various settings in South Asia,[10], [12]–[16] and also contrasts with a large-scale program evaluation in rural India where lack of mortality impact seemed to stem from inadequate implementation and insufficient coverage of the interventions.[17] Our program coverage for both the antenatal and postnatal components was comparable with levels achieved in other effective trials.[12], [13] In addition, we had strict quality assurance of implementation through regular supervision of CHWs and through intensive monitoring of quality of program implementation through household “adequacy” surveys; data from the surveys was used to identify potential areas for improvement in program implementation, and to guide adjustments in intervention delivery to optimize program impact. In Sylhet, Bangladesh, we achieved a 34% reduction in mortality through a similar package of interventions, supervision and monitoring;[12] further analysis of that program revealed that a 64% reduction in mortality was seen among the newborns who were visited within the first two days of life whereas no mortality impact was found among those who were visited only after the two days.[30] Coverage of the first visit within the two days, however, was similar in Sylhet (62%) and Mirzapur (69%), and the magnitude of changes in care practices were also similar.[12], [30] Thus, factors other than reaching families with the intervention must be considered to explain the lack of mortality impact in this study and to guide future strategies to reduce mortality in moderate mortality settings such as Mirzapur.

Lack of evidence of mortality impact can be due to lack of power to test our hypothesis that the intervention would result in a 40% reduction in mortality in the intervention arm – a level of reduction that had been observed in other efficacy trials[10] and that we thought would be needed to compel policy and program change in Bangladesh. Given the lower number of live births in the study area than anticipated during study design, we did not achieve our enrolment target of 14,872 births. We speculated that a number of factors contributed to this, including declining fertility in rural areas of Bangladesh,[1], [31], [32] an overestimated initial population size, and potential omission of live births in the retrospective pregnancy history.[33] In particular, preliminary results from the Mirzapur Demographic Surveillance Systems since 2007[34] suggest that the initial population of 292,000 in 2003 was likely overestimated by about 18%, while the annual number of live births during 2004 and 2005 recorded in the retrospective birth history data was about 5% lower than the prospective demographic surveillance results. Expanding the study area or extending the intervention period would have been an option to achieve the target enrolment. However, the catchment area could not be extended in order to ensure access to Kumudini Hospital; and, there was no compelling reason to continue the trial longer than planned, due to the lack of evidence for a downward mortality trend in the intervention arm using program implementation data. In Sylhet, for example, a non-significant downward trend in mortality was observed within 6 month after the intervention started, and a significant program effect on mortality was observed 2 years after the initial intervention introduction.[12] In addition, improvement in care seeking for illness with qualified providers at Kumudini Hospital by families in both study arms, coupled with the provision of quality, life-saving care for any who reached the hospital, likely contributed to the lack of mortality impact of the intervention relative to the comparison area.

Most importantly, however, our results highlight that local epidemiology, including levels and causes of mortality in the community, must be taken into careful account during intervention design.[35], [36] As NMR decreases, particularly below about 30 per 1000 live births, the cause structure of mortality and, thus, the relative importance of various risk factors for mortality changes.[3]; [8] In most other community-based trials, baseline NMR exceeded 45 per 1000 live births,[10], [12]–[15] and serious infections, including sepsis, pneumonia, and tetanus, likely accounted for >40% of neonatal deaths.[3], [9], [28] Although our intervention was designed to address the major causes of mortality in neonates, it was most robust for the prevention and management of infections. In the Mirzapur population, however, nearly 60% of deaths were due to birth asphyxia or prematurity, and the program had limitations in reaching households at the critical times (i.e., during labour, childbirth and immediately after delivery) to address these conditions, and the CHWs lacked the necessary tools and skills to effectively address these conditions. CHWs attended <20% of home deliveries, largely due to difficulties in receiving timely notification of labour onset and in travelling to the home to intervene during delivery, given their population catchment area which extended over four villages and, particularly at night, strong discouragement from CHW families for travelling outside the village out of safety concerns. TBAs attended most home deliveries (97%) but in spite of brief but focused training in clean delivery, immediate newborn care, and danger sign recognition and referral, they lacked the capabilities to provide skilled care at birth, including resuscitation of birth asphyxiated newborns. Some evidence suggests, however, that TBA training in resuscitation is a potentially effective intervention.[37]–[40] Moreover, recent reviews and meta-analyses suggest that TBAs have some potential for promoting antenatal care, detecting obstetric complications, referring women to skilled obstetric care and positively impacting stillbirths and neonatal outcomes.[41], [42] We found, however, that the numbers and diversity of TBAs in the community made it challenging to train, supervise and manage them to uniform standards of care, and that TBAs and CHWs infrequently encountered a newborn that required bag-and mask resuscitation, which further complicates attempts to train and equip them to provide effective resuscitation in the community.[41] Current policy in Bangladesh does not promote TBA training programs, however, and implementation of newborn resuscitation outside health facilities is challenging.[2], [4] Thus, skilled attendance at delivery remains a key policy and program priority for reducing both neonatal and maternal mortality in Bangladesh.[2], [43], [44]

In addition to skilled care at delivery, early postnatal care is also critical for reducing mortality in moderate neonatal mortality settings, considering the preponderance of early deaths due to prematurity, birth asphyxia, and, to a lesser extent, vertically acquired sepsis.[20] Although our overall coverage of postnatal care was good, only 18% and 33% of neonates who died within the first day and the first week of life, respectively, were visited by CHWs prior to the death.[45] Moreover, among newborns who were assessed by CHWs and found to be ill, only 54% complied with referral to hospital and compliance with referral was 30% less likely in the first week of life,[45] despite attempts to eliminate major care seeking barriers – danger sign recognition, access to the hospital and cost.[46], [47] Thus, emphasis must be placed on community mobilization and empowerment,[48] and on greater understanding of and development of improved approaches to overcome social and financial barriers to referral compliance and care seeking at facilities, especially in the first week of life and in settings where cultural seclusion after birth remains a social norm.[47], [49], [50] Moreover, as NMR is reduced below about 30 per 1000, reliance on community-based care is likely to be inadequate to address the needs of extremely preterm infants, who often need additional interventions beyond essential newborn care interventions (e.g., breastfeeding, warmth and hygiene, and emollient therapy[51]–[54]), including corticosteroid administration to the mother prior to delivery, surfactant therapy at birth, and assisted ventilation such as continuous positive airway pressure.[3], [55], [56] Skilled attendance at facility-based deliveries, along with adaptation of these additional interventions for implementation in first-level facilities in low resource settings, can help to ensure their coverage.[48], [57] Emerging evidence suggests that in addition to understanding and overcoming social barriers to care seeking at facilities, programs to address financial barriers may also provide a powerful stimulus to families to access skilled care for delivery and immediate postnatal care at health facilities.[48], [58]

Finally, for treatment of serious neonatal infections, community-based case management is a viable alternative to facility-based care even where access to quality health care at facilities can be ensured. [5]–[12], [19], [22], [23], [46] In Sylhet, Bangladesh, while only 34% of referrals of sick newborns to hospital by CHWs were complied with, another 43% accepted injectable antibiotic treatment at home. Neonates in each treatment group had a significantly reduced hazard of mortality, compared to sick neonates who received no treatment or treatment from unqualified providers, indicating that with the addition of home-based treatment, approximately three-fourths of sick neonates received effective curative antibiotic treatment preventing death,[12], [59] a substantial improvement over what was achieved in Mirzapur, where we did not offer home-based treatment with injectable antibiotics.

In summary, for optimal survival improvement in low resource populations with moderate NMR, the intervention design must include a clear pathway to survival that links risk factors with causes of mortality, and identifies locally contextualized approaches to risk reduction.[13] As community-based interventions mature and NMR comes down, programs must ensure, in addition to essential newborn care; skilled care during childbirth, including interventions to prevent and manage birth asphyxia and respiratory distress syndrome in preterm infants; and high coverage of curative postnatal care in the first two days of life. Barriers to care seeking for illness must also be addressed. Where poor care seeking at referral-level hospitals exists during the early neonatal period, adaptation of interventions for extremely preterm infants for use at community clinic level must be prioritized, and consideration given to inclusion of home-based treatment of serious infections integrated into community case management strategies for childhood infections.

## Supporting Information

Checklist S1 Consort checklist
(0.06 MB DOC)

Protocol S1 Trial protocol
(0.15 MB PDF)

Reference S1 Supporting document: Reference 21 - unpublished report
(0.36 MB PDF)
