# Supplementary material for: Evaluation of a Cluster-Randomized Controlled Trial of a Package of Community-Based Maternal and Newborn Interventions in Mirzapur, Bangladesh
Source: PLoS One. 2010 Mar 24;5(3):e9696. doi: 10.1371/journal.pone.0009696 (PMC2844410; doi:10.1371/journal.pone.0009696)
Supplement: Protocol S1 — Trial protocol (0.15 MB PDF) [file pone.0009696.s002.pdf]

RESEARCH PROTOCOL

1. Title of Project: ETIOLOGY, PREVENTION AND TREATMENT OF NEONATAL INFECTIONS IN THE COMMUNITY

DESCRIPTION OF THE RESEARCH PROJECT

2. Specific Aims:

Describe the specific aims of the proposed study. State the specific parameters, biological functions/ rates/ processes that will be assessed by specific methods **(Type within limits).**

1 To identify the principal agents of serious bacterial infections in Bangladeshi neonates in the community.

2 To evaluate the impact of introducing a package of essential obstetric and neonatal care practices in the community, including identification of barriers to care-seeking and design of strategies to address those barriers.

3 To build capacity within Bangladesh by training Bangladeshi scientists in epidemiological and microbiological techniques, clinical research methods and best clinical practice through our on-going collaboration with Dhaka Shishu (Children) Hospital and the International Centre for Diarrhoeal Disease Research, Bangladesh (ICDDR,B).

3. Background of the Project Including Preliminary Observations:

Describe the relevant background of the proposed study. Discuss the previous related works on the subject by citing specific references. Describe logically how the present hypothesis is supported by the relevant background observations including any preliminary results that may be available. Critically analyze available knowledge in the field of the proposed study and discuss the questions and gaps in the knowledge that need to be fulfilled to achieve the proposed goals. Provide scientific validity of the hypothesis on the basis of background information. If there is no sufficient information on the subject, indicate the need to develop new knowledge. Also include the **significance and rationale** of the proposed work by specifically discussing how these accomplishments will bring benefit to human health in relation to biomedical, social, and environmental perspectives. **(Do not exceed 5 pages, use continuation sheets).**

Etiology of serious bacterial infections in neonates

Despite declines in infant mortality rates in recent decades, neonatal death rates (i.e., death in the first 28 days of life) remain unacceptably high. Of the 8 million infant deaths that occur worldwide each year, approximately 5 million occur in the neonatal period (WHO. 1996). An estimated 98% of these neonatal deaths occur in developing countries, principally in Asia and Africa. The first week of life is a particularly vulnerable period when 50 to 70% of fatal and life-threatening neonatal illnesses occur (Bartlett et al. 1991). An estimated 30 million of the annual birth cohort in developing countries acquire an infection, and approximately 1.8 million succumb, accounting for 30 to 40% of all neonatal deaths (Stoll. 1997).

Bangladesh is one of the most populated and poorest of the developing countries, and has among the highest perinatal mortality rates in the world (Arifeen SE. 1997; Baqui et al. 1998; Antelman. 1997). The risk of death in Bangladesh during the first month of life (48 per

1,000 births) is greater than in the next 11 months combined (34 per 1,000 births) (Mitra el al. 1996; Islam et al. 1982). Thus, about 40% of all under-five deaths or about 60% of all infant deaths occur in the first month of life. Overall, one in 20 children born in Bangladesh dies during the neonatal period.

Infectious diseases, along with birth asphyxia and prematurity are the principal causes of death in neonates worldwide (WHO. 1994). Our current knowledge of the etiology of infectious diseases in neonates in developing countries is based almost entirely on studies of hospitalized patients or on retrospective, verbal autopsy-based surveys in the community, neither of which may accurately reflect the true burden of disease in the community. While nation-wide surveys in Bangladesh have provided useful data on the cause of mortality for post-neonatal and childhood deaths, verbal autopsy was found to lack sufficient precision for assigning cause of death in a significant proportion of neonates (e.g., 15%) (Baqui. 1998). Nevertheless, infectious diseases were estimated to cause 35% of the neonatal mortality, with acute respiratory infection and tetanus causing 18% and 15% of deaths, respectively. Small hospital-based or retrospective community-based studies suggest that infectious mortality is due principally to acute respiratory infections (approximately 750,000 deaths per year), neonatal tetanus (375,000), sepsis (300,000), and diarrhea (150,000) (Bartlett et al. 1991; Stoll. 1997; WHO. 1994). A small, prospective, community-based study of early infant mortality in Guatemala categorically identified infectious diseases as the leading cause of lethal and potentially lethal illness (Bartlett. 1991). In a recent study in India, 52% of neonatal deaths were due to sepsis (Bang et al. 1999). The only large prospective study to date which precisely identified the etiologic agents of neonatal infections was conducted by the World Health Organization (WHO) in Ethiopia, The Gambia, Papua New Guinea, and The Philippines in 1990 to 1993 (WHO. 1999). This study was based on laboratory diagnostic testing in conjunction with medical history and clinical examination. Among 2398 young infants less than 90 days of age with suspected infection, 167 positive blood cultures were documented. The primary pathogens were *Staphylococcus aureus* (34), *Streptococcus pneumoniae* (33), and *Streptococcus pyogenes* (29). Most of the remainder were *Enterobacteriaciae*, represented by *Escherichia coli* (19), *Salmonella* (17), *Enterobacter* (7), and *Klebsiella* (5). Of the 43 positive cerebrospinal fluid (CSF) cultures, 17 were *S. pneumoniae*. This study, however, relied on evaluation of patients who self-referred to health care facilities, and, consequently, may not have provided a representative sample of agents of serious infectious diseases in the community.

No study has been reported which utilized community-based surveillance in conjunction with clinical and laboratory diagnostic techniques to precisely and prospectively determine the etiology or total burden of bacterial illness in neonates in a developing country. To develop strategies for improving child survival in early infancy, including rational empiric treatment regimens, a better understanding of causes of infectious morbidity and mortality is essential (Darmstadt et al. 2000). The only community-based surveillance project we are aware of is a Wellcome Trust-funded project in Kilifi, Kenya. Ours would be the first such investigation in Asia.

Community-based prevention and management of serious neonatal infections

An estimated 63% of infants are delivered at home in developing countries. In some countries such as Bangladesh, approximately 95% of births occur at home (Mitra et al. 1996). These deliveries often are conducted by traditional birth attendants (TBAs) or *dayas* who generally do not have adequate training in delivery care and management of the newborn. In addition, many mothers from rural communities are reluctant to seek care for their newborn at a health facility (Bartlett el al. 1991; Bang et al. 1999; Bang et al. 1993). Moreover, although the WHO recommends hospitalization of sick neonates (WHO. 1989; WHO. 1990), for many, facility-based care is inaccessible and/or unaffordable. As a result, most neonates die at home. Therefore, an important strategy for reducing neonatal mortality is community-based prevention of infectious diseases and early recognition and management of seriously ill neonates.

A prospective, home-based trial in rural India evaluated the efficacy of case management of neonatal pneumonia, including empiric oral antimicrobial therapy (Bang et al. 1993). In the intervention area, the adult population was educated in recognition of pneumonia and Community Health Worker s (CHWs) were trained in hygienic delivery practices, essential neonatal care, and diagnosis of pneumonia based on recognition of a few signs such as cough, respiratory rate $\geq$ 60/min, and chest indrawing. Neonates in the intervention area with suspected pneumonia were empirically given oral cotrimoxazole for 7 days. An attempt was made to refer complicated cases to a district hospital. The death rate among neonates in the intervention area who were diagnosed with pneumonia (17.4/1000) was decreased by 40% compared to neonates in the control area (29.1/1000). The reduction in pneumonia-specific mortality explained 57% of the reduction in neonatal mortality (from 83.9 to 63.6/1000). The authors concluded that neonatal pneumonia can be managed successfully in the community.

More recently, the home-based strategy of Bang et al. for reducing neonatal mortality in India was expanded to encompass treatment of suspected septicemia and meningitis as well as pneumonia (Bang et al. 1999). Community Health Workers in intervention areas were trained to provide a package of home-based neonatal care, including health education to pregnant women, diagnosis and management of birth asphyxia, identification of high-risk (premature and low birth weight) neonates for more intensive surveillance, temperature maintenance, promotion of breastfeeding, administration of vitamin K, treatment of umbilical cord stump and skin infections, and identification of sick newborns suspected of having septicemia, meningitis and/or pneumonia and administration of antibiotics [oral cotrimoxazole and intramuscular (IM) gentamicin] in the home. As a result of the implementation of this package of essential newborn care, mortality due to sepsis was reduced by 76% and neonatal mortality declined 62% compared to the control, non-intervention area at an estimated cost of $5.30 per neonate. Although promising, this study lacked the proper number of randomization units, having been conducted in only two areas which already had been serving as "control" and "action" (intervention) areas for many years prior to introduction of the newborn care package. Moreover, mortality rates in the control area varied year-to-year from 50 to 65 per 1000, rendering statistical inference and precise calculation of the effect-size problematic. There may also have been a strong pre-existing secular trend in the intervention area, which may have been obscured in the reported data by the grouping of data for 2 years in the baseline results. Therefore, the impact observed may have been a combination of effects due to the intervention and the secular trend. In addition, the principal investigators of this study had lived and worked in the study community for a decade prior to introducing the intervention, and the contribution of their presence to the impact of the intervention is difficult to assess. Therefore, the effects of the package of obstetric and newborn care on neonatal mortality observed by Bang et al. needs to be

demonstrated in other settings. We are planning to evaluate strategies similar to Bang et al. in another population (Sylhet) of Bangladesh.

However, the strategy of home-based treatment with antibiotics, especially parenteral administration, for life-threatening infections may be difficult for many countries to implement given the complexity and cost. Therefore, it is important to evaluate different health care delivery strategies for neonatal care, including the feasibility of referral to see if the same effect can be achieved with less costly strategies. Despite the apparent efficacy of home injectable antibiotic treatment, facility-based care has some advantages over home-based care in regards to administration of intravenous antibiotics, monitoring of clinical status and management of complications, and attention to nutritional needs.

Addressing barriers to care-seeking

Health-seeking practices and barriers to referral have been a major impediment to facility-based care of sick neonates (Ahmed et al. 2001; Zoysa et al. 1998). The "Three Delays" model is widely used for the planning of interventions to decrease maternal mortality (Barnes-Josiah et al. 1998). The model proposes that maternal mortality is mostly due to delays in: 1) deciding to seek appropriate medical help for an obstetric emergency; 2) reaching an appropriate obstetric facility; and 3) receiving adequate care when a facility is reached. This framework can be adapted for neonatal care interventions that aim to promote early and appropriate care for sick neonates at health facilities. Actions to promote delays at each level will include:

Table 1: Three "Delays" Model for Health Care Seeking Behavior for Neonates

**Where does delay occur**	**Examples of actions to address this delay**
Decision in the home to seek appropriate medical help	- Education of parents and community health workers about danger signs in neonates that require immediate care at a health facility<br>- Education about improvements to quality of care in health facility<br>- Institution of a system of referral slips for community health workers
Transport from home to facility providing appropriate care for neonates	- Establishment of community-based systems of emergency transport for sick children<br>- Establishment of community funds to pay for transport of sick children
Provision of adequate care for neonates in health facility	- Training of facility-based health workers in the management of sick neonates<br>- Improved communication between health workers and parents of sick children

Activities to improve the of care for sick neonates may include organization of a community-based system of emergency transport for sick infants, education of parents and community health workers, and institution of a system of referral slips (Kalter et al. 2001; Macintyre and Hotchkiss. 1999; Nordberg et al. 1996). Recent research in Imbabura, Ecuador conducted as part of a WHO multi-country study on referral suggests that using referral slips, rather than simply making a verbal referral makes the caretakers significantly more likely to comply with referral (Kalter et al. 2001).

Summary

We propose to conduct community-based, prospective surveillance aimed at determining the organisms causing serious infections in neonates. Concurrently, we will implement an intervention aimed at reducing neonatal mortality through provision of essential obstetric and newborn care; identification of neonatal sepsis in the community; referral and community-based transport of sick neonates and strengthening of neonatal care in health facilities. These studies will provide a foundation for future programmatic implementation of appropriate management strategies for prevention and treatment of infections in neonates in the community.

By a transfer of technology, this project also will equip Bangladeshi scientists to conduct epidemiological and microbiological investigations of other infectious disease problems in the community. The objectives of this study are strongly supported by The Minister of Health, Bangladesh (see attached letter).

Informed consent and methods utilized in surveillance and management strategies for patients in the intervention trial will be subject to the review and approval of the appropriate ethical review boards of the ICDDR,B; Shishu Hospital; The Johns Hopkins University (JHU); and Oxford University.

4. Research Design and Methods:

Describe in detail the methods and procedures that will be used to accomplish the objectives and specific aims of the project. Discuss the alternative methods that are available and justify the use of the method proposed in the study. Justify the scientific validity of the methodological approach (biomedical, social, or environmental) as an investigation tool to achieve the specific aims. Discuss the limitations and difficulties of the proposed procedures and sufficiently justify the use of them. Discuss the ethical issues related to biomedical and social research for employing special procedures, such as invasive procedures in sick children, use of isotopes or any other hazardous materials, or social questionnaires relating to individual privacy. Point out safety procedures to be observed for protection of individuals during any situations or materials that may be injurious to human health. The methodology section should be sufficiently descriptive to allow the reviewers to make valid and unambiguous assessment of the project. **(Do not exceed ten pages, use continuation sheets).**

Overview of study methods

Community-based surveillance to prospectively identify infections in neonates will be set up in a representative rural sub-district in Bangladesh. Every attempt will be made to obtain blood cultures on a high percentage of neonates presenting with signs/symptoms of sepsis. The primary analysis will include the species of organisms isolated from the blood and their antibiotic sensitivity patterns.

A randomized trial will be conducted to evaluate the impact of introducing a package of essential obstetric and neonatal care interventions, including identification of the barriers to care-seeking, design of strategies to address those barriers and management of serious bacterial infections, on neonatal mortality. The primary outcome measure will be changes in neonatal mortality rates in the intervention and control communities over the course of the trial.

Study Site and Population

The study will be conducted in Mirzapur, a rural sub-district (thana) of Bangladesh located 60 km north of Dhaka, the capital city. It has an estimated total population of 400,000, distributed in 13 unions and 219 villages. The birth and infant and child mortality rates (26/1000 population, 70/1000 live births and 100/1000 live births, respectively) of Mirzapur are very similar to the national averages. The estimated annual birth cohort in the sub-district is 10,400, or, on average, approximately 50 births per village per year, more than 95% of which occur at home. The nationwide neonatal mortality rate was 42/1000 according to the 1999 to 2000 Bangladesh Demographic and Health Survey. The population of Mirzapur is served by a 750-bed non-profit private hospital, Kumudini Hospital, with laboratory and radiology facilities. Children with severe illness requiring in-patent care are referred to this facility, or the 31-bed governmental Thana Health Complex. ICDDR,B has had a collaborative agreement with Kumudini Hospital since 1982, and a recent ICDDR,B study by co-applicant Dr. Hasan successfully utilized this hospital as a referral center and field laboratory (Hasan, 2000). Each union has a Health and Family Welfare Centre (H&FWC) staffed by paramedics. The H&FWCs are outpatient facilities that provide preventive and limited curative child and reproductive health services, including immunizations, antenatal

and postnatal care, to a population of approximately 30,000. Services at the community level are provided by family welfare assistants (FWA) and health assistants (HA); one FWA and one HA attend to a population of about 6,000 to 7,000. Despite this infrastructure, like most of Bangladesh, almost all births and deaths in Mirzapur occur in-home, and most commonly are attended by a TBA. Therefore, little cause-specific morbidity and mortality data is available, although informal discussions by the investigators of this study with local TBAs revealed that early infant deaths are largely associated with jaundice, fever, convulsions and unconsciousness, suggesting that many deaths may be due to septicemia, meningitis, or pneumonia.

Baseline Studies

Baseline census, mapping and household survey

A baseline census of the study areas will be conducted by enumerators to map and characterize the population. At the same time as the census, additional information will be collected in a household survey which will identify expectant mothers, and estimate the birth, infant death and neonatal death rates. The survey will be used to obtain retrospective pregnancy, birth and death information in the previous 2 years using a Demographic Health Survey-type instrument. Information on socio-economic and demographic variables, water and sanitation conditions and other possible confounders or correlates of mortality also will be collected in the baseline module so that any group differences can be adjusted for during data analysis.

Study cluster and randomization

Within each of the 13 unions in the study area, FWA areas will be assigned randomly to the intervention or control group to achieve geographic balance of villages over the entire Mirzapur thana. A computer-generated, restricted randomization scheme will be employed to achieve near balance on cluster size/birth cohort size and mortality rate within each union. Criteria for balance between the two sets of clusters in a union will include a baseline neonatal mortality rate within 30% of each other, and a birth rate within 20%. In addition, there will be restrictions to achieve overall balance within 10% for each of these measures.

Baseline qualitative studies

Baseline qualitative research will focus on the first two delays in the “three delay” model cited in the Background and Rationale. Key questions that the qualitative research will address are:

Table 2: Baseline Studies to Evaluate Causes of Delays in Seeking Neonatal Care

Where does delay occur	Corresponding research questions	Methods to be used to address research questions
Decision in the home to seek appropriate medical help	- What signs or symptoms in neonates prompt parents to seek outside care?<br>- What local terms are used to describe and categorize sick neonates?<br>- What are different sources of care for neonates?<br>- What are perceived advantages and disadvantages of different sources of care for neonates?<br>- What are the expectations regarding facility-based care?	- 50-60 semi structured interviews with women who recently delivered<br>- 10 semi structured interviews with TBAs
	- How is the decision made at the household level to seek care for a sick neonate? Who is responsible for making the decision, and what factors are taken into account when the decision is made?	- 20 semi structured interviews with parents of a sick neonate that has been brought to a health facility
Transport from home to facility providing appropriate care for neonates	- Who takes a sick neonate to a health facility, if mother cannot leave home or is not supposed to leave home?<br>- What means of transport are commonly used to take sick children from the home to a health facility?<br>- What are the perceived advantages and disadvantages and costs of different means of emergency transport?<br>- What sources of funds exist for families to pay for emergency transport and care at health facilities for sick neonates?	- 6 focus group discussions with mothers of young children<br>- 3 focus group discussions with fathers of young children

Results of the baseline qualitative studies will be used to design health care messages using local terms, to communicate with the communities, and to assess the acceptability of various forms of education and intervention.

Sample size calculations

Sample size for identification of agents of neonatal infection

We projected that the incidence of serious bacterial infection in our study neonates will be 1.4%, based on the report of Stoll that 20% of neonates in developing countries develop infection (Stoll. 1997), and data from the WHO young infant study demonstrating that 7% (167/2398) of blood cultures obtained from infants less than 90 days of age with clinically suspected sepsis were positive (WHO. 1999) (i.e., 0.2 x 0.07 = 0.014). If the sepsis rate is 1.4% with 40% precision (i.e., the lowest acceptable rate is 0.8%), and if we had a simple random sample, we would needed 1,284 newborns to estimate this rate with a 95% significance level. Because this is a community-based study with clusters as the unit of randomization, it is reasonable to assume that the samples are more homogenous within than between the clusters. Thus, a higher design effect for the variance estimations is required. To compensate for the underestimated variances, we will inflate the sample size 2-fold to 2,568 newborns (or 514 clinically suspected sepsis cases assuming that 20% will have clinically suspected sepsis). A design effect of 2.0 is very reasonable; with a cluster size equal to 150 to 200 births per cluster, this indicates that the intra-cluster correlation is 0.02. Therefore, we will attempt to collect culture specimens from 514 suspected sepsis cases. We should be able to achieve this sample size as we plan to enroll 4044 newborns in the intervention clusters

(see sample size calculations for intervention); the expected number of clinical sepsis cases in 4044 newborns should be about 809.

Sample size for evaluation of intervention

Assuming a baseline neonatal mortality rate of 40/1000, an individually randomized study with 2022 newborns in each group would be sufficient to detect a mortality reduction of 40% in the intervention arm. As this is a community-randomized trial, doubling the sample size should be sufficient to account for between-community variability. Therefore, 4044 newborns need to be enrolled and followed in each study arm. The annual birth cohort of 10,400 in the study area will be more than sufficient to meet this target, even considering that the 5% of neonates who are born in a health care facility will not be included. After the first few months of the trial, an interim analysis of the design effect will be performed as a check on the doubling factor. This analysis will not affect the Type I error, but may lead to adjustments in the length or size of the trial (most likely in the direction of shortening it).

Sample size for Community-based transport

Sample size calculations for community based transport indicators are shown in Table 4. The indicator requiring the highest number of subjects is for patients deteriorating during transport (n=876). Even though not all patients will require transport, there should be enough subjects to achieve this number.

Table 4: Sample Size Calculations for Neonatal Mortality and Community Transport System

**Measure or comparison**	**Expected**	**Desired precision/ difference**	**Sample size estimate**
Difference in neonatal mortality in intervention and control arms	24/1000 in intervention group	±40%	2,202x2[@]=4,044 in each group
Percent of neonates referred to the transport system that make it to Kumudini Hospital	80%	±10%	189x10[*]=1,890
Percent of children who deteriorate during transport	15%	±10%	438x10[*]=4,380
Average duration of transport	5 hours	± 1 hour<br>Assumed SD=3	95x10[*]=950
Percent of time that community transport is available	80%	±10%	189x6.7[*]=1,266
Percent of all referred neonates arriving at Kumudini Hospital who used the community transport	70%	±10%	233x6.7[*]=1561

[@] Adjustment for design effect (cluster design). [*] Adjustment to number of newborn birth required.

All calculations are based on a 95% confidence level. The sample size is doubled in each case to account for the design effect of clusters. In addition, because many of the indicators will only apply to those referred or those who were transported, additional adjustments were made. We estimate that 15% of neonates will be referred and 10% will be transported through community based transport so we have multiplied our sample by the inverse (1/0.15=; 1/0.1=10) to reflect the number of newborns required. The number of subjects required for community transport indicators is significantly less than that required for surveillance and mortality outcome measures so these estimates will have a higher level of precision than necessary.

Description of the intervention

All pregnant women and newborns in the comparison clusters will receive the usual obstetric and neonatal care available in the community. In the intervention clusters, a team of TBAs and CHWs will be trained to provide, and to educate mothers/caregivers to participate in providing a package of essential obstetric and neonatal care.

Community health education

Education activities will be conducted in the intervention communities using a variety of media to provide specific health messages on the importance of:

a. Prenatal care, including promotion of tetanus toxoid immunization, and adequate nutrition during pregnancy
b. Hygienic delivery practices
c. Identification of signs signaling the need for emergency obstetric care and indications for self-referral
d. Early recognition and prompt management of birth asphyxia
e. Early, exclusive breast feeding
f. Temperature maintenance
g. Hygiene for preventing infections
h. Monitoring of newborn weight gain
i. Recognition of danger signs and symptoms in neonates
j. Appropriate health-seeking behavior for sick neonates

Role of the TBA

Trained, skilled TBAs within intervention clusters will be given incentives to attend all births within their cluster and to report all births within 12 hours to the CHW for that cluster if the CHW fails to attend the delivery. TBAs in the intervention clusters will be instructed and evaluated in the performance of simple interventions to improve delivery and neonatal care. Safe delivery and neonatal care kits will be provided for all deliveries in the intervention area.

Interventions which TBAs will be trained to introduce will include:

a. Referral of complicated cases for emergency obstetric care to Kumudini Hospital.
b. Appropriate hygiene during delivery and the immediate post-partum period, including hand-washing, use of clean instruments and a clean delivery surface, minimization of vaginal examinations, avoidance of inserting foreign objects into the vagina, cord cutting with a clean blade and tying with clean thread, and clean cord care including application of antiseptics (e.g., gentian violet) to the umbilical stump.
c. Neonatal resuscitation, including recognition of birth asphyxia within one minute of birth, clearance of mucus with an oral mucus sucker/trap, provision of tactile stimulation and, when necessary, artificial breathing with mouth-to-mask or tube-and-mask techniques.
d. Thermal control, including drying the newborn with warm, clean towels immediately after birth, swaddling the newborn in layers of cloth and dressing the neonate as appropriate for the season, covering the newborn’s head, promoting skin-to-skin contact with the mother, and utilizing skin-to-skin contact and/or sleeping bags designed for re-warming hypothermic neonates.
e. Promotion of breastfeeding, including feeding of colostrum, avoidance of prelacteal feeds, and immediate, exclusive breast-feeding.

Role of the CHW

Trained CHWs will also attend all deliveries and assist the TBA as needed. The CHW will be primarily responsible for providing newborn care (i.e., items c, d, and e described

under the role of TBAs), but the TBA will also be trained in newborn care so that the TBA can work with the CHW as a team or provide this care in the absence of the CHW. The CHW will collect baseline data within 24 hours of birth using pre-tested questionnaires. Baseline data will include birth weight, household socioeconomic status, factors which impact risk for infections [e.g., crowding, sanitation, exposure to smoke, maternal tetanus immunization status, maternal nutritional status (e.g., mid upper arm circumference), premature/prolonged rupture of membranes, maternal fever, delivery care including management of birth asphyxia, thermal control, feeding practices, prophylaxis of ophthalmia neonatorum, umbilical cord care, bathing and skin care practices], and distance to a health facility. will follow the neonate regularly through the neonatal period as part of the surveillance activities (see the surveillance method below). The CHWs will continue to visit the study neonates in the intervention communities regularly in their homes and collect data on signs and symptoms of potential infections using a standardized questionnaire. Visits will occur on days 1, 3 and 7 and twice weekly thereafter for the first 28 days of life. No visits will be made to the homes of neonates in the control communities.

The CHWs will be trained and evaluated in the provision of essential newborn care, including:

a. Identification of high risk neonates who: a) weigh less than 2000 g within 24 hours of birth on a hand-held, spring balance accurate to within 10 g, or, b) are less than 37 weeks gestational age as determined from the date of last menstruation. These neonates will be followed more closely then full-term, appropriate-for-gestational-age neonates, with home visits every other day for the first 10 days of life, and twice weekly thereafter for the first 28 days of life.

b. Promotion of early, exclusive breastfeeding, including feeding of colostrum and avoidance of prelacteal feeds. CHWs will be trained to teach mothers how to feed expressed breast milk with a spoon to neonates who suck poorly. CHWs will also be trained in the recognition and management of common breast problems during lactation (e.g., inverted nipples, mastitis, breast abscess).

c. Temperature maintenance, including monitoring axillary temperature.

d. Appropriate bathing and skin care practices.

e. Clean umbilical cord care, and management of umbilical cord and superficial skin infections.

f. Identification and management of neonates with suspected serious bacterial infections.

Neonates who are suspected of having sepsis or other serious conditions by CHWs will be referred to Kumudini Hospital for appropriate care. CHWs will refer neonates needing help with travel to the community maintained transport system (see section on “community sustained emergency transport” below). If hospitalization of neonates with suspected sepsis is refused, the CHW will provide all possible and necessary management of the illness at home. These neonates will be excluded from “etiology” component of this study. Repeated attempts to refer the child will be made by the CHW during the follow-up visits.

Patients who comply with referral to Kumudini Hospital will be re-evaluated by trained nurses and doctors. If the patient is confirmed to meet high-risk criteria for sepsis, they will be appropriately cultured and treated with benzathine penicillin and gentamicin according to WHO guidelines (WHO. 1989; WHO. 1990). Treatment of neonates who are culture-positive for one or more pathogens will continue for 10 to 14 days. Hospitalized neonates will be kept in the hospital until clinically stable (i.e., normal feeding, stable body temperature); thereafter, consideration will be given to continuing treatment at home with an oral antibiotic (cefprozil: 20mg/kg/day divided twice daily). If the condition of the baby

deteriorates during the home treatment period, the parents again will be encouraged to take the child again to Kumudini Hospital. Antibiotic sensitivity testing performed at Dhaka Shishu Hospital Microbiology Laboratory on all isolates will be used to tailor antibiotic therapy when appropriate, particularly for management of gram-negative pathogens. Patients who leave Kumudini Hospital against medical advice will also be given oral cefprozil and followed daily at home by the CHW as for those who initially refused referral. Culture-negative infants will be managed as clinically indicated.

Dr. Saha and colleagues are currently conducting a study to determine the identity and antibiotic susceptibility profiles of the bacterial organisms that colonize the vagina of women in Mirzapur and from surrounding areas in Bangladesh. We may alter the choice of antibiotics for the treatment of neonatal sepsis based on the findings of this study. Bang et al. successfully used this approach for determining the antibiotic treatment for neonatal sepsis in their study (Bang et al. 1993).

Role of the mother/caregivers

The mother/caregivers will be trained and monitored by the TBA and CHW in essential newborn care in the home including feeding practices, temperature maintenance, appropriate hygiene, umbilical cord care, bathing and skin care practices, and recognition of danger signs of potentially serious illness. The mother/caregivers will be instructed in indications for self-referral and the need to contact the CHW on days when the CHW is not scheduled to visit the home.

Community sustained emergency transport

Planning sessions will be held with community leaders to identify locally sustainable emergency transport mechanisms. CHWs will keep track of patients referred via these mechanism and will document a physical examination within an hour before departure. They will periodically summarize emergency transport instances looking at average duration of transport and complications of transport. Community leaders will be encouraged to take appropriate actions to improve the transport mechanisms when problems are identified. Locally sustainable emergency transport systems have been used successfully in Kenya, and Nigeria, and are being implemented in Matlab, Bangladesh (Macintyre and Hotchkiss. 1999; Shehu et al. 1997; Iqbal. 2001). While we will use some of these strategies, the plan for Mirzapur will need to be adapted to meet local circumstances. Process indicators (see Table 4) will be analyzed periodically for all communities to monitor the effectiveness of the system overall. While we will focus on transport of neonatal and obstetric emergencies, the transport system will extend to all emergencies in the community.

Method to identify aetiology of newborn infections

As noted earlier, trained, skilled CHWs will be asked to attend all deliveries in her assigned intervention cluster. The trained, skilled CHWs will visit newborns in the home within 24 hours of delivery, and will continue to visit the study neonates in the intervention communities regularly in their homes and collect data on signs and symptoms of potential infections using a standardized questionnaire (see above, Role of CHW). Neonates identified by the CHW as having two or more of the following signs will meet criteria for having a suspected serious bacterial infection (i.e., sepsis, meningitis, and/or pneumonia): ill appearance; agitation; extreme lethargy/decreased spontaneous movement/poor neuromuscular tone (i.e, “loose limbs”, “floppy”; decreased, abnormal, or absent cry; poor feeding (e.g., weak or reduced suck); high (>38°C) or low (<36°C) temperature in the absence of an identifiable breach in thermal control; respiratory rate >60/min, chest in-drawing, or grunting; umbilical cord infection (i.e., erythema, tenderness, and/or discharge

around the umbilicus); seizure(s); or delayed digital capillary refill. Neonates with clinically suspected serious bacterial infection will be referred and if needed, accompanied by the CHW to Kumudini Hospital where cultures of blood, cerebrospinal fluid (CSF) and urine will be obtained by trained doctors. We will culture from the umbilical cord in neonates with signs of umbilical cord infection. Blood will be collected in tubes containing sodium polythenol sulphonate and saponin, and cultured using the lysis-centrifugation technique shown by our group at Shishu Hospital to be cost-effective and highly sensitive compared to conventional methods (Saha et al. 1992). Urine and CSF cultures will be performed by standard microbiological techniques. A 250 to 500 *u*l aliquot of blood or CSF also will be placed in a sterile tube with EDTA and frozen for future studies, including molecular diagnostics. If referral to Kumudini Hospital is refused, the CHW will attempt accompanied referral to the nearest H & FWC where a trained doctor will obtain blood cultures. Study doctors will make frequent field visits to supervise and support the work of the nurses and to ensure the quality of specimens collected. Specimens will be transported daily from the H & FWC in cool boxes to Kumudini Hospital where initial plating will be performed as described previously (Saha et al. 1992; Saha et al, 1997). Plates will be transported daily from Kumudini Hospital to Shishu Hospital for further organism identification, antibiotic susceptibility testing, and molecular diagnostic testing. Quality assurance of the microbiological procedures will be maintained through regular verification of organism identification using standard American Type Culture Collection strains and checks of compliance with standard zones of inhibition and minimum inhibitory concentrations per standards of the National Committee for Clinical Laboratory Standards. Serious bacterial infection is defined as recovery of at least one pathogen in blood, CSF and/or urine cultures obtained in the presence of compatible clinical signs of systemic illness. A pathogen is defined as a microorganism known to cause infection in neonates.

Outcome variables

The primary outcome, changes in neonatal mortality rates in the intervention and control communities from the beginning to the end of the study period, will be based on accurate measurement of mortality rates. Trained field workers (FWs) will be responsible only for a) counting pregnancies, live births (i.e., birth of a live infant after 28 weeks of gestation), neonatal and infant deaths, and b) conducting verbal autopsies in reported neonatal deaths. They will have no training in newborn care. The FWs will collect demographic data from both the intervention and control communities on a 3-monthly basis. Presumably, the CHWs will provide a record of every newborn in the intervention clusters, but we may not know about all births and deaths in the comparison clusters. To minimize this potential bias, the FWs will ascertain and record the outcome of all reported pregnancies (stillborn, live-born, miscarriage) within the control and intervention communities. We will compare the records of the CHWs to those of the FWs on births and deaths in the intervention clusters on an ongoing basis to estimate whether these events are undercounted by the FWs. If found, we will attempt to determine the reasons for such undercounting and will use this information to retrain the FWs.

A secondary outcome measure, cause-specific neonatal mortality rates, will be based on information collected by trained health care workers on a 3-monthly basis using verbal autopsies to determine the cause of all neonatal deaths in both the intervention and control villages. These workers, however, will not have any training on neonatal care, and their work will be independent of the intervention. Verbal autopsies will be conducted using an instrument recently validated by our group for use in Bangladeshi neonates (Kalter et al. 1999), and cause of death will be determined independently by co-applicant pediatricians at Shishu Hospital who will review the verbal autopsy records. We are currently involved in further developing and adapting a verbal autopsy method for neonates in Bangladesh. Death

will be categorized as due to prematurity, sepsis/meningitis/pneumonia, diarrhea, asphyxia, tetanus, hypothermia, birth trauma, or congenital anomalies, others and not known.

We will use the following outcome indicators to evaluate the effectiveness of the community transport system.

1. Percent of neonates referred to the transport system that make it to Kumudini Hospital
2. Percent of children who deteriorate during transport
3. Average duration of transport
4. Percent of time that community transport is available.
5. Percent of all referred neonates arriving at Kumudini Hospital who used the community transport.

These outcome measures will be determined based on information the CHW will collect prior to transport and information that will be recorded on arrival at Kumudini Hospital.

Data quality and management

The senior study physician at Kumudini Hospital will have overall responsibility for assuring the quality of the work of the other study physicians. The Study Coordinator will have overall responsibility including ensuring quality of the works done by the enumerators, TBAs, CHWs, and FWs. All questionnaires and data forms will be reviewed for accuracy, consistency and completeness. To ensure data quality, the Study Coordinator, senior study physician, and investigators will make periodic filed visits to observe data collection by the enumerators, CHWs and FWs. In addition, a 5% sample of neonates in the intervention trial will be re-evaluated within 2 days of the original interview/measurement. After editing, the data will be entered in databases using on-line custom-designed data entry programs. Necessary range and consistency checks will be in-built. Data will be periodically checked by running and reviewing frequency distributions and cross-tabulations.

Data Analysis

Baseline characteristics of the intervention and control groups will be examined for group comparability. Any significant baseline differences will be controlled for during data analysis. The primary analysis will involve estimation of neonatal mortality rates, percent reduction in neonatal mortality rates in the intervention and control areas and the difference in neonatal mortality between the two areas. Appropriate and standard bivariate and multivariate statistical techniques will be used. To account for the clustered nature of the data, techniques such as Generalized Estimating Equation and multi-level modeling will be used.

Data Safety and Monitoring Committee:

A three-member **data safety and monitoring committee (DSMC)** will be established. The committee will have members from both within ICDDR,B and outside and will include a statistician, a clinician, and one person experienced in field trials. The committee will review results of interim data analyses.

5. Contribution of the Partners and Training Activities:

To build capacity within Bangladesh by training Bangladeshi scientists in epidemiological and microbiological techniques and clinical practice through our on-going collaboration with Shishu Hospital and the ICDDR,B.

Well-established and newly formed collaborative alliances among the scientists with proven track records at the participating institutions create a synergy of effort which will enable us to address infections in neonates in a practical yet highly technical manner not

possible at any one of the institutions alone. Moreover, the existing infrastructure in Bangladesh provides the necessary foundation for the successful transfer of technology from the USA- and UK-based institutions to Bangladesh.

Resources at the USA and UK institutions will be focused on further building the capacities of Bangladeshi scientists and the government-associated institutions Shishu Hospital and the ICDDR,B. Johns Hopkins University (JHU) will serve as a training site for Bangladeshi scientists in epidemiology and clinical practice in the care of neonates. The epidemiological investigations will be led by Dr. Arifeen (who obtained his doctoral degree at JHU) of the ICDDR,B with support from the JHU group. Three Bangladeshi scientists will attend a summer course in fundamentals of epidemiology/data management, data analysis at JHU. One of these individuals will be the lead study pediatrician based at Kumudini Hospital. This individual also will receive training for 1 month in neonatal clinical assessment and care at JHU. In addition, a JHU neonatologist will conduct a 10-day training course on-site in Mirzapur. The course will provide instruction in neonatal care appropriately tailored for the study physicians, nurses, TBAs and CHWs. These activities will build on long-standing collaborations between the JHU and Bangladeshi co-applicants over the past 20 years.

Microbiological testing will be conducted under the direction of Dr. Saha at Shishu Hospital, with technical assistance in Bangladesh from the Oxford group. The Oxford-based Centre for Vaccine and Tropical Medicine Research with the Department of Microbiology will support establishment of the diagnostic laboratory infrastructure. Initial emphasis will be placed on optimizing the Kumudini Hospital and Shishu Hospital laboratories where routine culture techniques will be undertaken. The Oxford group also will provide ongoing quality assurance and validation of test results obtained in the Shishu Hospital and field laboratories. The Oxford-based investigators have experience setting up field laboratories for Wellcome Trust-funded Tropical Units in Kenya and South East Asia.

Capacity building in Bangladesh also will involve establishing molecular techniques at Shishu Hospital for detecting micro-organisms based on DNA amplification and sequence typing of organisms. Dr. Saha will be trained in these techniques in Oxford under the direction of the Oxford co-applicants. He also will receive on-site supervision from the Oxford group in establishing these capabilities at Shishu Hospital. These molecular techniques offer opportunities for conducting non-culture based detection of bacterial meningitis or bacteremia and will be a valuable technology for building the research infrastructure in Bangladesh. This will underpin future detailed research studies of infection. Bacterial isolates from the community-based surveillance activities (AIM 1) will provide a rich source of material for use in establishing the techniques. Moreover, optimization of conventional microbiological techniques will enable the conventional culture results to be used as a gold standard for assessing the sensitivity of the molecular diagnostic techniques. We propose to optimize the polymerase chain reaction (PCR) detection of bacteria from CSF in cases of meningitis using the approach of Radstrom *et al.* (Radstom et al. 1994) supplemented by the techniques developed for detecting bacteria in blood described below. Organisms for which multi-locus sequence typing (MLST) schemes exist will be directly typed from CSF as described previously by the Oxford group (Enright et al. 2000). These techniques will be validated against the culture results and MLST of organisms cultured from CSF. Techniques for detecting bacterial pathogens in blood or CSF will be developed based on PCR amplification of a generic 16S ribosomal target (Geisen et al. 1994). This technique will be adapted to determine the genus and/or species of the organism by sequence analysis of the PCR amplicons. As there have been problems with the specificity of PCR of blood samples arising from its exquisite sensitivity, semi-quantitation of target sequences will be developed based on invasive cleavage of oligonucleotide probes (Lyamichev et al. 1999). This will also act as a confirmatory test of the PCR. The availability of culture results will

enable validation of these amplification techniques in preparation for undertaking future molecular diagnostic investigations at Shishu Hospital.

Timetable

Months 1 to 9: Baseline community census, mapping and household survey; ethnographic studies and formative qualitative research; design and pretesting of instruments; training of enumerators, study physicians, TBAs, CHWs, nurses, paramedics, and FWs; preparation of clinical facilities at Kumudini hospital and the H & FWCs; and preparation of field laboratories. Community meetings to prepare community transport system.

Months 10 to 24: Patient enrollment and conduct of surveillance and intervention activities.

Months 25 to 36: Final demographic surveys, completion of laboratory analyses, data analyses and report preparation. If the intervention package is shown to be efficacious, training of TBAs and CHWs in the control villages or workers in a local non-governmental organization, and identification of resources for sustaining the interventions, will be undertaken in the final year.

References

World Health Organization: Perinatal mortality: A listing of available information. Geneva, Switzerland, WHO, 1996.

Bartlett AV, Bocaletti MEPD, Bocaletti MA. Neonatal and early postneonatal morbidity and mortality in a rural Guatemalan community: the importance of infectious diseases and their management. Pediatr Infect Dis J 1991;10:752-7.

Stoll BJ. The global impact of neonatal infection. Clin Perinatol 1997;24:1-21.

Arifeen SE. Birth weight, intrauterine growth retardation, and prematurity: a prospective study of infant growth and survival in the slums of Dhaka, Bangladesh. Doctor of Public Health Dissertation, Johns Hopkins University, 1997.

Baqui AH, Black RE, Arifeen SE, Hill K, Mitra SN, Sabir AA. Causes of childhood deaths in Bangladesh: results of a nationwide verbal autopsy study. Bull World Health Organ 1998;76:161-71.

Antelman G. Determinants of perinatal mortality, preterm delivery and intrauterine growth retardation among the urban poor in Dhaka, Bangladesh. Doctor of Science Dissertation, Johns Hopkins University, 1997.

Mitra SN, Ahmed AS, Cross AR, Jamil K. Bangladesh demographic and health survey, 1996-1997. Dhaka and Calverton, Maryland: National Institute of Population and Training (NIPORT), Mitra and Associates, and Macro International, Inc.

Islam MS, Rahaman MM, Aziz KMS, Rahman M, Munshi MH, Patwari Y. Infant mortality in rural Bangladesh: an analysis of causes during neonatal and postneonatal periods. J Trop Pediatr 1982;28:294-8.

World Health Organization. Safe Motherhood. Mother-baby Package: implementing safe motherhood in countries. Maternal Health and Safe Motherhood Programme. Geneva, Switzerland, WHO, 1994, FHE/MSM/94.11.

Bang AT, Bang RA, Baitule SB, Reddy MH, Deshmukh MD. Effect of home-based neonatal care and management of sepsis on neonatal mortality: field trial in rural India. Lancet 1999;354:1955-61.

Ahmed S, Sobhan F, Islam A, e-Khuda B. Neonatal morbidity and care-seeking behaviour in rural Bangladesh. J Trop Pediatr 2001;47:98-105.

Zoysa ID, Bhandari N, Akhtari N, Bhan MK. Careseeking for illness in young infants in an urban slum in India. Soc Sci Med 1998;47:2101-2111.

WHO Young Infants Study Group. Bacterial etiology of serious infections in young infants in developing countries: results of a multicenter study. Pediatr Infect Dis J 1999;18:S17-22.

Darmstadt GL, Black RE, Santosham M. Research priorities and postpartum care strategies for the prevention and optimal management of neonatal infections in less developed countries. Pediatr Infect Dis J 2000;19:739-50.

Mitra SN, Sabir AA, Cross AR, Jamil K. Bangladesh Demographic and Health Survey, 1996-97. Dhaka and Calverton, Maryland: National Institute of Population Research and Training (NIPORT), Mitra and Associates, and Macro International Inc, 1997.

Bang AT, Bang RA, Morankar VP, Sontakke PG, Slanki JM. Pneumonia in neonates: can it be managed in the community? Arch Dis Child 1993;68:550-6.

Barnes-Josiah D, Myntti C & Augustin A (1998) The "three delays" as a framework for examining maternal mortality in Haiti. Soc Sci Med 46, 981-93.

Kalter HD, Moulton L, Black RE, Salgado R, Contreras A, Nieto P & Egas ML (2001) Referral constraining factors: Imbabura Province, Ecuador Paper presented at meeting "Research for community implementation of WHO/UNICEF's Integrated Management of Childhood Illness (IMCI) ", Baltimore MD, January 22-24, 2001. Johns Hopkins University, Baltimore MD

Macintyre K & Hotchkiss DR (1999) Referral revisited: community financing schemes and emergency transport in rural Africa. Soc Sci Med 49, 1473-87.

Nordberg E, Holmberg S & Kiugu S (1996) Exploring the interface between first and second level of care: referrals in rural Africa. Trop Med Int Health 1, 107-11.

World Health Organization. Programme of acute respiratory infections. Report of the fourth meeting of technical advisory group 6-10 March 1989. Geneva, Switzerland, WHO, 1989, WHO/ARI/89.4.

World Health Organization. Acute respiratory infections in children: case management in hospitals in developing countries. Geneva: WHO, 1990. (WHO/ARI/90.5.)

Hasan KZ. Risk factors associated with acute lower respiratory tract infection in a cohort of newborns from birth to 24 months of age in a rural community of Bangladesh. Doctor of Public Health Dissertation, University of Alabama at Birmingham, 2000.

Saha SK, Khan WA, Saha S. Blood cultures from Bangladeshi children with septicaemia: an evaluation of conventional, lysis direct plating and lysis centrifugation methods. Trans Royal Soc Trop Med Hyg 1992;86:554-6.

Saha SK, Rikitomi N, Ruhulamin M, Watanabe K, Ahmed K, Biswas D, Hanif M, Khan WA, Islam M, Matsumoto K, Nagatake T. The increasing burden of disease in Bangladeshi children due to *Haemophilus influenzae* type b. Ann Trop Paediatr 1997;17:5-8.

Personal communication with Dr. Samir Saha, July-2001. Dhaka Shishu Hospital. Based on price at Dhaka Shishu Hospital General Pharmacy.

Kalter HD, Hossain M, Burnham G, Khan NZ, Saha SK, Ali MA, Black RE. Validation of caregiver interviews to diagnose common causes of severe neonatal illness. Paediatr Perinat Epidemiol 1999;13:99-113.

Radstrom P, Backman A, Qian N, Kragsbjerg P, Pahlson C, Olcen P. Detection of bacterial DNA in cerebrospinal fluid by an assay for simultaneous detection of *Neisseria meningitides*, *Haemophilus influenzae*, and streptococci using a seminested PCR strategy. J Clin Micro 1994;32:2738-44.

Enright M, Knox K, Griffiths D, Crook D, Spratt B. Molecular typing of bacteria directly from cerebrospinal fluid. Eur J Clin Microbiol Infect Dis 2000;19:in press.

Greisen K, Loeffelholz M, Purohit A, Leong D. PCR primers and probes for the 16S rRNA gene of most species of pathogenic bacteria, including bacteria found in cerebrospinal fluid. J Clin Microbiol 1994;32:335-51.

Lyamichev V, Mast A, Hall J, Prudent J, Kaiser M, Takova T, Kwiatkowski R, Sander T, de Arruda M, Arco D, Neri B, Brow M. Polymorphism identification and quantitative detection of genomic DNA by invasive cleavage of oligonucleotide probes. Nat Biotechnol 1999;17:292-6.

Macintyre K, Hotchkiss DR. Referral revisited: community financing schemes and emergency transport in rural Africa. Soc Sci Med 1999 Dec;49(11):1473-87.

Shehu D, Ikeh AT, Kuna MJ. Mobilizing transport for obstetric emergencies in northwestern Nigeria. The Sokoto PMM Team. Int J Gynaecol Obstet 1997 Nov;59 Suppl 2:S173-80.

Shenep JL, Flynn PM, Baker DK, Hetherington SV, Hudson MM, Hughes WT, Patrick CC, Roberson PK, Sandlund JT, Santana VM, Sixbey JW, Slobod KS. Oral cefixime is similar to continued intravenous antibiotics in the empirical treatment of febrile neutropenic children with cancer. Clin Infect Dis 2001 Jan;32(1):36-43.

Hoberman A, Wald ER, Hickey RW, Baskin M, Charron M, Majd M, Kearney DH, Reynolds EA, Ruley J, Janosky JE.Oral versus initial intravenous therapy for urinary tract infections in young febrile children. Pediatrics 1999 Jul;104(1 Pt 1):79-86.

Al-Eidan FA, McElnay JC, Scott MG, Kearney MP, Troughton KE, Jenkins J. Sequential antimicrobial therapy: treatment of severe lower respiratory tract infections in children. J Antimicrob Chemother 1999 Nov;44(5):709-15.

Klaassen RJ, Allen U, Doyle JJ. Randomized placebo-controlled trial of oral antibiotics in pediatric oncology patients at low-risk with fever and neutropenia. J Pediatr Hematol Oncol 2000 Sep-Oct;22(5):405-11.

Personal Communication. Iqbal, Anwar. Community Ambulance Initiative Under EOC Matlab Project, International Centre for Diarrhoeal Diseases Research, Bangladesh (ICDDR,B), 2001.

6. Appendix: Use of Oral Antibiotics in the Management of Neonatal Sepsis

Standard of care

Recommended treatment of serious systemic bacterial infections in neonates (i.e., septicemia, pneumonia and meningitis) in developed countries includes parenteral administration of antibiotics in a health care facility. Similarly, the WHO recommends parenteral antibiotic therapy (e.g., benzylpenicillin or ampicillin plus an aminoglycoside such as gentamicin) in a health facility for treatment of serious neonatal infections in developing countries (WHO, 1989; WHO, 1990). In resource poor countries, however, the majority of births and neonatal deaths take place in the home, and families often are reluctant to seek care outside the home for neonatal illness for a variety of reasons, including cultural (e.g., confinement after birth), economic, logistical (e.g., lack of transportation), and health care availability (e.g., lack of quality referral-level care) factors (Bang, 1999; Datta, 1987; Zoysa, 1998; Ahmed, 2001).

Home-based treatment of neonatal sepsis with injectable antibiotics

Little information is available on treatment of serious neonatal infections in the community in developing countries. The most recent data available is from a study of domiciliary newborn care in India, cited above (Bang, 1999), which demonstrated that trained and supervised community health workers were capable of identifying and successfully treating neonatal infections in the home using a combination of oral and injectable antibiotics. Introduction of a package of interventions that included administration of oral cotrimoxazole (10 mg trimethoprim twice daily for 7 days) and intramuscular gentamicin (5 mg twice daily for 10 days in preterm infants and 7.5 mg twice daily for 7 days in term infants) for treatment of suspected “sepsis” (including septicemia, pneumonia, and meningitis) resulted in a 76% reduction in the cause-specific neonatal mortality rate attributed to sepsis, and this decrease in deaths among neonates with signs of sepsis accounted for three-fourths of the 59% total reduction in neonatal mortality. Case fatality attributed to sepsis was reduced in the intervention area from 16.6% before to 2.8% after introduction of the home-based package of neonatal care. Of note, neonatal case fatality was 13% in the same area in a previous study when village health workers focused on recognition of pneumonia and treated suspected cases with oral co-trimoxazole (Bang, 1994). The precise contribution that the addition of injectable gentamicin made to mortality reduction above that due to oral cotrimoxazole alone cannot be determined, however, as many other aspects of neonatal care were introduced in the more recent trial of home-based neonatal care, including health education of pregnant women regarding care during pregnancy, breastfeeding counseling, and recognition and care-seeking for neonatal danger signs; clean delivery practices; prevention and management of birth asphyxia, neonatal skin and umbilical infections, hypothermia and breastfeeding problems; careful surveillance for problems in preterm and low birth weight infants; and recognition and management of a broader range of serious bacterial infections, including sepsis and meningitis in addition to pneumonia. Nevertheless, antibiotic susceptibility testing of vaginal isolates from women in the community showed that 94% of isolates were susceptible to cotrimoxazole alone; all isolates were susceptible to the combination of cotrimoxazole and gentamicin (Bang, 1992). Since the agents of neonatal sepsis and pneumonia, particularly early-onset infections during the first week of life come in large part from the birth canal, these data suggest that on the basis of antibiotic susceptibility patterns in isolation, oral cotrimoxazole alone would have been expected to provide adequate coverage against the majority of neonatal bacterial pathogens, and the incremental benefits of adding

gentamicin might have been relatively small. This is an over simplification, however, as in practice, there are many other factors that play a role in effectiveness of the therapy.

In an uncontrolled study in India of domiciliary management by village health workers of high-risk neonates, including preterm and low birth weight infants, those with feeding problems, illness or a history of prolonged and difficult labor, in the absence of management of sepsis, neonatal mortality declined by 25% (Pratinidhi, 1986). Bang et al. (1999) suggested that the higher reduction in neonatal mortality in the SEARCH neonatal care study (59%) may have been due to management of sepsis, although this is speculative.

Other community-based studies in Guatemala (Bartlett, 1990; reviewed above) and in urban slums in New Delhi, India (Bhandari, 1996), have reported low neonatal case fatality (0% and 3%, respectively) with use of injectable antibiotics to treat neonates with suspected sepsis. In the Indian study, oral cephalexin was given in combination with injectable amikacin (Bhandari, 1996).

Home-based treatment of neonatal pneumonia with oral antibiotics

Data on management of neonates with serious bacterial infections using oral antibiotics in the home in developing countries comes almost exclusively from trials of pneumonia case management. Our group (Sazawal, 1992; revised analysis unpublished) has conducted a meta-analysis of all published and unpublished community-based intervention trials on case management of pneumonia in unselected preschool-aged children in developing countries. The impact of pneumonia case management on total neonatal mortality and pneumonia-specific neonatal mortality was determined. Of the six studies that compared neonatal mortality in concurrent control and treatment groups (Kielman, 1978; Mtango, 1986; Khan, 1990a; Khan, 1990b; Bang, 1990; Pandey, 1991; Fauveau, 1992), one lacked data on pneumonia-specific mortality (Khan, 1990). In four of the studies, neonates with suspected pneumonia were treated with oral cotrimoxazole (Mtango, 1986; Khan, 1990a; Khan, 1990b; Bang, 1990; Pandey, 1991); injectable penicillin was used in one study (Kielman, 1978), and another used both injectable penicillin and oral ampicillin (Fauveau, 1992). Uncorrected analysis showed a 20% to 30% reduction in all-cause neonatal mortality (odds ratio 0.72, 95% CI 0.62-0.83). After correction for perceived biases that may have affected the study results, the estimated reduction in total and pneumonia-specific neonatal mortality was 30% (95% CI 15% to 42%) (OR 0.70, 0.58-0.85) and 47% (95% CI 20% to 64%) (OR 0.53, 0.36-0.80), respectively. Similar results were found when additional studies were evaluated which had a before-after design. Data from three studies showed a 14% (95% CI 7% to 31%) reduction in total neonatal mortality (Mtango, 1986; Khan, 1990a; Khan, 1990b; Fauveau, 1992), and two studies combined to show a 36% (95% CI 16% to 65%) reduction in pneumonia-specific mortality (Mtango, 1986; Fauveau, 1992). For each of these estimates, one of the studies involved a combination of oral and injectable antibiotic treatment, while oral antibiotics only were used in the other studies.

This meta-analysis did not specifically address the issue of impact of oral compared to injectable antibiotics in the management of neonatal pneumonia. Moreover, the effects on neonatal mortality seen in the meta-analysis of pneumonia case management trials cannot be attributed directly to antibiotic effects, since a variety of other interventions accompanied use of antibiotics in the interventions areas. However, the diversity of the interventions included in the various packages and the variety of developing country settings, and the consistency of the impact in the various studies suggests that antibiotic treatment, which was common to all the trials, likely played a major role in mortality reduction. Moreover, it appears that a package of neonatal care interventions that includes oral antibiotic therapy likely will be beneficial in many developing countries. Finally, the reduction in neonatal mortality found in the meta-analysis was comparable to estimates of the proportion of neonatal illness due to

infections (i.e., 32%; Save the Children, 2001), suggesting that a substantial proportion of these deaths were averted through case management that included oral antibiotic therapy in the home.

Other data not included in the meta-analysis are available in which oral antibiotics have been used to treat neonatal infections, particularly pneumonia, in the community. In Indonesia, use of ampicillin plus supportive care (e.g., continued breastfeeding, clearing of the nose, fever control) in neonates with pneumonia had no measurable impact on cure rates of mild disease at 1-week follow-up, and did not halt progression to moderate disease at 1-week compared to the use of supportive care alone in the control group (Sutrisna, 1991). In Nepal, pneumonia case management using oral ampicillin (along with health education and immunizations) significantly reduced infant mortality in a before-after treatment design, although data specific to the neonate was not presented (Pandey, 1989).

Overall, oral antibiotics that have been used with some success to treat serious neonatal bacterial infections in the community include cotrimoxazole (Mtango, 1986; Khan, 1990a; Khan, 1990b; Bang, 1990; Bang, 1993; Bang, 1994; Bang, 1999; Pandey, 1991), cephalexin (Bhandari, 1996), penicillin (Datta, 1987), and ampicillin (Pandey, 1989; Fauveau, 1992). Supportive evidence is most extensive for cotrimoxazole, which is the agent recommended by the WHO for community management of infant and childhood pneumonia, and for most situations in which facility-based treatment of neonates with parenteral antibiotic is not feasible (WHO, 1995).

Conclusions on use of oral antibiotics for treatment of neonatal infections

Data from developed countries suggests that serious neonatal bacterial infections are best managed using parenteral antibiotics, and this standard of care should be provided when feasible in developing countries. For many families, however, facility-based care or even injectable antibiotics are not within their reach. Available data indicate that a case management approach that emphasizes essential newborn care along with prompt recognition of serious bacterial infections and treatment with oral antibiotics is superior to no case management. No data comparing oral and parenteral antibiotic treatment regimens in the community have been reported, and the incremental benefit of injectable over oral antibiotics is not known. In situations in which facility-based care and use of parenteral antibiotics are not feasible, however, clean delivery and healthful newborn care practices along with prompt recognition of and oral antibiotic therapy for potentially serious bacterial infections is likely to be of substantial benefit. Among oral agents, cotrimoxazole has the most extensive and most favorable record for community-based treatment of serious neonatal bacterial infections.

Choice of Oral Antibiotic for Home Use

A large number of oral antibiotics could be considered for treatment of infections in neonates. Principal factors to consider in choosing an oral antibiotic are outlined in Table 1.

Table 1. Considerations in selection of an oral antibiotic

I. Cost considerations
  a. Per-dose cost
  b. Number of doses, i.e. duration and frequency of treatment
II. Indirect costs
  a. Compliance
    i. Frequency and length of therapy
    ii. Palatability
  b. Incidence of treatment failure
  c. Incidence of adverse drug reactions, including drug interactions
III. Pharmacologic considerations
  a. Pharmacodynamic considerations
    i. Bactericidal
    ii. Intrinsic activity against pathogens
    iii. Beta-lactamase stability
    iv. Little potential for resistance development
  b. Pharmaceutic considerations
    i. Available in concentrated suspension
    ii. No direct gastrointestinal irritation
    iii. Food (i.e., breast milk) does not interfere with absorption
  c. Pharmacokinetic considerations
    i. High bioavailability
    ii. Rapid intestinal absorption
    iii. Good tissue penetration
    iv. Long elimination half-life

We have previously presented in-depth consideration of these factors and made detailed comparisons among available oral antibiotics (Darmstadt, 1997, Darmstadt, 1998). Salient features of oral antibiotics available in Bangladesh that could be considered for use in treating neonatal sepsis are summarized in Table 2.

Table 2. Comparison of Oral Antibiotics

Anti-biotic	Cost[1]	Activity			Prior use in neonates[2]	Tissue penetra-tion	CNS penetra-tion	Absorption from GI tract[3]	Dosing
		S. aureus	*S. pneumo*	Gram - bacilli					
Cotrimox-azole	$0.25	++	Rising resistance in some areas	++	Yes, most extensive	+++	++	+++	BID
Amoxi-cillin	$0.38	-	++	+	Yes	++	+	+++	TID
Amox-Clav	$1.00	+++	+++	+++	Yes	++	+	+++	BID
Cepha-lexin	$1.00	+++	++	+	Yes	++	+	++[4]	QID
Cefpro-zil	$1.00	+++	++	++	No	+++	++	+++	BID
Cefix-ime	$1.12	-	++	+++	No	++	++	+++	BID

Table 2 footnotes:
[1]Cost for a 10-day course of treatment in a 2-kg neonate.
[2]Refers to prior use in trials in developing countries.
3Considering bioavailability and impact of food on absorption.
[4]Absorption of cephalexin may be delayed and decreased by food, and may be decreased up to 50% in neonates.

The macrolides, such as erythromycin, were not included in this comparison due to insufficient spectrum of activity, significant gastrointestinal side effects, and association with emergence of antibiotic resistance. In general, the cephalosporins and penicillins both have a favorable side effect profile. There is extensive experience on use of cephalosporins and pencillins in neonates, as parenteral administration of penicillin/ampicillin or third generation cephalosporins (e.g., cefotaxime) is standard therapy for neonatal sepsis. The cephalosporins tend to be relatively expensive, however, and there is more concern over emergence of resistance with their use than with the penicillins. The first generation cephalosporins (e.g., cephalexin) lack sufficient activity against gram-negative pathogens, whereas the third generation agents provide excellent coverage against gram-negative organisms but activity against *S. aureus and S. pyogenes*, two of the most important agents of serious bacterial infections in young infants (WHO, 1999), may be compromised. For example, Cefixime, a third generation cephalosporin, entirely lacks activity against *S. aureus*.

Overall, the most promising oral agents for treatment of neonatal infections in the community are cotrimoxazole, the second-generation cephalosporins (e.g., cefprozil, cefuroxime, which can be considered interchangeable), and Augmentin. As noted above, cotrimoxazole has been used the most extensively and successfully in neonates in the community, and is the least expensive among these agents. Although it is not approved for use in neonates, it appears to have had a favorable safety record, and Bang et al. (1999) found no increase in jaundice among treated neonates. However, a primary concern with use of cotrimoxazole, suppression of the bone marrow, has not be monitored in community-based studies, and it is also unclear whether surveillance has been adequate to monitor the incidence of hypersensitivity reactions. The primary obstacle to use of cotrimoxazole in Bangladesh, however, is rising resistance among pneumococcal isolates over the past decade, such that the majority are now resistant and use of cotrimoxazole for treatment of community-acquired pneumonia in children is falling out of favor in Bangladesh (Saha, 1999). The second generation cephalosporins such as cefprozil and cefuroxime have a very favorable side effect profile and good spectrum of activity against most isolates of the principal agents presumed to cause serious bacterial infections in neonates in the community, namely *S. aureus*, *S. pneumoniae*, *S. pyogenes*, *E. coli*, *Salmonella*, *Enterobacter* and *Klebsiella*. They are relatively expensive, however, costing approximately $1 for a course of treatment. Augmentin is approved for use in neonates by the United States Food and Drug Administration, and, due to the extended spectrum afforded by competitive inhibition of beta-lactamases by clavalanic acid, is also active against most neonatal pathogens. The spectrum of activity of Augmentin, cefuroxime, and ceprozil are essentilly identical, and in clinical practice these agents are essentially interchangeable (Darmstadt, 1997). Augmentin is being produced in Bangladesh by reputable manufacturers, and generally costs slightly less (approximately $2 for a 10-day course of treatment for a neonate weighing 3 kg) than the cephalosporin class of antibiotics, with less concern about induction of resistance. When used prophylactically in women with preterm prelabor rupture of membranes (pPROM) in Zimbabwe, Augmentin (n=84; control, n=87) prolonged the period between rupture of membranes and labor, and there was a trend toward decreased neonatal sepsis (Magwali, 1999). In a much larger multi-center study of women with pPROM conducted largely in the United Kingdom, Augmentin (n=1205) prolonged pregnancy, and there was a trend toward reduction in neonatal bacteremia (p=0.2), especially if the baby was born within 14 days of presentation (p=0.08) (Kenyon, 2001a). Use of Augmentin, however was associated with an increased risk of necrotizing enterocolitis (NEC). Among 2394 and 2415 women treated with (either alone or in combination with erythromycin) or without any Augmentin, there were 44 (1.8%) and 17 (0.7%) cases, respectively, of proven NEC in their offspring (i.e., 27 additional

cases in the Augmentin group). Despite the increase in NEC, there was no significant difference between the two groups in neonatal mortality or in a composite of neonatal death, chronic lung disease or major cerebral abnormality (Kenyon, 2001). In another multi-center study in the UK in women with spontaneous preterm labor and intact fetal membranes, Augmentin did not impact neonatal bacteremia, neonatal mortality, or neonatal composite outcome, but again, was associated with an increase in incidence of NEC (i.e., 20 cases in 3085 patients who received Augmentin compared to 10 cases in 3156 patients who did not receive Augmentin) (Kenyon, 2001b). Overall, 37 additional cases of NEC occurred in 5480 neonates whose mothers received Augmentin compared to 5571 who received no Augmentin. To put this in perspective, recent data from India would suggest that among 5500 neonates (5500), 358 (6.5%) would develop serious bacterial infection requiring antibiotic therapy (Bang, 1999), and of these, approximately 48 would die from sepsis despite antibiotic treatment (case-fatality 13.3%, Bang, 1994), but the lives of 21 neonates would be saved due to oral antibiotic therapy (i.e., a 30% reduction in mortality, see meta-analysis above). Thus, the number of additional cases of NEC as a result of Augmentin therapy (n=27) appears to at least equal the projected number of lives saved as a result of case management with antibiotic therapy (n=21). This appears to be significant even when one considers that less than 4% of neonates in the SEARCH trial in India were in the higher risk category for NEC based on gestational age less than 34 weeks, since these few neonates accounted for approximately one-third of deaths. Taken altogether, although there is no direct data to suggest that treatment of neonates would result in a similar risk of NEC as when exposure occurs in utero, as in the two large multi-center trials, it seems prudent to avoid use of Augmentin. This is particularly true when other antibiotics with a comparable spectrum of activity but fewer safety concerns are available, such as second-generation cephalosporins, including ceprozil and cefuroxime.

Conclusions on Choice of Oral Antibiotic for use in Bangladesh

Considering the characteristics of, and available evidence for impact and safety of the various oral antibiotics available in Bangladesh, the best choice for treatment of serious neonatal bacterial infections appears to be a second generation cephalosporin that can be dosed twice daily, such as cefprozil or cefuroxime.

Dr. Saha and colleagues are currently conducting a study to determine the identity and antibiotic susceptibility profiles of the bacterial organisms that colonize the vagina of women in Mirzapur. The choice of antibiotics for the treatment of neonatal sepsis may be based on the findings of this study.

References

Ahmed S, Sobhan F, Islam A, e-Khuda B. Neonatal morbidity and care-seeking behaviour in rural Bangladesh. J Trop Pediatr 2001;47:98-105.

Bang AT, Bang RA, Baitule SB, Reddy MH, Deshmukh MD. Effect of home-based neonatal care and management of sepsis on neonatal mortality: field trial in rural India. Lancet 1999;354:1955-61.

Bang AT, Bang RA, Sontakke PG, SEARCH Team. Management of childhood pneumonia by traditional birth attendants. Bull WHO 1994; 72:897-905.

Bang AT, Bang RA, Morankar VP, Sontakke PG, Solanki JM. Pneumonia in neonates: can it be managed in the community? Arch Dis Child 1993;68:550-6.

Bang AT, Bang RA, Tale O, Sontakke PG, Solanki J, Wargantiwar R, Kelzarkar P. Reduction in pneumonia mortality and total childhood mortality by means of community-based intervention trial in Gadchiroli, India. Lancet 1990;336:201-206.

Bartlett AV, Paz de Bocaletti ME, Bocaletti MA. Neonatal and early postnatal morbidity and mortality in a rural Guatemalan community: the importance of infectious diseases and their management. Pediatr Infect Dis 1991;10:752-7.

Bhandari N, Bahl R, Bhatnagar V, Bhan MK. Treating sick young infants in urban slum setting. Lancet 1996;347:1774-1775.

Darmstadt GL. Oral antibiotics for uncomplicated bacterial skin infections in children. Pediatr Infect Dis J;1997;16:227-240.

Darmstadt GL. Antibiotics in the management of pediatric skin disease. Dermatol Clin 1998;16:509-525.

Datta N, Kumar V, Kumar L, Singhi S. Application of case management to the control of acute respiratory infections in low-birth-weight infants: a feasibility study. Bull World Health Organ 1987;65:77-82.

Fauveau V, Stewart MK, Chakraborty J, Khan SA. Impact on mortality of a community-based programme to control acute lower respiratory tract infections. Bull World Health Organ 1992;70:109-116.

Kenyon SL, Taylor DJ, Tarnow-Mordi W, ORACLE Collaborative Group. Broad-spectrum antibiotics for preterm, prelabour rupture of fetal membranes: the ORACLE I randomized trial. Lancet 2001a;357:979-988.

Kenyon SL, Taylor DJ, Tarnow-Mordi W, ORACLE Collaborative Group. Broad-spectrum antibiotics for spontaneous preterm labour: the ORACLE II randomized trial. Lancet 2001b;357:989-994.

Khan AJ, Khan JA, Akbar M, Addiss DG. Acute respiratory infections in children: a case management intervention in Abbottabad District, Pakistan. Bull World Health Organ 1990a;68:577-585.

Khan JA. Case management of acute respiratory infection in children of Abbottabad District, Pakistan: an intervention study. Bull Internatl Union Against Tuberculosis and Lung Dis 1990b;65:25-28.

Kielmann AA, Taylor CE, DeSweemer C, et al. The Narangwal experiment on interactions of nutrition and infections: II. Morbidity and mortality effects. Indian J Med Res 1978;68S:21-41.

Magwali TL, Chipato T, Majoko F, Rusakaniko S, Mujaji C. Prophylactic augmentin in prelabor preterm rupture of membranes. Intl J Gynecol Obstetr 1999;65:26q-265.

Mtango FDE, Neuvians D. Acute respiratory infections in children under five years. Control project in Bagamoyo District, Tanzania. Trans R Soc Trop Med Hyg 1986;80:851-858.

Pandey MR, Daulaire NMP, Starbuck ES, Houston RM, McPherson K. Reduction in total under-five mortality in western Nepal through community-based antimicrobial treatment of pneumonia. Lancet 1991;338:993-997.

Pandey MR, Sharma PR, Gubhaju BB, et al. Impact of a pilot acute respiratory infection (ARI) control programme in a rural community of the hill region of Nepal. Ann Trop Paediatr 1989;9:212-220.

Pratinidhi A, Shah U, Shrotri A, Bodhani N. Risk-approach strategy in neonatal care. Bull World Health Organ 1986;64:291-297.

Saha SK, Rikitomi N, Ruhulamin M, et al. Antimicrobial resistance ans serotype distribution of *Streptococcus pneumonia strains causing childhood infections in Bangladesh, 1993 to 1997. J Clin Microbiol 1999;37:798-800.*

Save the Children. State of the World’s Newborns. Save the Children Federation 2001;1-48.

Sazawal S, Black RE. Meta-analysis of intervention trials on case management of pneumonia in community settings. Lancet 1992;340:528-533.

Sutrisna B, Frerichs RR, Reingold AL. Randomized, controlled trial of effectiveness of ampicillin in mild acute respiratory infections in Indonesian children. Lancet 1991;338:471-474.

Zoysa ID, Bhandari N, Aktari N, Bhan MJ. Careseeking for illness in young infants in an urban slum in India. Soc Sci Med 1998;47:2101-2111.

WHO Young Infants Study Group. Bacterial etiology of serious infections in young infants in developing countries: results of a multicenter study. Pediatr Infect Dis J 1999;18:S17-22.

World Health Organization. The management of acute respiratory infections in children: practical guidelines for outpatient care. Geneva, Switzerland, WHO, 1995.

World Health Organization. Acute respiratory infections in children: case management in hospitals in developing countries. Geneva: WHO, 1990, WHO/ARI/90.5.

World Health Organization. Programme of acute respiratory infections. Report of the fourth meeting of technical advisory group 6-10 March 1989. Geneva, Switzerland, WHO, 1989, WHO/ARI/89.4.
