# Supplementary material for: Evaluation of a Cluster-Randomized Controlled Trial of a Package of Community-Based Maternal and Newborn Interventions in Mirzapur, Bangladesh
Source: PLoS One. 2010 Mar 24;5(3):e9696. doi: 10.1371/journal.pone.0009696 (PMC2844410; doi:10.1371/journal.pone.0009696)
Supplement: Reference S1 — Supporting document: Reference 21 - unpublished report (0.36 MB PDF) [file pone.0009696.s003.pdf]

Final Report: Formative Research on newborn care practices in the home and careseeking for sick newborns in Upazila Mirzapur, Tangail District, Bangladesh

November 2003

Final Report: Formative Research on newborn care practices in the home and careseeking for sick newborns in Upazila Mirzapur, Tangail District, Bangladesh

Contributors to this report

Tamanna Sharmin
Nabeel Ashraf Ali
M Habib Seraji
Sanwarul Bari
Peter Winch (Editor of overall report)

Data collection

Afsana Akther
Dilara Afroz
Dilruba Begum
Khaleda Akhter
Rowshan Jahan
Sitara-e-Zebin Khan

Data entry and tabulation

Jessica Greene
Nazma Begum

Principal Investigators

Gary L. Darmstadt
Shams El Arifeen

Co-Principal Investigators

Abdullah H. Baqui
Mathuram Santosham
Robert E. Black

Co-Investigators: Zahid Hassan, Peter Winch, M.Habib Seraji, Sanwarul Bari, Hugh Waters, Saifuddin Ahmed, K Zaman, Samir K Saha, Derrick Crook, Richard Moxon

---

Project to Advance the Health of Newborns and Mothers II (PROJAHNMO-II) – Upazila Mirzapur, Bangladesh

A collaborative project of:

Centre for Health and Population Research ICDDR,B
Johns Hopkins Bloomberg School of Public Health
Kumudini Welfare Trust and Kumudini Hospital
Bangladesh Rural Advancement Committee
Government of Bangladesh
Dhaka Shishu Hospital
Oxford University

Funding for the PROJAHNMO-II Project is provided by:

Burroughs Wellcome – Wellcome Trust Fund: Infectious Diseases Initiative

Table of Contents

1 INTRODUCTION TO THIS REPORT ... 8

1.1 OVERVIEW OF THE PROJAHNMO-II PROJECT IN UPAZILA MIRZAPUR ... 8
1.2 CARESEEKING DURING THE NEWBORN PERIOD ... 9
1.3 COMPARISONS WITH SYLHET DISTRICT, BANGLADESH IN THIS REPORT ... 10

2 METHODS OF DATA COLLECTION ... 11

3 SOCIAL AND ECONOMIC CHARACTERISTICS OF UPAZILA MIRZAPUR ... 12

3.1 ECONOMIC STATUS AND EDUCATION ... 12
3.2 TRANSPORT ... 13

4 TRADITIONAL BIRTH ATTENDANTS AND COMMUNITY HEALTH WORKERS ... 15

4.1 TRADITIONAL BIRTH ATTENDANTS (TBAS) - OVERVIEW ... 15
4.2 TRADITIONAL BIRTH ATTENDANTS (TBAS) – RESULTS OF SEMI-STRUCTURED INTERVIEWS ... 17
4.3 SHASTHO SHEBIKA IN MIRZAPUR ... 18
4.4 IMPLICATIONS FOR INTERVENTION DESIGN ... 19

5 ROLES OF HOUSEHOLD MEMBERS IN MATERNAL AND NEWBORN CARE ... 20

5.1 ROLES OF HOUSEHOLD MEMBERS IN MIRZAPUR ... 20
5.2 IMPLICATIONS FOR INTERVENTION DESIGN ... 23

6 PREGNANCY, LABOR, DELIVERY AND IMMEDIATE NEWBORN CARE ... 24

6.1 GENERAL PATTERN OF MATERNAL AND NEWBORN CARE PRACTICES IN MIRZAPUR ... 24
6.2 PRACTICES DURING PREGNANCY ... 25
6.3 LOCATION OF MOTHER AND CHILD DURING LABOR, DELIVERY AND THE NEONATAL PERIOD ... 29
6.4 KNOWLEDGE OF DANGER SIGNS AND CARE-SEEKING FOR COMPLICATIONS DURING LABOR AND DELIVERY ... 31
6.5 DELIVERY OF THE PLACENTA AND IMMEDIATE NEWBORN CARE ... 33
6.6 IMPLICATIONS FOR INTERVENTION DESIGN ... 34

7 ROUTINE NEWBORN CARE IN THE HOME ... 35

7.1 OVERVIEW OF NEWBORN CARE PRACTICES ... 35
7.2 BATHING, CARE OF THE UMBILICAL STUMP, THERMAL CONTROL AND SKIN CARE ... 36
7.3 PRE-LACTEAL FEEDS AND BREAST-FEEDING ... 41

8 THE SICK NEWBORN 44

8.1 ILLNESSES OF NEWBORNS AND CARESEEKING 44
8.2 SOURCES OF ADVICE AND CARE 47
8.3 RESULTS OF CARESEEKING SCENARIOS 47
8.4 ROLE OF TBAS IN REFERRAL OF SICK NEWBORNS 50
8.5 VILLAGE DOCTORS – GENERAL DESCRIPTION 51
8.6 ROLE OF VILLAGE DOCTORS IN MATERNAL CARE 52
8.7 ROLE OF VILLAGE DOCTORS IN NEWBORN CARE 53
8.8 IMPLICATIONS FOR INTERVENTION DESIGN 54

9 REFERENCES 57

List of Tables

Table 1. Three “Delays” Model for Health Care Seeking Behavior for Neonates 10
Table 2. Data collection instruments for the second round of formative research in Upazila Mirzapur 11
Table 3. Differences in social and economic environment between Sylhet District and Upazila Mirzapur 12
Table 4. Relative ease of transport from different Unions in Upazila Mirzapur to Mirzapur town 13
Table 5. Differences between traditional birth attendants in Sylhet and Mirzapur 16
Table 6. Reported roles of household members in labour, delivery and immediate newborn care, semi-structured interviews, N=40 21
Table 7. Reported roles of household members in routine newborn care, semi-structured interviews, N=40 22
Table 8. Household members reported to be primary and secondary caretakers of newborn during first eight days of life, semi-structured interviews, N=40 23
Table 9. Practices during pregnancy in Sylhet and Mirzapur 25
Table 10. Advantages and barriers to antenatal care attendance mentioned in semi-structured interviews 26
Table 11. Person Accompanying Woman on Antenatal Care Visits from semi-structured interviews, N=20 mentioned being accompanied on ANC visit (more than one answer allowed) 26
Table 12. Services reported to have been provided during antenatal visits, semi-structured interviews (N=26 women who mentioned having received antenatal care) 27
Table 13. Reported ways that foods are beneficial or detrimental to pregnant women, semi-structured interviews, N=40 27
Table 14. Classification of foods as good or bad for pregnant and breastfeeding women by respondents to semi-structured interviews, N=40 28
Table 15. Location of baby during first 8 days of life, semi-structured interviews, N=40 29
Table 16. Location of labor and delivery and delivery position in Sylhet and Mirzapur 30
Table 17. Sleeping place of baby during first 8 days of life, semi-structured interviews, N=40 30
Table 18. Practices during labor and delivery in Sylhet and Mirzapur 31
Table 19. Reported complications and their severity during last pregnancy from semi-structured interviews, N=40 32
Table 20. Reported complications and their severity during labour and delivery from semi-structured interviews, N=40 32
Table 21. Delivery of placenta and cord care in Sylhet and Mirzapur 33

Table 22. Differences in routine newborn care between Sylhet and Mirzapur....35
Table 23. Bathing practices during the first week from the semi-structured interviews, N=40.......36
Table 24. Care of the umbilical area before the stump fell off from the semi-structured interviews, N=40....38
Table 25. Reported day the umbilical stump fell off from the semi-structured interviews, N=40..38
Table 26. Care of the umbilical area after the stump fell off from the semi-structured interviews, N=40....39
Table 27. Wrapping practices during the first week from the semi-structured interviews, N=40...39
Table 28. Feeding of newborn in Mirzapur and Sylhet....41
Table 29. Reported initial feeding of newborn from semi-structured interviews, N=40....41
Table 30. Careseeking practices in Sylhet and Mirzapur....44
Table 31. Illnesses affecting newborns according to informants in Upazila Mirzapur....45
Table 32. Preferred sources of care for three scenarios on careseeking from the semi-structured interviews, N=19....48
Table 33. Preferred mode of transport for three scenarios on careseeking from the semi-structured interviews, N=19....49
Table 34. Estimated cost of transport for three scenarios on careseeking from the semi-structured interviews, N=19....50

I Executive Summary

The Projahnmo-II Project in Mirzapur Upazila (Sub-District) of Tangail District in central Bangladesh includes two major components. First, the project is putting in place a community-based, prospective surveillance system aimed at determining the organisms causing serious infections in neonates. Concurrently, the project is implementing an intervention aimed at reducing neonatal mortality through provision of essential obstetric and newborn care; identification of neonatal sepsis in the community; referral and community-based transport of sick neonates and strengthening of neonatal care in health facilities. These studies will provide a foundation for future programmatic implementation of appropriate management strategies for prevention and treatment of infections in neonates in the community.

This report describes the results of formative research conducted as part of this study. The purpose of the formative research was to provide the project team with information useful for the design of both an effective community-based surveillance for the identification of sick newborns, and also an effective intervention for intervening at the household level to reduce overall neonatal mortality, as well as mortality due specifically to neonatal infections. The research described here was conducted between November 2002 and April 2003.

Upazila Mirzapur (sub-district) was divided into 5 zones by the qualitative research team. A village was chosen in each zone, and a series of interviews conducted in each village, in order to sample the cultural and ecological diversity of the study area. The formative research was conducted in two phases. The first phase starting in November 2002 was exploratory and relatively unstructured. Twenty-six interviews were conducted with mothers of newborns, mothers-in-law, husbands and traditional birth attendants. Building on the results of this initial round of research, a second round of interviews started in January 2003. Key issues identified in first round that were investigating in more depth in the second round included family structure and responsibilities; organization of pregnancy, delivery and neonatal period; care for sick newborns and current and potential roles of traditional birth attendants, community health workers of BRAC (shastho shebika) and village associations is promoting better newborn care practices and facilitating careseeking for sick newborns.

In general the situation in Upazila Mirzapur is much more favourable for the promotion of appropriate newborn care practices than in Sylhet District. Along a continuum of ideal practices to harmful practices, households in Mirzapur are much closer to the ideal practices than are households in Sylhet, although there is still room for improvement on a large range of practices in Mirazpur.

In most cases the parturient woman goes to her natal home (baperbari, marbari) to deliver the baby and spend the postpartum period. The mother's natal home is usually in either the same union or an adjacent union. This fact means that a high proportion of deliveries will probably occur in a different study arm, i.e. women living in the intervention zone arm will delivery in the comparison zone and vice versa. The intervention plan must take this reality into account.

There is good awareness of the importance of antenatal care, danger signs during pregnancy, labor and delivery and willingness to seek care. Careseeking for prolonged or difficult labor may mean seeking oxytocin injections from a village doctor, a practice which the intervention should discourage. Care of the newborn immediately after birth is deficient. Being placed on the ground increases the risk of hypothermia, and also makes it less likely that breathing problems will be identified and acted upon immediately.

Routine newborn care practices are similar in Sylhet and Mirzapur including bathing the baby immediately after cutting the cord, massaging the baby with mustard oil, and performing a naming ritual around the end of the first week. In contrast to Sylhet, families in Mirzapur have fairly good knowledge of danger signs during pregnancy, labor, delivery and the newborn period. They are more aware of pneumonia and diarrhea as problems of newborns, perhaps influenced by various nation-wide campaigns on child health. There is limited awareness of other major causes of mortality in newborns such as asphyxia and hypothermia.

The main sources of care are Kumudini hospital and village doctors who are unlicensed and have little or no training. The influence of village doctors is pervasive. They need to be taken into account in the design of the intervention. They have the potential to provide messages on birth preparedness and newborn care especially to men when they visit the shops (dispensary/pharmacy), and to families when the VDs go out on calls. They could also paly an important role in the referral of mothers and newborns. TBAs could also play a key role in referral system. They need more education on danger signs.

In Mirzapur TBAs appear to generally play a positive role. Many are trained, and their training is valued by families. Many already perform antenatal and postnatal visits. Neonatal health interventions could build on this already-established pattern of visits, and insert additional content related to promotion of appropriate newborn care practices. Also, TBAs were found to prefer referral rather than taking risks. At the same time, the larger objectives of training for many TBAs is to become skilled in more complex procedures and acquire the necessary equipment and supplies to carry out these procedures. Program planners need to be aware of this, and deal with it directly in the course of the training. The shastho shebika have a wide range of training and carry out a wide range of types of work. Their role in promoting appropriate maternal and newborn care practices might include both dissemination of key messages and negotiation with families about changes in behavior, as well as sale of commodities such as birth kits or other items related to newborn care.

1 Introduction to this report

1.1 Overview of the Projahnmo-II Project in Upazila Mirzapur

Despite declines in infant mortality rates in recent decades, neonatal death rates (i.e., death in the first 28 days of life) remain unacceptably high. Deaths in the neonatal period (first 28 days of life) have been estimated to account for between 33 and 42% of the annual 10.8 million deaths occurring globally in children under five years of age (Black *et al.*, 2003). A large proportion of these deaths are due to sepsis and other infections. Investigation of the etiology of newborn infections and the effectiveness of interventions to prevent or treat newborn sepsis is therefore one of the top priorities in neonatal health research (Darmstadt *et al.*, 2000; Marsh *et al.*, 2002; Moss *et al.*, 2002; Darmstadt *et al.*, 2003). The only large prospective study to date which precisely identified the etiologic agents of neonatal infections was conducted by the World Health Organization (WHO) in Ethiopia, The Gambia, Papua New Guinea, and The Philippines in 1990 to 1993 (Lehmann *et al.*, 1999; WHO, 1999b; WHO, 1999a). This study was based on laboratory diagnostic testing in conjunction with medical history and clinical examination.

Bangladesh is one of the most densely populated of the low-income countries, and has among the highest perinatal mortality rates in the world (Baqui *et al.*, 1998; Baqui *et al.*, 2001). The risk of death in Bangladesh during the first month of life (48 per 1,000 births) is greater than in the next 11 months combined (34 per 1,000 births) (Islam *et al.*, 1982a; Islam *et al.*, 1982b; Mitra *et al.*, 1998). Thus, about 40% of all under-five deaths or about 60% of all infant deaths occur in the first month of life. Overall, one in 20 children born in Bangladesh dies during the neonatal period.

The Projahnmo-II Project in Mirzapur Upazila (Sub-District) of Tangail District in central Bangladesh includes two major components. First, the project is putting in place a community-based, prospective surveillance system aimed at determining the organisms causing serious infections in neonates. Concurrently, the project is implementing an intervention aimed at reducing neonatal mortality through provision of essential obstetric and newborn care; identification of neonatal sepsis in the community; referral and community-based transport of sick neonates and strengthening of neonatal care in health facilities. These studies will provide a foundation for future programmatic implementation of appropriate management strategies for prevention and treatment of infections in neonates in the community.

This report describes the results of formative research conducted as part of this study. The purpose of the formative research was to provide the project team with information useful for the design of both an effective community-based surveillance for the identification of sick newborns, and also an effective intervention for intervening at the household level to reduce overall neonatal mortality, as well as mortality due specifically to neonatal infections. The research described here was conducted between November 2002 and April 2003. We would like to thank Nazma Begum and her colleagues in the data management section for performing the data entry of the lengthy semi-structured instrument, Jessica Greene for making tabulations of the semi-structured data, and the qualitative interviewers Afsana Akther, Dilara Afroz, Dilruba

Begum, Sitara-e-Zebin Khan, Rowshan Jahan and Khaleda Akhter for their long hours in the field under difficult conditions. The trial and this formative research are part of the PROJAHNMO-II project, funded by the Burroughs Wellcome – Wellcome Trust Fund, Infectious Diseases Initiative.

1.2 Careseeking during the newborn period

Understanding the patterns and determinants of careseeking during the newborn period is important to this project for two reasons. First, the project needs to encourage families to bring sick newborns to Kumudini Hospital in Mirzapur so that blood, urine and other samples can be taken to test for a range of potential pathogens, and to provide the newborns with adequate treatment for their infections. Since a wide range of previous studies have demonstrated that families prefer to keep babies inside the home during the first 30-40 days of life even when they are ill (Islam *et al.*, 1982a; Blanchet, 1984; Jordan, 1987; Jeffery *et al.*, 1989; Jordan, 1993; Sutrisna *et al.*, 1993; Chawla, 1994; Cosminsky, 1994; Davis-Floyd & Sargent, 1997; Kaosar & Rashid, 2000; Winch *et al.*, Manuscript in preparation), it was anticipated that convincing families to seek care outside the home would be a challenge. Second, the project is seeking to intervene to improve the management of sick newborns as one component of a larger strategy to decrease neonatal mortality (Marsh *et al.*, 2002). Understanding how families assess whether newborns are sick and what factors affect where they seek treatment is necessary to design a more effective intervention. There is evidence from Sri Lanka and elsewhere that increases in careseeking are a major pathway contributing to decreased neonatal and infant mortality (McGilvray, 1994).

Health-seeking practices and barriers to referral have been a major impediment to facility-based care of sick neonates (de_Zoysa *et al.*, 1998; Ahmed *et al.*, 2001). The "Three Delays" model is widely used for the planning of interventions to decrease maternal mortality as shown in the following table (Barnes-Josiah *et al.*, 1998). The model proposes that maternal mortality is mostly due to delays in: 1) deciding to seek appropriate medical help for an obstetric emergency; 2) reaching an appropriate obstetric facility; and 3) receiving adequate care when a facility is reached. This framework can be adapted for neonatal care interventions that aim to promote early and appropriate care for sick neonates at health facilities.

Activities to improve the of care for sick neonates may include organization of a community-based system of emergency transport for sick infants, education of parents and community health workers, and institution of a system of referral slips (Nordberg *et al.*, 1996; Kalter *et al.*, 1997; Macintyre & Hotchkiss, 1999; Hotchkiss *et al.*, 2000). Recent research in Imbabura, Ecuador conducted as part of a WHO multi-country study on referral suggests that using referral slips, rather than simply making a verbal referral makes the caretakers significantly more likely to comply with referral (Kalter *et al.*, 2003).

Table 1. Three "Delays" Model for Health Care Seeking Behavior for Neonates

Where does delay occur	Example s of actions to address this delay
Decision in the home to seek appropriate medical help	- Education of parents and community health workers about danger signs in neonates that require immediate care at a health facility<br>- Education about improvements to quality of care in health facility<br>- Institution of a system of referral slips for community health workers
Transport from home to facility providing appropriate care for neonates	- Establishment of community-based systems of emergency transport for sick children<br>- Establishment of community funds to pay for transport of sick children
Provision of adequate care for neonates in health facility	- Training of facility-based health workers in the management of sick neonates<br>- Improved communication between health workers and parents of sick children

1.3 Comparisons with Sylhet District, Bangladesh in this report

This study is occurring at the same time at another large community-based neonatal care intervention trial with many of the same partner institutions in Sylhet District in northeastern Bangladesh. During the course of the formative research in the two sites, many similarities but also but surprising divergences in newborn care practices were documented between the two sites. These differences are discussed at some length in some sections of this report. They are significant in that they may indicate the need to implement newborn health interventions strategies differently in different settings, and also may help to explain variations in neonatal mortality from one site to another.

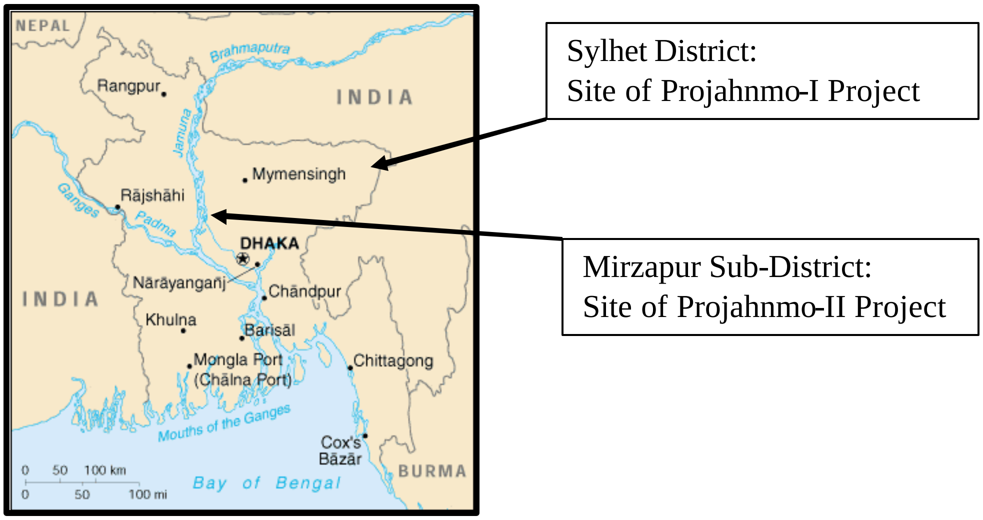

2 Methods of Data Collection

Upazila Mirzapur (sub-district) was divided into 5 zones by the qualitative research team. A village was chosen in each zone, and a series of interviews conducted in each village, in order to sample the cultural and ecological diversity of the study area. The formative research was conducted in two phases. The first phase starting in November 2002 was exploratory and relatively unstructured. Twenty-six interviews were conducted with mothers of newborns, mothers-in-law, husbands and traditional birth attendants using instrument Q1. The numbers from each group interviewed were:

- Recently-delivered women 10
- Husbands 5
- Mothers-in-law 5
- Traditional birth attendants 6

The interviews adopted a life cycle approach, starting with the informant's recollections of maternal and child health practices when he/she was a child, then asking about earlier pregnancies before dealing in more detail with the current pregnancy, delivery and care for the newborn. Interviews generally lasted from one to two hours and generated twenty to sixty pages of notes written in Bangla.

Building on the results of this initial round of research, a second round of interviews started in January 2003. These interviews were semi-structured, and included detailed questions on the roles of various family members. These interviews took 1.5 to 3 hours to administer, and sometimes had to be administered over the course of 2 or 3 sessions. Key issues identified in first round that were investigating in more depth in the second round included family structure and responsibilities; organization of pregnancy, delivery and neonatal period; care for sick newborns and current and potential roles of traditional birth attendants, community health workers of BRAC (shastho shebika) and village associations is promoting better newborn care practices and facilitating careseeking for sick newborns. The instruments used in the second round are listed below, and are available upon request.

Table 2. Data collection instruments for the second round of formative research in Upazila Mirzapur

	Sample size
Q2 – Routine care for newborn	40
Q3 – Care for sick newborns	19
Q4 – Maternal care practices	40
Q5 – Maternal and newborn care scenarios	19
Q6 – Traditional birth attendants	19
Q6 – Community health workers (CHWs) of BRAC (Shastho shebika)	9
Q6 – Village associations (somobay somiti)	16
Q7 – Village Doctors	10

3 Social and economic characteristics of Upazila Mirzapur

3.1 Economic status and education

Mirzapur Upazila (Sub-District) is located in central Bangladesh, less than 2 hours north of the capital Dhaka. Its central location and good transportation links with Dhaka have meant that the Mirzapur area is closely linked to the economic changes occurring in central Bangladesh, including the growth of the textile and other industries. The next table summarizes some of the social and economics differences between Sylhet and Mirzapur that were apparent from the formative research. Many of these differences will be confirmed quantitatively at a later point when results from the baseline surveys in the two sites are compared.

Table 3. Differences in social and economic environment between Sylhet District and Upazila Mirzapur

	Sylhet District	Upazila Mirzapur, Tangail District
Neonatal mortality	- Higher<br>- NMR = 81.7/1000 live births for Sylhet Region in Bangladesh DHS 1999/2000	- Lower<br>- NMR = 51.8/1000 live births for Dhaka Region in Bangladesh DHS 1999/2000
Patterns of fertility	- Higher<br>- TFR = 4.1 for Sylhet Region in Bangladesh DHS 1999/2000<br>- Low use of family planning indicated in interviews<br>- Informants indicated higher demand for children, think children will bring income in future	- Lower<br>- TFR = 3.2 for Dhaka Region in Bangladesh DHS 1999/2000<br>- Informants indicated lower demand for children, want 2-3 only
Sources of income cited in interviews	- Remittances from expatriate workers	- Agriculture<br>- Biscuit, pharmaceutical, textile and cotton factories or mills in Mirzapur<br>- Export processing zone one hour from Mirzapur, many people work there<br>- Cottage industries: production of cloth with hand-looms – sari, lungi
Female literacy	- Women usually not educated, low literacy rate for women observed in interviews	- Higher literacy rate for women, both men and women usually educated observed in interviews
Communication	- Lower exposure to mass media<br>- 62.1% of women have no exposure to mass media for Sylhet Region in Bangladesh DHS 1999/2000	- Greater exposure to mass media<br>- 49.0% of women have no exposure to mass media for Dhaka Region in Bangladesh DHS 1999/2000
NGOs	- Fewer NGOs	- Many NGOs BRAC, Asha, Proshika working on health and microcredit

While the formative research found that women in Sylhet District tend to stay at home, and in many cases wait for remittances to be sent from abroad, many men and women in Mirzapur leave the home on a daily basis to work in one of the local factories, or in the export processing zone about one hour from Mirzapur. Literacy rates are apparently much higher in Mirzapur than in Sylhet as there are more NGOs working and great exposure to the mass media.

The qualitative team found that there is much greater awareness of family planning and birth control in Mirzapur than in Sylhet, with couples generally expressing the desire to limit family size. A number of women reported both spontaneous and induced abortions. One woman reported having had three abortions. Abortions were rarely mentioned in Sylhet. Levels of fertility in Mirzapur appear to be similar to those of Bangladesh as a whole, and much lower than in Sylhet.

3.2 Transport

Transport is a key issue for the Mirzapur study. The study protocol calls for families to bring sick newborns to Kumudini Hospital in Mirzapur. The feasibility of this depends greatly on the available means of transport. The qualitative team found that transport if Mirzapur is much more difficult than in Sylhet. The roads are in worse condition, but many potholes and culverts. When a rickshaw comes to a culvert, the passengers needs to get out and walk over the culvert. Only the Dhaka-Mirzapur highway is in good condition.

Many methods of transport are available. Some people use 3 or 4 methods of transport to get from their village to Mirzapur as the condition of the road changes along the way. Methods of transport available include: trolley (three wheel rickshaw van), rickshaw, ox-cart, van, boat, and tempo (12 seat baby taxi). The condition of the tempos is very bad. They are first used for highway travel. Later when their condition has deteriorated, they are used for transport on village roads. Unlike Sylhet, microbuses (Lite Ace) are not widely available.

In remote villages it is sometimes hard to identify the road. The roads are very narrow, there are many trees, and the interviewers had to ask villagers which path was in fact the road. Roads are similar in condition to the Matlab comparison area, and similar to most of Bangladesh, but much worse than in Sylhet. The most difficult unions for communication are: Ajgana, Bastail, Anaitara and Uarsi. Ajgana Union is largely forested, with small fields carved out within the forest. Better unions for communication are near main road in centre of the Zila: Fatehpur, Jamurki, Mahera (Site of Police training centre, has better road), and Gorai.

Table 4. Relative ease of transport from different Unions in Upazila Mirzapur to Mirzapur town

Ease of transport	Easy	Difficult	More difficult	Impossible
Union	Jamurki<br>Mahera<br>Fatehpur<br>Bhatgram	Bahuria<br>Banail<br>Tarappur<br>Gorai	Ajgana (forest)<br>Uarsi<br>Anaitara<br>Bastail	Some villages in every union: Banaitoli, have to cross two rivers then walk

In general transport is easier in the rainy season, as people can use shallow-draught boats and are less dependent on the roads.

There are fixed times in some villages for transport e.g. two trips per day. This can result in long delays in seeking care. In one interview where a woman described careseeking for a retained placenta, she left the house at 2am and arrived at Kumudini Hospital at 7 am.

4 Traditional birth attendants and community health workers

4.1 Traditional birth attendants (TBAs) - Overview

There are a number of potential obstacles to achieving the objectives of the Projahnmo-II project. One of these is that when mothers or newborns exhibit danger signs and care is sought outside the home, often this care is delivered by TBAs, unlicensed medical practitioners or "village doctors" and traditional healers. This can make it impossible for the project to collect samples from newborns with signs of infection, as well as delay or replace definitive treatment for newborn infections in health facilities. For that reason, considerable effort was invested in understanding the roles, practices and skill levels of different types of providers that attend women during labor and delivery and provide treatment for sick newborns, most notably traditional birth attendants (TBAs) and village doctors. This section of the report presents data on TBAs and community health workers.

Data on roles and practices of TBAs in Mirzapur came from several different sources:

- First round of formative research (Unstructured interviews)
  - Statements by mothers of young children and other family members about role of TBA from the first round unstructured interviews
  - Six narrative-style interviews with TBAs in 1st round
- Second round of formative research
  - Semi-structured interviews using a 10-page instrument with 19 TBAs in 19 villages distributed among 12 unions in Mirzapur Thana. For these interviews the qualitative interviewers selected a trained TBA if there was one in the village, and otherwise selected an untrained TBA.

There are many differences between traditional birth attendants in Sylhet and Mirzapur with respect to numbers, motivation and training, and these differences have important implications for the design of the neonatal care intervention package for the Mirzapur study.

In Sylhet, many births are attended by non-trained family members or untrained traditional birth attendants. Many if not most of the women who assist with deliveries do not self-identify as TBAs, as the work of a TBA is considered to be of low status. Families place more value on having someone known to the family assist with delivery, than on having that person be trained. As a consequence, there is a large population of women who attend births in Sylhet, and each individual woman attends a relatively small number of births per year.

In Mirzapur families express a clear preference for trained birth attendants. If a trained birth attendant is present in a village, then the untrained birth attendants apparently will get little business. Women are much more proud of being identified as traditional birth attendants. Almost every village is said to have a trained birth attendant, a finding that needs to be confirmed during the mapping and listing. TBAs have been trained by Kumudini Hospital or one of the several NGOs working in Mirzapur: BRAC, Asha and Proshika.

Some of the courses last 3 months, others last 1 month. They initially received materials, but have not been resupplied. The NGO is said to pay 300tk for attending the training.

TBAs in Sylhet are known as *dhoroni* or *dhonni* from the word "*dhora*" meaning "catch." TBAs in Mirzapur are known as *chauni*. In the semi-structured interviews, 12 out of the 19 TBAs preferred the term *chauni*, while 7 out of the 19 preferred the terms *dai* or *dhatri*. While the *dhonni* are reticent to refer women with complications during labour and delivery to the hospital, and try to manage complications such as prolonged labour, breech presentation and retained placenta at home, the *chauni* in Mirzapur refer complications early, and often accompany the woman to the hospital when she is referred. In Sylhet the parturient woman is usually the one to cut the umbilical cord, but in Mirzapur it is usually the *chauni* who cuts the cord.

In contrast to the *dhonni* in Sylhet, the *chauni* in Mirzapur commonly make postpartum visits. If the *chauni* lives far from the woman's house, she may only visit on the sixth day, but if she lives close she may visit more frequently during the first week. TBAs are more professionalized in Mirzapur than in Sylhet. They accept payment in cash or in kind, but are more likely to be paid in kind with a sari, lungi or soap. These and other differences between Sylhet and Mirzapur are summarized in the following table.

Table 5. Differences between traditional birth attendants in Sylhet and Mirzapur

	Sylhet	Mirzapur
Name used for TBAs	*Dhonni, dhoroni*	*Chauni, dai*
Typical number of trained TBAs per village	1-2	1-2
Typical number of untrained TBAs per village	2-10	2-3
Preference of families between trained TBA, untrained TBA and family member	1. Family member<br>2. TBA known to family, whether or not trained<br>3. Other TBA not known to family	1. Trained TBA<br>2. Untrained TBA<br>➔ Clear preference for and understanding of the importance of training
Who trained the TBAs?	Family Welfare Centres	FWC, Kumudini Hospital, BRAC and other NGOs
Referral of complicated maternal cases to hospitals	Rare, try to manage cases at home as long as possible	Common, refer early and often accompany the woman
Antenatal home visits performed by TBAs	No	Sometimes
Postnatal home visits performed by TBAs	Sometimes	Usually

4.2 Traditional birth attendants (TBAs) – Results of semi-structured interviews

The 19 TBAs interviewed in the semi-structured interviewed were mostly between 40 and 55 years of age.

40-44	4	60-64	2
45-49	2	65-69	2
50-54	6	>70	1
55-59	2		

Their level of education was low, 14 having no education, 3 have primary education and 3 having secondary education. Most (13/19) had attended one or more previous trainings for TBAs, typically a course lasting 3 days to 2 weeks on safe delivery practices. The courses were organized by CARE (3 TBAs), Kumudini Hospital (3), BRAC (3), Proshika (1), Grameen Bank (2), Save the Children (1) and Bastail Hospital (1). A couple of TBAs had attended longer courses. One had attended a one month training course, and another the 18-month training course for Family Welfare Visitors (FWVs). Sometimes the training had occurred 5 to 10 or more years ago, and few details of the training could be recalled. There was a clear appreciation among families of the importance of training.

> "I decided on my own to get the training. No one in our village was trained then. Therefore, when I learned the skill, I decided to do the job of *chauni* ...nowadays, people do not call upon the *chauni* who do not have training." - Afsana/Jobeda/Oct.28,2002

None of the TBAs interviewed in the semi-structured interviews work for an organization. All stated that they work largely voluntarily, and when they are paid it is on an informal basis. Families pay the TBAs, often in kind, when they are happy with their work. The TBAs in Mirzapur provide a wide range of services including antenatal home visits (16/19), assistance at delivery (19/19), postnatal home visits (14/19), sale and promotion of birth kits (6/19), promotion of tetanus toxoid vaccination (14/19), promotion of breastfeeding (18/19) and promotion of the introduction of solid food to children over 5 months of age (12/19). The number of antenatal home visits made ranged from 2-3 (2/19) to 3-4 (3/19) to 4-5 (7/19).

The first contact with the family is early in the pregnancy in about half the cases (9/19) and mid-way through the pregnancy in the remaining (10/19). TBAs stated they were most often contacted by the mother-in-law (16/19) but can also be contacted by the pregnant woman herself (9/19), the husband (8/19), another senior female in the household (8/19) or the pregnant woman's mother (5/19). There was a wide range in the reported number of deliveries conducted per year:

2-4 deliveries	4	20-25 deliveries	2
5-7 deliveries	6	40-45 deliveries	1
10-12 deliveries	6		

The actual delivery techniques used by TBAs in Mirzapur are a mixture of traditional techniques passed down to them by other TBAs and techniques learned during training:

> "My baby fell under (was displaced), so the chauni came and massaged some mustard oil and raised (to normal position) the baby. Then she asked for warm water – washed the blade, and then cut the cord. In order to get the placenta out, she asked me to blow inside an empty bottle and shoved a little hair inside my throat..." -Afsana//Oct.29,2002

Most of the TBAs interviewed perform postpartum visits. Topics covered during this visits include the importance of warm massages (shek) to the perineum (15/19), proper diet (5), being careful about where and how the woman moves (4), cleanliness (4), taking medicines properly (3), avoidance of heavy work (3) and menstruation (2). Topics specifically related to newborn care that are covered during postpartum visits include breastfeeding and feeding of colostrums to baby (7/19), testing the arms and legs of the baby (7), application of warm massages (sek deoa) and Gentian violet to the umbilical area (6), bathing and massaging of the baby with mustard oil (5), keeping the baby warm (5), feeding the baby misrir pani (4), feeding the baby boiled cow's milk/goat's milk or tejpata (2). It appears then that messages transmitted by TBAs are concordant with public health messages regarding clean delivery techniques, colostrum and care of the umbilical area, but discordant regarding prelacteal feeds and massaging with mustard oil.

Program planners often see TBAs as playing a role primarily in diffusion of educational messages and timely referral of obstetric emergencies to health facilities. TBAs in Mirzapur don't see their role in those terms. This is best exemplified by the kinds of additional training they requested, unprompted, during the semi-structured interviews including how to give injections (3/19), how to cut the cord (2/19), how to administer saline (1/19) and how to perform an episiotomy which they refer to as a "small Caesarian section" (1/19). It is also demonstrated by the types of equipment and supplies they requested including a blade (8/19), a bag or box to carry equipment (6), scissors (5), thread (5), injections and pain killers (3), clean cloths (3), gloves (3), Savlon (2), soap (2) and saline (2). The apparent aspirations of at least some of the TBAs to learn how to perform more complex procedures need to be addressed during the training, and the reasons why procedures such as injections of oxytocin or pain killers should not be performed by TBAs in the home need to be stated directly.

At the same time that some TBAs expressed an interest in training and equipment for performing more complex procedures, there was general recognition among the TBAs of the importance of referring complicated cases to health facilities. On the TBAs interviewed in the semi-structured interviews, 13 of the 19 stated that they had referred women to hospital during labor and delivery during the past, and 11 out of 19 had referred a sick newborn.

4.3 Shastho Shebika in Mirzapur

The NGO Bangadesh Rural Advancement Committee (BRAC) has a nation-wide network of community health workers known as shastho shebika (SS) from the words shastho (health) and sheba (to care for). A comprehensive evaluation of even part of this program is beyond the scope of this report. However, since the SS are potential collaborators in

implementation, it was thought to be advisable to interview at least a few of them to assess how they might relate to the intervention being implemented. A small sample of 9 shastho shebika from 9 different villages, located in 9 different unions, were interviewed. All were associated with BRAC and all were female. Their level of education varied, one having no education, 6 having primary education and 2 having secondary education. The names they used to refer to themselves were daktar apa (4/9), shashto shebika (3/9) and shastho apa (2/9). They mentioned (unprompted) having taken a very wide range of training courses in the past, including training on treatment of common illnesses (2/9), TBA training through Save the Children and the Ministry of Health (2), AIDS training (1), shastho shebika training (1), vaccination (1), injections and administration of saline (1), veterinary training (1) and seed/agriculture and general hygiene (1). All but the TBA training were organized by BRAC according to the shastho shebika.

The shastho shebika interviewed mentioned involvement in a wide range of services including supporting vaccination/polio activities (8/9), promotion of child health in general (7), maternal health promotion (6), STD and HIV prevention (4), agriculture (4), microcredit (4), community mobilization (3) and sale of medicines (1). In relation to maternal care, all nine stated that they make home visits to pregnant women, seven stated that they attend deliveries and six make postpartum visits. The role of the shastho shebika in delivery care is typically one of helping the person attending the parturient woman rather the performing the delivery itself. Sources of income for their work mentioned by the shastho shebika are accompanying a women for her antenatal care visit, for which she is paid 2 taka, and a 25% profit from the sale of drugs.

4.4 Implications for intervention design

In Mirzapur TBAs appear to generally play a positive role. Many are trained, and their training is valued by families. Many already perform antenatal and postnatal visits. Neonatal health interventions could build on this already-established pattern of visits, and insert additional content related to promotion of appropriate newborn care practices. Also, TBAs were found to prefer referral rather than taking risks. At the same time, the larger objectives of training for many TBAs is to become skilled in more complex procedures and acquire the necessary equipment and supplies to carry out these procedures. Program planners need to be aware of this, and deal with it directly in the course of the training.

The shastho shebika have a wide range of training and carry out a wide range of types of work. They are all involved in maternal and newborn care, but somewhat less than are the TBAs. Two of the shastho shebika also work as TBAs. Their role in promoting appropriate maternal and newborn care practices might include both dissemination of key messages and negotiation with families about changes in behavior, as well as sale of commodities such as birth kits or other items related to newborn care.

5 Roles of household members in maternal and newborn care

5.1 Roles of household members in Mirzapur

In neonatal care, organization of the household and the roles of different household members are important considerations. A particular concern is to define who is responsible for care of the baby during the first month of life. While it may be the mother, in some cultures other household members take the lead role at this time, and need to be reached by interventions to improve newborn care. In neonatal care organization of the household and the roles of different household members are important considerations. A particular concern is to define who is responsible for care of the baby during the first month of life. While it may be the mother, in some cultures other household members take the lead role at this time, and need to be reached by interventions to improve newborn care.

The next three tables illustrate the varied roles of three groups of people involved in labour, delivery and newborn care:

1. Trained and untrained health workers: traditional birth attendants (TBAs) or *chauni* and nurses or doctors;
2. The mother of the newborn and females from her family such as her mother and her sister; and
3. The father of the newborn and females from his family such the child's grandmother/mother's mother-in-law or *shashuri*, the wife of the father's brother or *jal*, the wife of the father's father's brother or *chachi shashuri* and the younger sister of the father or *nonod*.

The role of these various household members in newborn care depends in part on where the mother and child stay. The mother has the greatest responsibility for newborn care regardless of where she stays. If the mother (with the child) stays at the *shashurbari* or husband's family compound, she gets some help from her mother-in-law (*shashuri*) and other female members of family such as *nonod* (husband's sister), *jal* or *ja* (husband's brother's wife), *chachi shashuri* (wife of husband's father's brother).

The major participants in decision-making are the senior females, as shown in Table 2. The major financial contributors are the husband and the husband's brother. Thus efforts to improve careseeking for maternal and neonatal emergencies must involve both senior females, the husband and the husband's brother.

The next table shows that the TBAs and the family of the parturient woman are much more involved in all aspects of care, compared to Sylhet District. One reason for this is the greater role of TBAs in Mirzapur and the respect for training in Mirzapur. Another reason is that women are much more likely to delivery their babies in their parents' home (*baperbari*), rather than their inlaws' home (*shashurbari*).

Table 6. Reported roles of household members in labour, delivery and immediate newborn care, semi-structured interviews, N=40

Action	1. Monitor progress of labour	2 Deliver baby	3 Deliver the placenta	4 Cut the umbilical cord	5 Help baby breathe	6 Wrap baby
**1. Health workers**						
Trained dhonni/TBA	6	16	13	14	8	12
Untrained dhonni/TBA	1	11	11	12	6	9
Nurse/daktar	5	6	6	7	4	6
**2. RDW/Mother of newborn and females from her household (*baperbari*)**						
RDW/Newborn's mother	0	1	1	1	0	1
Mother of RDW	9	2	3	1	2	3
Bon, Sister of RDW	2	0	1	1	0	1
**3. Father of newborn and females from his household (*shashurbari*)**						
Father/Husband	3	0	0	0	0	0
Shashuri, mother-in-law	4	1	1	2	1	1
Ja/Jal, Wife of husband's brother	4	0	2	0	0	1
Chachi shashuri, Wife of husband's father's brother	3	2	4	2	0	3
Nonod, Younger sister of husband						

The next table makes this point even more strongly. The mother of the parturient woman is very involved in all aspects of maternal and newborn care. It appears from this table that the three people with highest priority for messages about maternal and newborn care are the pregnant woman herself, her mother, and the TBA.

Table 7. Reported roles of household members in routine newborn care, semi-structured interviews, N=40

Relationship to recently-delivered woman (RDW)	1 Give baby something to drink		2 Give baby something to eat		3 Bathe baby		4 Put oil on skin of baby		5 Put clothes on baby	
	Little	A lot	Little	A lot	Little	A lot	Little	A lot	Little	A lot
**1. Health workers**										
Trained dhonni/TBA	5	0	1	0	10	0	7	0	8	0
Untrained dhonni/TBA	9	4	0	0	9	0	3	1	4	0
Nurse/*dakter*	2	0	0	0	2	1	2	0	4	0
**2. RDW/Mother of newborn and females from her household (*baperbari*)**										
RDW/Newborn's mother	7	15	6	21	1	14	11	24	12	26
Mother of RDW	9	8	10	4	4	20	7	18	8	17
Bon,Sister	2	2	3	0	3	1	4	3	6	3
Bhabi, elder brother's wife	1	1	0	0	2	1	4	0	3	1
Meye, daughter	2	1	1	2	1	0	3	0	4	0
**3. Father of newborn and females from his household (*shashurbari*)**										
Husband	0	0	1	0	0	0	0	0	1	0
Shashuri, mother-in-law	5	1	1	0	4	4	6	4	6	2
Ja/Jal, Wife of husband's brother	2	1	2	1	0	3	2	3	2	3
Chachi shashuri, Wife of husband's father's brother	1	0	0	0	5	0	3	0	2	0
Nonod, Younger sister of husband	0	0	0	0	0	0	1	0	1	0

The role of household members in newborn care depends on where the mother and child stay. The mother has prime responsibility for newborn care regardless of where she stays. Most deliveries and most newborn care occur in the at the baperbari or wife family compound. There she has many more people to lend a helping hand around her. Her own mother provides her with the most help, and this assistance is provided throughout the first week, as shown in the following table.

Table 8. Household members reported to be primary and secondary caretakers of newborn during first eight days of life, semi-structured interviews, N=40

Relationship to recently-delivered woman (RDW)	Care-taker type	Day of birth	Day 1	Day 2	Day 3	Day 4	Day 5	Day 6	Day 7	Day 8
**1. Health workers**										
TBA (Dhonni)	1°	0	0	0	0	0	0	0	0	0
	2°	0	0	0	0	0	0	0	0	0
Dakter/nurse	1°	6	4	2	2	2	1	1	1	0
	2°	0	0	1	1	1	2	2	2	2
**2. RDW/Mother of newborn and females from her household (*baperbari*)**										
RDW/Mother of newborn	1°	3	9	11	11	11	13	14	14	18
	2°	6	14	15	16	17	15	14	14	9
Mother of RDW (Baby's nani)	1°	7	15	16	17	17	16	14	14	11
	2°	9	4	4	4	4	4	4	4	4
Bon/Boin, mother's sister (baby's khala)	1°	3	1	1	1	1	1	1	1	1
	2°	2	5	4	3	3	3	2	2	1
**3. Father of newborn and females from his household (*shashurbari*)**										
Husband, father of baby	1°	3	5	4	3	3	2	2	2	1
	2°	2	1	2	2	2	3	3	3	4
Shashuri, mother-in-law (baby's dadi)	1°	3	5	4	3	3	2	2	2	1
	2°	2	1	2	2	2	3	3	3	4
Ja/Jal, Wife of husband's brother (Baby's chachi)	1°	2	2	2	2	2	2	2	2	0
	2°	4	2	3	3	3	3	3	3	3
Nonod, younger sister of husband (Baby's fupu)	1°	1	1	1	1	1	1	1	1	0
	2°	1	1	1	1	1	1	1	1	2

5.2 Implications for intervention design

The information presented in this section has a number of important implications for intervention design. The common practice of migration to the household of the pregnant woman's family for labor, delivery and the newborn period mean that it is crucial that the intervention reach women in family of the pregnant woman, especially her mother (baby's maternal grandmother). The larger roles of TBAs in care before, during and after birth again emphasize the need for the intervention to communicate with TBAs and engage them as full partners.

6 Pregnancy, labor, delivery and immediate newborn care

6.1 General pattern of maternal and newborn care practices in Mirzapur

In general the situation in Upazila Mirzapur is much more favourable for the promotion of appropriate newborn care practices than in Sylhet District. Along a continuum of ideal practices to harmful practices, households in Mirzapur are much closer to the ideal practices than are households in Sylhet, although there is still room for improvement on a large range of practices in Mirazpur.

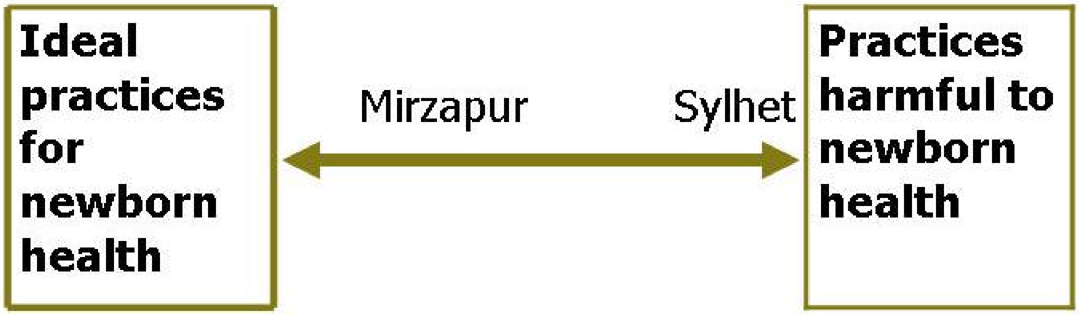

The situation in Mirzapur is generally favorable regarding careseeking for obstetric emergencies and for sick newborns. There is a positive view of health services, particularly of the quality of care at Kumudini Hospital. Informants expressed a preference for trained rather than untrained traditional birth attendants (TBAs) in interviews, a willingness to seek care outside the home, acceptance of biomedical interventions, and greater acceptance of family planning which is manifested in lower fertility rates and lower demand for children.

> "Kumudini is the best in Tangail. No matter which disease you go there with – they can cure it. Doctors and nurses are very good. They don't refuse anyone. Their behaviour is also very good. They care for you a lot and their treatment is good too. It's a clean hospital." - Dilara/Ghughi/Jan.28,2003

The generally favorable situation for the promotion of newborn health in Mirzapur appears to results from a number of different factors. Literacy rates are relatively high in Mirzapur. In addition, there have been many NGO projects in the past, and there continues to be a wide range of NGO activity. Past and current projects have involved NGOs such as BRAC, Grameen Bank, Proshika, Save the Children, CARE, SSS, Buro Tangail, and Udoy. Finally, many men work outside Upazila Mirzapur in the adjacent in export processing zone. While this is positive in terms of increased household income, it also has implications for community mobilization strategies and approaches to reaching men, as it will be less likely that men are home during the days, and community mobilization activities are more difficult to implement in the evenings.

6.2 Practices during pregnancy

The next table compares practices during pregnancy in Sylhet and Mirzapur. Women are much more likely to attend ANC in Mirzapur, but still deliver at home in almost all cases, similar to Sylhet.

Table 9. Practices during pregnancy in Sylhet and Mirzapur

	Sylhet	Mirzapur
Antenatal care (ANC)	Low use of ANC	High use of ANC but want to deliver in home
Food habits during pregnancy	Pregnant women avoid oily fish, many types of vegetables because they affect physical structure of baby. For example coconut water is said to make the baby's eyes white. If one cuts fish in shape of eclipse, baby will have cleft lip.	Avoid some kinds of food like duck, duck egg, carp, coconut, coconut water, pineapple<br>Some women eat pineapple, others say it destroys fetus and creates skin disease/pimples for baby<br>Coconut water makes baby have white eyes, others think coconut water is good, gives baby a bright face<br>Duck egg causes asthma<br>Mrigal fish (carp) will cause epilepsy (mrigiro)<br>More restrictions after delivery<br>One kind of eel, if eaten, will cause baby to twist
Work and movement during pregnancy	Concerned about being attacked by malevolent spirits or evil eye which can cause upri-batash.<br>To protect themselves, pregnant women never go out at time of call to prayer, carry amulets, piece of iron, or matches with them.	The concept of an illness called upri is not recognized.<br>Pregnant women try to avoid bao-batash (bad air), but don't take it as seriously though they try to avoid go out from their room at time of call to prayer.<br>During eclipse they have some rules for pregnant women. Pregnant women should not eat or cut anything during eclipse and she should walk throughout the time. She would not sleep but she can lie on her back. They cut a jute stick according to the height of the pregnant woman and place it in the ground in the middle of the courtyard.
Complications and danger signs during pregnancy	Headache, dizziness, vomiting, khichuni, jor, swollen hands, legs during pregnancy is a normal phenomenon for a woman.	Dizziness, vomiting, due to heavy work the fetus displace to lower abdomen (baccha tole pora), weakness are considered as complications during pregnancy.

Awareness of antenatal care and its benefits are high. The following table lists the advantages and disadvantages of antenatal care listed in semi-structured interviews. Women appreciated the concept of attending antenatal care to protect themselves and their babies, but sometimes could not attend because of the distance from their house to the site of ANC delivery, or because they are not allowed to leave their home unaccompanied.

Table 10. Advantages and barriers to antenatal care attendance mentioned in semi-structured interviews

Advantages of ANC mentioned<br>N=26 women who mentioned receiving antenatal care	Mentioned
• Protect baby & mother from disease	20/26
• Mother have healthy baby	19
• Recognize danger signs during labor	12
• Prevent caesarian & delivery complications	4
Barriers to ANC mentioned<br>N=12 women who mentioned not receiving antenatal care	
• Lack of freedom to leave house	5/12
• Distance to ANC facilities from home	5
• No one to accompany her	3
• Household work of higher priority	3
• Mother not consider ANC important	2
• Family members not allow	1
• Lack of money	1
• Doctor will give medicine she doesn't want to take	0
• Unfavorable environment of facilities	0
•Mother does not face problems and does not need ANC	0

Table 11. Person Accompanying Woman on Antenatal Care Visits from semi-structured interviews, N=20 mentioned being accompanied on ANC visit (more than one answer allowed)

	# Respondents
Husband	10
Brother (bhai)	2
Younger sister of husband (Nonod)	2
Wife of husband's father's brother (Chachi Shashuri)	2
Mother-in-law (Shashuri)	1
Wife of husband's brother (Ja/Jal)	1
Elder brother's wife (Bhabi)	1
Sister (Bon)	1
Aunt (Khala)	1

Women expressed the need to be accompanied when they went for antenatal care. The preceding table demonstrates that women are most often accompanied by their husbands, but that a variety of other women can also accompany them.

Table 12. Services reported to have been provided during antenatal visits, semi-structured interviews (N=26 women who mentioned having received antenatal care)

	Services mentioned
Weighing	25
Measure blood pressure	24
Check baby's position	25
Get iron tablets	20
Get other medication	9
Get vaccination	22
Other	2

The services most often reported to have been provided were weighing, measurement of blood pressure and checking of the baby's position.

Various foods are also believed to have a variety of adverse effects on the developing fetus and/or mother, or on the breastfeeding newborn. The second phase of formative research conducted in Mirzapur helped to delineate the perceived beneficial and harmful effects that various foods have on the mother and baby during pregnancy. The following Table outlines these perceived effects.

Table 13. Reported ways that foods are beneficial or detrimental to pregnant women, semi-structured interviews, N=40

**Ways foods can be beneficial**		**Ways foods can be detrimental**	
Good for health	39	Make blood thicker/watery	24
Increase energy	39	Cause cold for baby	24
Increase blood	20	Affect physical structure of baby	24
Purify blood	12	Cause allergy	22
Improve appearance of baby	10	Affect appearance of baby	18
Prevent colds	5	Cerate digestion	11
Help digestion	5	Cause batrog	11
		Increase size of baby	7
		Cause abortion	6
		Cause swollen arms/feet	1

Given that many foods are perceived to have harmful qualities, food restrictions were found to be common. The following two Tables outline the types of food mentioned by women during the second phase of formative research and whether they were deemed to be "good" or "bad." In common with rest of Bangladesh, people think that pregnant women should not eat large quantities of food for fear that the baby will grow too large and the woman will subsequently experience difficulties during delivery.

Table 14. Classification of foods as good or bad for pregnant and breastfeeding women by respondents to semi-structured interviews, N=40

Food	Suitability for pregnant women			Suitability for breastfeeding women		
	Good	Bad	No opinion as to whether good or bad	Good	Bad	No opinion as to whether good or bad
Vegetables	39	0	1	12	25	3
Grapes	18	0	22	11	3	26
Chicken	34	0	6	30	2	8
Eggs	32	1	7	26	3	11
Milk	28	0	12	28	2	10
Fish	28	5	7	15	6	19
Telal Fish	15	1	24	20	2	18
Small Fish	27	0	13	18	3	19
Dry Fish	8	4	28	1	13	26
Meat	25	6	9	10	18	12
Apple	22	0	18	11	1	28
Dal	25	2	13	24	5	11
Sour Foods	13	5	22	3	12	25
Potatoes	24	2	14	28	0	12
Coconut	13	19	8	6	7	27
Coconut Water	8	22	10	5	8	27
Gr. Pineapple	2	13	25	0	2	38
Tamarind	6	24	10	2	16	22
Duck	1	37	2	4	24	12
Duck Egg	2	37	1	4	24	12

The above table shows that coconut, tamarind and ducks and their eggs are particular concerns for pregnant women, and ducks and their eggs, dry fish, meat, vegetables, tamarind, and sour foods are concerns for breastfeeding women. The restrictions mentioned are less numerous than was found in Sylhet.

There appears to be a greater tendency to relieve the pregnant woman of some of her workload in Mirzapur, as shown in the following quote from a husband:

> "I asked her not to do any hard work during her pregnancy. I asked her not to fetch water, cook, or carry heavy sacks of rice. I fetched the water and carried the sacks of rice myself."
> - Afsana/Adabari/Oct.25,2002

6.3 Location of mother and child during labor, delivery and the neonatal period

The next table compares the location of the mother and child during labor and delivery in Sylhet and Mirzapur. Women in Mirzapur are much more likely to deliver in their mother's home (baperbari) rather than their mother-in-law's home (shashurbari) as compared to Sylhet. After delivery they stay in the baperbari for 40 days. There is little movement of the mother and newborn from one household to another during the first weeks of life.

Table 15. Location of baby during first 8 days of life, semi-structured interviews, N=40

Day of life of new-born	Household (bari) where baby stays			Room in the house where baby is located				
	Bari of mother's family. Baperbari	Bari of father's family, shashurbari	Other house-hold or no resp.	Mother's bedroom	Other room inside	Kitchen	Outside	Other or no response
DOB	20	12	8	7	13	7	7	6
Day 1	20	11	9	11	13	5	6	5
Day 2	21	11	8	11	14	5	6	4
Day 3	19	11	10	11	14	5	6	4
Day 4	21	11	8	11	14	5	6	4
Day 5	19	11	10	11	13	4	6	6
Day 6	19	12	9	14	11	3	5	7
Day 7	18	12	10	14	11	3	5	7
Week 2	19	9	12	12	11	1	5	11

In Sylhet the kitchen is more use as delivery room though the higher class-select their living room for that, often mother remains there with newborn for first 7-9 days. In Mirzapur the minority of birth take place in the kitchen. Those spaces in the house which are not used as living space may be used for delivery. After delivery they shift to mother's own room. Among Hindus and tribals , there is a delivery room called Chhotti ghor. They would not deliver the babies at their living room. At late pregnancy they prepare it.

The clear preference for the delivery position in Mirzapur is the squatting position, known locally as *jakon* or *boshay*:

> "When I got there, I saw she (untrained chauni) had the pregnant mother sitting up 'giving jakon' all night long. The mother was saying that they would kill her. You see, you need to have the pregnant woman walk before she sits in jakon and delivers. She needs to walk even if she is bleeding. So, I had her walk and the delivery happened smoothly." Trained TBA discussing practices of an untrained chauni - Afsana/Majalia/Oct.28,2002

Table 16. Location of labor and delivery and delivery position in Sylhet and Mirzapur

	Sylhet	Mirzapur
Household where delivery occurs	Usually in compound of husband's family (shashurbari)	Usually at compound of woman's family (baperbari)
Room in the house where delivery occurs	Typically kitchen	Typically room other than kitchen or main living room
Surface for delivery	They prepare a bed on the floor with bamboo/jute mat, sack or kantha (Tolpani) and for the first seven days after born the baby and the mother stay there.	They prepare a bed on the floor with a plastic sheet on a bamboo mat. After delivery if necessary, the baby can shift to bed but the mother should not leave the floor bed until 7days.
Delivery Position	Supine position is considered as ideal practice.	Delivery takes place by squatting position, which is called as Jakon/Boshay locally.

Babies most often sleep on a layered bed on the floor, as shown in the next table.

Table 17. Sleeping place of baby during first 8 days of life, semi-structured interviews, N=40

Day of life of newborn	Layered bed on floor				Total for layered beds	5= Mother's bed	6= Crib	7=Khat or choki	8=Other or no response
	1=Floor /Pati /Katha	2=Floor /Chhlaa /Katha	3=Floor /Pati /Chhala /Katha	4=Floor /Polythene /Chhala /Katha /Other					
DOB	3	0	4	15	22	0	0	5	13
Day 1	6	0	11	9	26	0	0	9	5
Day 2	6	0	12	9	27	1	0	9	3
Day 3	6	0	12	9	27	1	0	9	3
Day 4	5	0	12	9	26	1	0	10	3
Day 5	4	0	11	7	22	1	0	12	3
Day 6	2	0	10	6	18	1	0	15	3
Day 7	2	0	10	6	18	1	0	15	6
Week 2	1	1	8	4	14	0	0	18	8

6.4 *Knowledge of danger signs and care-seeking for complications during labor and delivery*

Women and their families interviewed in Mirzapur demonstrated greater awareness of danger signs and the need to seek care from a health facility when they occur, as summarized in the following table.

Table 18. Practices during labor and delivery in Sylhet and Mirzapur

	Sylhet	Mirzapur
Awareness of complications during labor and delivery	Less ANC visit, therefore less conscious about the complications during labor and delivery.	More ANC visits and greater awareness of the complications. They are also aware of danger of prolonged labor.
Complications during labor and delivery mentioned in interviews	- If hand/legs come first<br>- Pani bhanga<br>- Placenta previa<br>- Retained placenta<br>- Heavy bleeding	- Baccha chore pora (Premature leaking of water creates dryness inside the stomach<br>- Hand/leg comes first<br>- Prolonged labor<br>- Tetanus
Response by TBAs to obstetric complications	Usually TBA (dhonni) tries to deliver all the cases. For this they use amulets, Pani pora and other traditional treatments.	If TBA (chauni) finds that it is a complicated case then she usually refers to the hospital. TBA plays important role in decisions regarding outside careseeking.
C-section	People don't like at all and the rate is very low compare to MZP. Caesarian sections avoided at all costs.	Although they do not like C-sections, they are more flexible and will seek care for a C-section if there are danger signs.

The next two tables list danger signs during pregnancy and during labor and delivery that were mentioned by respondents to the semi-structured interviews, as well as their judgement as to whether they require care from a hospital. Many danger signs are recognized, but are treated outside of a health facility. A common solution used by families is to request an oxytocin injection from a village doctor:

> "The delivery was taking too much time. So I went and got the doctor. The doctor gave an injection, and then the TBA (chauni) delivered the baby nicely." - Rowshan/Ichail/Oct.20,2002

Table 19. Reported complications and their severity during last pregnancy from semi-structured interviews, N=40

Complication	Compli-cation reported	Severity if reported			Care from hos-pital?	Care from NGO clinic?
		Not severe	Mode-rately severe	Very severe		
Headache	16	8	5	3	4	0
Dizziness	13	5	6	2	2	0
Nausea	22	11	11	0	6	0
Swollen Arms/Feet	6	0	2	3	3	0
Weakness	26	3	17	5	6	0
No Appetite	8	3	5	0	1	0
Abdominal Pain	10	3	5	1	7	0
Fever	9	4	3	2	3	0
Sleepless	8	2	2	1	3	0
Lack Blood	7	2	3	2	4	0
Chick Mara	4	0	0	4	4	0
Baby Moves	0	0	0	0	0	0

Table 20. Reported complications and their severity during labour and delivery from semi-structured interviews, N=40

Complication	Compli-cation reported	Severity if reported			Care from hos-pital?	Care from NGO clinic?
		Not severe	Mode-rately severe	Very severe		
Breech presentation – bottom first (Ufta Bachha)	10	0	0	9	10	0
Water breaking (Pani Bhanga)	15	3	2	10	11	0
Hands/Feet First	14	0	0	14	14	0
Transverse lie (Pathailla Bachha)	2	0	0	2	2	0
Baby Swollen Hands/Feet	6	1	1	4	3	0
Bleeding	10	0	0	10	9	0
Fever	3	0	1	2	3	0
Convulsions (Chick Mara)	12	0	0	12	10	0
Prolong Pain	14	4	8	1	7	0
On & Off Pain	13	3	4	4	3	0
Delay Placenta Removal	10	0	0	9	8	0
Ripped cord (Nari Chera)	7	0	0	7	5	0
Mother Gets Fit	2	0	1	1	1	0
High BP	3	0	0	3	2	0

6.5 Delivery of the placenta and immediate newborn care

The table below summarizes differences in how the placenta is delivered and the umbilical cord is cut in the two sites. In both sites it is thought inappropriate to cut the umbilical cord before the placenta has been delivered. While awaiting delivery of the placenta, the newborn is usually kept on the ground, uncovered. In Sylhet the mother usually cuts the cord, while in Mirzapur the cord is usually cut by the TBA.

Table 21. Delivery of placenta and cord care in Sylhet and Mirzapur

	Sylhet	Mirzapur
Pulling on cord to facilitate expulsion of the placenta	No	No
Induce vomiting in woman to expel placenta	Yes: Insert hair deep into mouth or bind the stomach tightly	Yes: Insert hair deep into mouth, give mustard oil to drink, give garlic to ear
Insertion of hand into uterus to extract the placenta	Reported to be common	Reported to be rare because TBAs learn in their training to not do this
Location of newborn before cord is cut (awaiting delivery of placenta)	On sack or bamboo mat (delivery bed) without plastic sheet, never take baby on lap before cutting cord	On plastic sheet on delivery bed on the ground, sometimes held on lap
Heating of placenta and umbilical cord to stimulate breathing in baby	Yes	Yes
Who cuts the cord	Usually mother herself cuts the cord. If she is unable to do that then only the youngest child (not started praying) cut the cord. The person who cuts the cord can't pray until after 40 days.	Usually TBA (chauni) cuts the cord
How cord is cut	Cord cut with new but unboiled blade.	Cut cord with new, boiled blade
Cord care immediately after cutting	After cutting cord, they use turmeric juice for girl baby and ginger juice for boys and chewed rice on the umbilical stamp to stop the bleeding. They use fishing net thread to tie the cord. They give heat (Sek deoa) applied with piece of warmed cloth.	After cutting the cord they often don't apply anything to it. Sometimes they use Sindur (Red coloured powder use by the married hindu women), poramati, Khoer, mustard oil and crashed rotten betelnut.

The typical sequence of actions taken right after birth is:

1. Baby delivered and place on bed on the ground
2. Actions taken to facilitate delivery of the placenta such as inducing vomiting in the mother
3. Delivery of the placenta
4. TBA (Chauni) holds the baby
5. Usually the baby is given a bath – they skip bathing only if it is too cold
6. Wipe – with a soft cotton cloth
7. Massage mustard oil onto skin of baby
8. Wrap – with a soft cotton cloth, lungi or shari
9. Prelacteal foods given such as water mixed with palm sugar (misrir pani) or honey (modhu)
10. Colostrum (shaldudh) is then fed to the baby

Wrapping and feeding practices are described in more detail in the next section. Breathing difficulties immediately after birth are a recognized problem, and the TBA is the one responsible for care. Common practices to stimulate breathing in the newborn include heating the placenta or umbilical cord (resulting in a putative transfer to heat to the newborn), miling of the umbilical cord toward the baby, and holding the newborn upside-down and miling the cord.

> "...the baby came out but it wasn't breathing or crying, as if it had died. So the mother's grandma asked for water and started moving and shaking the baby in the cold water. In the meantime, others in the room, including the mother, were frying the placenta and milking the umbilical cord. I slapped the baby in the back and on the stomach. After about an hour of these activities, the baby started to breath faintly..."
> Dilara/Sinjhuri/Oct.27,2002

The TBAs do their best to manage breathing problems, but when they are unsuccessful families stated that they refer to newborn to a health facilities for care.

6.6 Implications for intervention design

Site of delivery. In most cases the parturient woman goes to her natal home (baperbari, marbari) to deliver the baby and spend the postpartum period. The mother's natal home is usually in either the same union or an adjacent union. This fact means that a high proportion of deliveries will probably occur in a different study arm, i.e. women living in the intervention zone arm will delivery in the comparison zone and vice versa. The intervention plan must take this reality into account.

Practices. There is good awareness of the importance of antenatal care, danger signs during pregnancy, labor and delivery and willingness to seek care. Careseeking for prolonged or difficult labor may mean seeking oxytocin injections from a village doctor, a practice which the intervention should discourage. Care of the newborn immediately after birth is deficient. Being placed on the ground increases the risk of hypothermia, and also makes it less likely that breathing problems will be identified and acted upon immediately.

7 Routine newborn care in the home

7.1 Overview of newborn care practices

Routine newborn care practices are similar in Sylhet and Mirzapur including bathing the baby immediately after cutting the cord, massaging the baby with mustard oil, and performing a naming ritual around the end of the first week. A significant difference is that Sylhetis are more likely to take sick newborns to a homeopathic doctor, whereas Mirzapuris are apparently more like to seek care from an allopathic practitioner.

Table 22. Differences in routine newborn care between Sylhet and Mirzapur

	Sylhet	Mirzapur
Baby's food	If the baby does not get sufficient breast milk then s/he will get banana, much saline, misrir pani, rice powder and wheat powder.	If the baby get sufficient breast milk then there is no extra food. If the baby does not get sufficient breast milk then s/he gets goat's milk, cow's milk and powder milk.
First bath	First bath just after cutting the cord for purifying the baby. They use warm water and soap for bathing. Vernix is being tried to remove during bathe with a soft, thin piece of cloth. After bath they massage mustered oil+garlic combination.	First bath just after cutting the cord. Though if the baby is sick or the weather is cold then they wait. They don't try to remove the vernix. They warm the water under sunlight and then put on fire. Before bath they wipe the baby with a piece of wet cloth.
Skin Care	To protect from cold they massage mustard oil+garlic allover the body.	They massage mustard oil all over the body but not protect from cold, to smooth the skin.
Wrapping	For the first seven days they cover the body of the baby though the head, neck and legs are uncovered.	For wrapping the baby, they use small kantha (patuni/duma) which are made from old clothes/lungies for seven days. After seven days they dispose this (patuni/duma).
Rituals	At 7/9th day of birth, "Noai" observed among muslims as well as hindues..	At 6th day of birth "Choy Soshthi" among hindues and at 7th day of birth muslims observe "Satura".
Illnesses recognised	Babies suffer in Sylhet from Hazma, Ulmi, Phera, Navipocha, Mukh Gha, Dasto, Neomonia, Upri laga, Sordikashi, Tap Utha within the 40 days of life.	Babies suffer from Sordikashi, jor, Dhonushtongkar, Dudh phera/Rokhha, Haga, neomonia, ham, mashipishi for first 40 days.
Food for breast feeding mother	For increasing the breast milk they eat Mouka fish, black cumin (kalo jeera), ginger paste, dry fish, potato and dry food. They perceived from this the body of the pregnant mother will cure soon. They also drink less water for the same reason.	Black cumin (kalo jeera), chili paste, garlic paste are given to the new mother to increase breast milk. At the first 40 days they usually don't eat any leafy vegetables because then the baby would have stomachache. They drink vegetable soup. They give pigeon, fish without skull like magur, shing, tangra, kalkini (young kalo boush fish) to increase breast milk and blood. New mother should avoid duck, duck's egg, beef and any sour food. They also drink less water.

7.2 Bathing, care of the umbilical stump, thermal control and skin care

Families recognize that coldness is a problem in newborns, and take action to prevent it. Practices to keep newborns warm include massaging with mustard oil twice a day (once in the morning after the bath and once in the evening), wrapping the baby with a soft cotton cloth, lungi or sari and application of a heat massage or *shek* twice a day (after the bath and in the evening). Families recognize that bathing can make the child cold, but feel bathing immediately after birth is crucial in order to make the child both physically and spiritually clean (*napak*) so it can be held by others. The next table shows that most babies are bathed on the day of birth, by the TBA, using warm water containing dettol.

Table 23. Bathing practices during the first week from the semi-structured interviews, N=40

Question	Response categories	Frequencies
On what day was baby first bathed?	DOB	33 (82.5%)
	Day 1	4 (10.0%)
	Day 2	1 (2.5%)
	Day 3	1 (2.5%)
	Day 4	1 (2.5%)
	Day 5	0
	Day 6	0
	Day 7	0
	Week 2	0
Why was baby first bathed on that day? (more than one answer allowed)	1=Baby was bathed later because she/he was vulnerable to cold if bathed earlier	3
	2=Cold Weather	8
	3= Baby was sick	0
	4=Baby should be cleaned as soon as possible to purify	28
	88= Other	3
How was baby prepared for bath?	Wipe the body of the baby before bath	24
	Performed Oju	14
	Covered the Umbilicus	3
	Massage Oil	1
	Preparation for protection from Upri	0
How was the water prepared? What was put in the water?	Warm water mixed with pond water or tube well water	29
	Put dettol in the water	21
	Clean the vessel	7
	Warm the water for bath	7

Question	Response categories	Frequencies
Who gave the baby its first bath? (More than one response permitted)	Chauni/TBA<br>Mother<br>Shashuri/mother-in-law<br>Ja/Jal<br>Baby's mother<br>Chachi-Shashuri<br>Bhabi<br>Khala/Aunt<br>Husband/baby's father<br>Mother's mother<br>Other	28<br>4<br>3<br>2<br>1<br>1<br>1<br>1<br>0<br>0<br>1
Was soap used?	Yes<br>No or no response	38<br>2
Was the vernix/white skin removed?	Yes<br>No<br>Don't know/no response	24<br>11<br>5
Who removed the vernix? (more than one answer allowed)	Chauni//TBA<br>Mother's mother<br>Baby's mother<br>Shashuri/mother-in-law<br>Khala/Aunt<br>Chachi-shashuri	17<br>5<br>2<br>2<br>1<br>1
What was done (to the baby) after the bath?	Wipe Baby<br>Massage mustard oil on skin<br>Wrap baby<br>Wipe Umbilicus/nabi<br>*Sek Deoa*<br>Massage baby oil on skin<br>Other<br>Massage *Tel Pora*<br>Massage other oil on skin	35<br>34<br>29<br>12<br>7<br>3<br>2<br>1<br>0
Number of baths in first week	1<br>2<br>3<br>4<br>5 or more	1<br>7<br>15<br>7<br>10

Care for the umbilical stump including application of heat (shek) using a piece of cloth or a fingernail and application of Gentian violet. If the umbilical stump does not fall off or does not health, then people will apply a red powder called shidur to the umbilical area, or apply ashed from the stove. The following table summarizes umbilical care practices in Mirzapur.

Table 24. Care of the umbilical area before the stump fell off from the semi-structured interviews, N=40

Type of care for umbilical area/nabi	Times per day performed?					Who performed this?			
	0/No	1	2	3+	Don't know/ missing	Mo-ther	Sha-shuri	Mthr's mother	Don't know/ missing/ other
Sek deoa (heat massage)	0	3	3	4	30	4	1	5	30
Put oil	0	1	0	2	37	0	1	1	38
Put turmeric juice	0	0	0	0	40	0	0	0	40
Put ginger juice	0	0	0	0	40	0	0	0	40
Turmeric powder	0	0	0	0	40	0	0	0	40
Wipe nabi with spirit	0	1	1	0	38	0	1	0	39
Wipe to clean	0	2	1	0	37	0	1	2	37
Apply medicine (Nebanol/ boric powder)	0	1	0	1	38	1	1	0	38
Put breast milk	0	0	0	0	40	0	0	0	40
Put chewed rice	0	0	0	0	40	0	0	0	40

Table 25. Reported day the umbilical stump fell off from the semi-structured interviews, N=40

Day reported that stump fell off	Frequency
DOB	0
1	3
2	1
3	6
4	9
5	9
6	4
7	5
8	0
9	1
Don't know	2

Table 26. Care of the umbilical area after the stump fell off from the semi-structured interviews, N=40

Type of care for umbilical area/nabi	Times per day performed?					Who performed this?			
	0	1	2	3+	Don't know/ missing	Mo-ther	Sha-shuri	Mthr's mother	Other
Sek deoa (heat massages)	0	8	18	4	10	0	14	2	14
Put oil	0	1	4	3	32	2	1	3	2
Put turmeric juice	0	0	0	0	40	0	0	0	0
Turmeric powder	0	0	0	0	40	0	0	0	0
Wipe to clean	0	4	0	1	35	1	1	2	1
Apply medicine (Nebanol/ boric powder)	0	4	1	3	32	5	2	1	0
Put breast milk	0	0	0	1	39	1	0	0	0
Put ash	0	0	1	1	38	1	0	1	0
Other	4	2	1	0	33	4	0	3	0

Table 27. Wrapping practices during the first week from the semi-structured interviews, N=40

Question	Response categories	Frequencies
Material used to wrap baby	Used lungi/sari	39
	Tolpani/Katha	38
	Nima/Gangy/Jama	30
	Towel	13
	Other	13
	Napkin/Nappy	4
	Blanket	2
Who wrapped baby? (More than one response allowed)	Baby's Mother	24
	Mother RDW	22
	Shashuri	6
	Bon/Sister	2
	Deor/Debor	2
	Father	1
	Meye	1
	Brother	1
	Khala/Aunt	1
	Ja/Jal	1
	Nonori	1
	Maid	1

Question	Response categories	Frequencies
How often was the baby's nappies/cloths/katha changed? (Times per day)	2 times per day<br>3<br>4<br>5<br>6<br>7<br>8<br>10<br>11<br>12<br>13<br>14<br>15<br>16<br>18<br>20<br>22<br>30	0<br>2<br>2<br>1<br>0<br>1<br>1<br>4<br>0<br>13<br>4<br>1<br>5<br>1<br>2<br>1<br>1<br>1
Who changed nappies/cloths/katha of baby? (More than one response permitted)	Baby's Mother<br>Mother of RDW<br>Shashuri<br>Khala/Aunt<br>Chhele<br>Meye<br>Ja/Jal<br>Other	34<br>22<br>5<br>2<br>1<br>1<br>1<br>1
Were all the wrappings changed, or only those that were soiled?	All wrappings were changed<br>Only soiled wrappings changed	27<br>13
Was the baby washed or just wiped after urination, after bowel movements?	Yes, washed<br>No, just wiped	4<br>36
Was baby strapped close to the mother?	Yes<br>No	39<br>1
For how many hours per day was baby strapped/kept close?	2 Hours<br>3<br>4<br>5<br>6<br>7<br>8<br>9<br>10<br>12<br>18<br>Other	1<br>3<br>2<br>3<br>7<br>4<br>4<br>0<br>6<br>8<br>1<br>1

7.3 Pre-lacteal feeds and breast-feeding

Table 28. Feeding of newborn in Mirzapur and Sylhet

	Mirzapur	Sylhet
Breastfeeding	Universal	Universal
Feed colostrum to baby	Usually	Sometimes
Prelacteal feeds	Common	Universal
Foods and fluids for newborn	No food. Fluids: misrir pani, honey, goat's milk, cow's milk or powdered milk	Banana, rice powder, mukh saline, misrir pani, honey,

Breastfeeding is universally practiced in Mirzapur. Despite this fact, there are a number of ways in which local feeding practices depart from the recommended norms. These include prelacteal feeds such as water with palm sugar (misrir pani), delay of initiation of breastfeeding, and continued mixed feeding after initiation of breastfeeding:

> "The chauni fed the baby misri mixed with warm water. She fed as long as the baby took it. It wasn't until the morning, after about two or three hours, that I gave the baby breast milk."

A positive point about feeding practices in Mirzapur is that women commonly report feeding colostrums to their babies, and views of colostrums are generally more favorable.

Table 29. Reported initial feeding of newborn from semi-structured interviews, N=40

Question	Response categories	Frequencies
Did you feed the baby colostrum (shaldudh), or did you wait for your regular milk to come in before starting to breastfeed?	Yes<br>No, waited for regular milk	39<br>1
On what day did you first breastfeed the baby?	Day of delivery<br>1st day after delivery<br>2nd day after delivery	31<br>5<br>4
If you fed baby on day of delivery, how long did you wait to feed baby?	Within 1st hour<br>After 1 hour<br>After 2 hours<br>After 7 hours<br>After 8 hours<br>After 12 hours<br>Did not feed on 1st day<br>No response/don't know	19<br>6<br>3<br>2<br>1<br>1<br>3<br>5

Question	Response categories	Frequencies
What drinks were given to the baby BEFORE it was breastfed? (more than one response allowed)	Misrir pani<br>Other drinks<br>Mukh saline<br>Water	20<br>17<br>4<br>2
What foods were given to the baby BEFORE it was breastfed? (more than one response allowed)	Other<br>Pakna kola<br>Cow/Goat milk	24<br>8<br>4
Who gave these drinks/foods to the newborn baby BEFORE it was breastfed? (more than one response allowed)	Mother's mother<br>Baby's mother<br>Dhorni/Dhonni<br>Shashuri<br>Khala/Aunt<br>Other<br>Meye<br>Bhabi<br>Ja/Jal<br>Chhele	12<br>7<br>5<br>4<br>3<br>3<br>2<br>1<br>1<br>1
What drinks were given to the baby AFTER it was breastfed for the first time? (more than one response allowed)	Misrir Pani/Chinir Pani<br>Other drinks<br>Mukh Saline	19<br>19<br>3
What foods were given to the baby AFTER it was breastfed for the first time? (more than one response allowed)	Cow/Goat milk<br>Pakna Kola<br>Tin Dud<br>Honey<br>Other	9<br>6<br>3<br>1<br>19
Who gave these drinks or foods to the newborn baby AFTER it was breastfed for the first time?	Baby's mother<br>Mother's mother<br>Shashuri<br>Other<br>Meye<br>Baby's father<br>Chhele<br>Bhabi<br>Khala/Aunt<br>Ja/Jal	14<br>13<br>4<br>4<br>3<br>1<br>1<br>1<br>1<br>1
Did you feel you had enough milk for your baby during the first days?	Yes<br>No	14 (35%)<br>26 (65%)

Question	Response categories	Frequencies	
When after birth did you first feel that you had enough milk for the baby?	day of delivery	5 (12.5%)	
	1st day after	14 (35.0%)	
	2nd day after	11 (27.5%)	
	3rd day after	4 (10.0%)	
	4th day after	2 (5.0%)	
	9th day	1 (2.5%)	
	12th day	1 (2.5%)	
	No response	2 (5.0%)	
What foods can you take to increase the quantity of breastmilk? What foods did you take to increase the quantity of breastmilk?		Can take	Did take
	Moka Machh	25	14
	Dry Fish	3	0
	Choto Machh	18	10
	Fish	16	9
	Meat	12	8
	Kaligira	34	31
	Ada/Golmorich/Kalig	29	26
	Lau/Gourd	20	17
	Potatoes	21	19
	Green Banana	4	3
	Gorom Pani	13	9
	Other Foods	30	-
	Other Actions	-	3
What problems did you have with breastfeeding? For which problems did you take some action?		Mentioned	Action taken?
	Swollen breasts	9	4
	Painful breasts	19	7
	Baby suck only1 breast	4	1
	Pimple on breast	2	2
	Not enough milk	8	6
	Excessive milk	16	9
	Other	3	1

8 The sick newborn

8.1 Illnesses of newborns and careseeking

There are a number of differences in patterns of careseeking between Sylhet and Mirzapur, as summarized in the next table. In general families in Mirzapur are more likely than families in Sylhet to seek care from allopathic providers and hospitals, but continue to consult some types of traditional healers such as kobiraj. In Sylhet newborns are treated first at home with traditional treatments such as bangla Masala, tel pora, pani pora, amulets, boron, and haon. If the baby is not cured, then they seek care outside the home from a homeopathic provider, and only if that fails do they consult an allopathic provider or go to a hospital. In Mirzapur home remedies were stated to be the preferred option only for the illness mashipishi. In most other cases informants stated that they prefer allopathic treatment.

Table 30. Careseeking practices in Sylhet and Mirzapur

	Sylhet	Mirzapur
ANC	Low use of ANC	High use of ANC but want to deliver in home
Sources of care for sick newborns	Prefer homeopath for newborns, also go to kobiraj, mia-sab, imam, jhar-fuk	Prefer allopathic doctor and hospital, like Kumudini Hospital. Also consult kobiraj who treats with telpora but not panipora or gurpora
Transport	Road communication is good in Sylhet though the rate of hospital delivery is low.	Road communication is bad compared to Sylhet though the rate of hospital delivery is much greater than in Sylhet.
Perception of hospital	Avoidance of hospital care, very rare for people to go to the hospital.	At Mirzapur, people have good knowledge about hospital and go there whenever they need it.

In the first round of interviews, informants (N=19) mentioned the following ways in which they can tell if a newborn is sick: Baby doesn't want to eat (16/19), high temperature (13), baby cries all the time (13), diarrhea/loose bowel movements (9), cold/shordi (6), baby weak and quiet (4), cough/kashi (3), difficulty breathing (3) and blisters on body and face (3). Pneumonia (informants use the English term even when speaking in Bangla) and other respiratory illnesses are mentioned more than any other class of illnesses. In unprompted narratives in the first round the following illnesses were mentioned: Pneumonia (18/19), diarrhea (14), high temperature (8), bhapi uta/ham/measles (7), cold/thanda/shordi (6), jaundice/kawla (6), upri/batash laga caused by malevolent spirits (4) and dudh phera (small blisters on skin) (2). In structured probing in the second round, the list of illnesses shown in the table on the following two pages was elicited. Many illnesses affecting newborns are thought to be causes due to either exposure to excessive cold, or exposure to malevolent spirits. Many preventive practices therefore aim to protect newborns from one or the other of these two causes.

Table 31. Illnesses affecting newborns according to informants in Upazila Mirzapur

Illness terms	Approxi-mate English translation	Fre-quency of men-tion	Number of times men-tioned first	Symptoms	Cause and preventive measures	Treatments
1. Jor	Fever	10	3	Body feels warm	Cold	Various
2. Nimonee	Pneumonia	5	1	Chest in-drawing; difficulty in breathing	Cold; mother's cold	Injections
3 Dudh Rokkha.	Skin problems, blisters	4	1	Initially small blisters; redness of body; later pus in the blisters	Doesn't know	Village dr.; ointment
4. Dudh phera	Skin problems, blisters	2	1	Initially small blisters; redness of body; later pus in the blisters	Doesn't know	Wrapping the baby with aunt's blanket
5. Dudh gota	Skin problems, blisters	1	0	Initially small blisters; redness of body; later pus in the blisters	Doesn't know	Village dr.; Wrapping the baby with aunt's blanket
6. Jaundice	Jaundice	7	3	Eyes, hands, and legs become yellow	If the mother had jaundice during her pregnancy	Sun bathing; Doctors
7. Thanda laga	Cold	7	3	Can't suckle on mother's breasts; runny nose;	Cold; mothers activities	Applying heat massage; keeping the chest covered; meds
8. Sordi	Runny nose	9	4	Can't suckle on mother's breasts; runny nose	Cold; mothers activities	Applying heat massage; keeping the chest covered; meds
9. Kashi	Cough	5	0	Cough	Cold; mothers activities	Applying heat massage; keeping the chest covered; meds
10. Shash koshto	Difficult breathing	3	0	Can't suckle on mother's breasts;	Cold	Have to keep the baby warm
11. Shash Tan	Wheezing; pneumonia	1	0	Can't suckle on mother's breasts;	Cold; mother's activities	Have to keep the baby warm
12. Jonmer por shash koshto	Difficult breathing right after birth	1	0	Can't suckle on mother's breasts	Cold; mother's activities	Have to keep the baby warm
13. Koph Atka	Congestion	1	0	Dry cough; ghor ghor noise in the chest	Cold; mother's activities	Have to keep the baby warm
14. Mukhe Phaka	Blisters on the lips (white)	4	0	White blisters on inside the mouth and on the lips	Doesn't know	Apply shohaga with honey after using a rag soaked in mustard oil; village dr.

Illness terms	Approxi-mate English translation	Fre-quency of men-tion	Number of times men-tioned first	Symptoms	Cause and preventive measures	Treatments
15. Mukhe Gha	Blisters on the lips (white)	1	0	White blisters on inside the mouth and on the lips	Doesn't know	Apply shohaga with honey after using a rag soaked in mustard oil; village dr.
16. Chokh Utha	Pink eye	1	0	Redness of the eye; sleep in the eyes; can't open the eyelids properly	If mother's milk falls in the baby's eyes	Heat message; Doctor
17. Chokh die pani pora	Watery eyes; pink eye	1	0	Watery eyes; redness;	Doesn't know	-
18. Mashi pishi	Erythema neonatorum (for lack of common term)	7	0	Redness of the body; tiny blisters after birth	It's normal to have it after birth	Village doctor.; ointment, etc.
19. Shorire Phoshka	Blisters on the body	1	0	-	-	-
20. Khosh Pachra	Skin pustules	1	0	severe itching; redness due to itching; watery wounds;	Un-cleanliness	Not sure
21. Ham	Measles	2	0	Redness of the body; fever; the baby cries a lot	-	Allopathic doctor
22. Ghamachee	Prickly heat	1	1	Redness and puffy blisters	From extreme heat (during summer)	Use talc powder
23. Upri	Malevolent spirits	2	0	Various	Not abiding by certain rules during and after pregnancy	Keeping iron and fire close-by and not going out during mid-day and after sunset
24. Bau Batash	Malevolent spirits	2	0	Various	Not abiding by certain rules during and after pregnancy	Keeping iron and fire close-by and not going out during mid-day and after sunset
25. Nabhitay gha	Umbilical infection	1	1	Infection; foul smelling discharge	Unhygienic activities	Doesn't know
26. Tongkar	Tetanus	1	1	Hands and legs get crooked	Injections help to prevent	Doctor
27. Pet kharap/ haga hoy	Diarrhea	5	0	Runny stool;	Mother's unhygienic activities	Oral saline
28. Bhapi utha	Used inter-changeably to mean either ham or mashipishi	1	0	Redness of the body; small blisters; fever	Doesn't know	Allopathic doctor

8.2 Sources of advice and care

The first sources of advice for how to care for a sick newborn mentioned spontaneously in the first round of formative research were elder females including the mother-in-law (shashuriI (10/19) and village doctors (8/19). Others such as homeopaths, health clinics, TBAs, neighbors, Mirzapur hospital and the jhar fuk were mentioned by only one informant. Informants stated that the village doctor is the one who is most available for advice, and elder females are the most experienced in these matters. The village doctors again figured prominently in the first sources of care for sick newborns mentioned in the first round of formative research: Village doctor (7/19), Mirzapur Kumudini hospital (5), elderly person (3), husband (1), kobiraj (1), health clinic (1), Jamurki Hospital (1) and homeopath (1). The ones who were thought to know the most about treatment of sick newborns, following a similar pattern, were the village doctor (12), Mirzapur Kumudini Hospital (9), elderly persons (4), trained TBAs (4), jhar fuk (3), kobiraj (3), other hospitals (3) and MBBS doctors (2).

At least some of the popularity of the generally unlicensed village doctors relates to the role of husbands in careseeking. Rather than letting women leave house to seek care, husbands often go to village doctor and describe the situation, then buy a drug and bring it home. In several cases husbands were found to purchase oxytocin from village doctors and bring it home. The mothers of the newborns do not typically seek care on their own, but rather seek permission from their husband (15/19), an elder male or female (8), her own parents (2), and elder brother (2), or the husband's brother's wife (jal) (1).

8.3 Results of careseeking scenarios

The second round of formative research included a module with scenarios describing danger signs in mothers or newborns as well as providing other details of the context in which the illness occurred. Respondents were asked to identify symptoms that were of concern to them, and to state how they would respond in such a situation. The three scenarios presented were: 1) a woman with symptoms of preeclampsia, 2) a woman with prolonged labor, and 3) a baby with signs of infected umbilical stump and sepsis. Only results from the third scenario will be presented here. Each scenario was about 15 lines long. The first half of the third scenario is given here by way of example:

> Halima has 2 boys and just had a baby girl, Moni, one week ago. The TBA attended her delivery, and advised her not to feed shaldudh (colostrum) to the baby, and to start giving him modhu (honey) and misrirpani (water with palm sugar). She didn't breastfeed the whole first day. On the second day, Halima started breast feeding Moni every couple of hours. When she was changing Moni's katha, she noticed redness around the umbilicus. On the third day, the baby was moving less than usual all day long and didn't even cry as usual and didn't want to suckle when given the breast. Halima also noticed yellow discharge around the baby's umbilicus. At around sunset, Halima carried her and tried again to breast feed her. She felt that the baby's hands and feet were cold. Her mother-in-law (shashuri) told her not to worry, the whole day was cold, and this is the reason why the baby feels cold now. She said the best treatment for the baby would be to massage its back

with warm mustard seed oil mixed with a little garlic. The shashuri also said the baby needs to sleep – and that is why she doesn't want to suck on her breast.

For all three scenarios recognition of danger signs was very high, even when they were hidden among other irrelevant information. In the newborn scenario the following danger signs were identified by respondents, unprompted, after they were read the scenario:

Redness around umbilicus	18/19	Doesn't cry as usual	14/19
Umbilical discharge	16/19	Moving less than usual	13/10
Doesn't want to breastfeed	15/19	Baby is cold	8/19

Coldness was the least recognized danger sign, and this finding is compatible with other findings in this formative research that suggest that while awareness of acute respiratory illnesses and their symptoms (coughs and colds) are high, awareness of the importance of a baby feeling cold to the touch is much lower.

Table 32. Preferred sources of care for three scenarios on careseeking from the semi-structured interviews, N=19

	Careseeking scenario		
	#1. Preeclampsia	#2. Prolonged labour	#3. Newborn sepsis
Kumudini Hospital	13	19	19
Village doctor	1	6	6
Shashtho apa	2	1	1
Union/Thana facility	2	1	1
Satellite clinic	4	0	0
TBA/chauni	3	0	0
Doctor	2	0	0
Grameen sh. Kendro (Rural health center)	1	0	0

For all three scenarios respondents stated an overwhelming preference for careseeking from Kumudini hospital. This is in disagreement with other information presented above that revealed a preference for village doctors. It may be that the more serious nature of the symptoms presented in the three scenarios led respondents to state they would seek care from the hospital. The interviewers for the different data collection procedures were the same. Village doctors were still mentioned by a significant number of respondents for the prolonged labor and newborn sepsis scenarios. For both types of situations other interviews showed that village doctors are often sought out for oxytocin injections (prolonged labor) or other medicines.

What the scenario method did not indicate, but which was found in other interviews, was that delays in seeking care are considerable. For example, although residents of Mirzapur commonly seek care from Kumudini Hospital in cases of prolonged labor, there is often a long delay in seeking care. Women are taken to Kumudini Hospital only after trying other closer

sources of care, especially drugs and injections from village doctors and compounders, as shown in the following quote:

> "My labor started right when I completed my tenth month. I didn't tell anyone, since there is a saying in the village that you shouldn't talk about it. However, I couldn't take it the next day, and my mother called the dai (TBA). I was in pain of and on – the water broke too. Dai went and got some homeopathic meds, but it didn't work. Then she asked me to go to the hospital, but I didn't want to go there. When nothing happened the next day and the labor continued, a (village) doctor came and gave an injection. That didn't work either. It was on the third night that I went to the hospital..." - Dilara/Nagar/Oct.28,2002

The scenarios also permitted direct comparison of the types of transport and the costs of transport that respondents estimated they would have to pay if they found themselves in a similar situation.

Table 33. Preferred mode of transport for three scenarios on careseeking from the semi-structured interviews, N=19

	Careseeking scenario		
	#1. Preeclampsia	#2. Prolonged labor	#3. Newborn sepsis
Van/trolley	11	13	15
Rickshaw	9	7	10
Tempo	6	6	8
Walk	3	1	4
Boat	2	1	3
Carry pt on board	2	2	2
Bus/car/baby taxi	1	1	1
Shallow boat	0	1	0

Respondents stated that their largest expenses related to careseeking are for hospital care Transport is an expense, but much less, as shown in the next table. Some families save money for emergencies, but most take loans from neighbors and relatives when the need arises.

> "In Kormokar Para, everyone comes forward in times of need. Right next to us is the Dhoni Bari and Thakur Ma Bari – they gave us some money. Everybody comes forward. Sometimes, people collect money through the school. Besides, one can always ask for a loan from a neighbour."-Afsana/Nardhana/Feb.1,2003

A smaller number of families obtain loans from village cooperative associations (Somobay somiti). They are associated with various NGOs: BRAC, Grameen, Udoy, Basha, SSS, Buro Tangail. In interviews with members of the associations, only 2 out of 16 stated that they had funds that could provide support for emergency care for sick newborn. In general they provide loans to members only, who need to have savings, obtain the signature of 3-5 other members and demonstrate that previous loans have been repaid before they can obtain a new loan. All of this suggests that they are unlikely to be a viable option for improving financial access to care for sick newborns in the current project.

Table 34. Estimated cost of transport for three scenarios on careseeking from the semi-structured interviews, N=19

Estimated cost in Bangladeshi taka ~57 taka = US $1	Careseeking scenario		
	#1. Preeclampsia	#2. Prolonged labour	#3. Newborn sepsis
Van/trolley <50 tk	3	3	3
51-100 taka	4	4	1
101-300 taka	5	6	2
Rickshaw <50 tk	4	1	3
51-100 taka	4	1	4
101-300 taka	3	1	2
Tempo <50 tk	0	1	1
51-100 taka	2	3	4
101-300 taka	5	5	6
Van/trolley <50 tk	3	3	3
51-100 taka	4	4	1
101-300 taka	5	6	2

8.4 Role of TBAs in referral of sick newborns

In the first round of formative research it was found that TBAs often play a key role in the referral of sick newborns, so further questions were asked about referral in the semi-structured interviews with TBAs. The TBAs stated that they do not like to handle complicated deliveries or prolonged labor, and this was confirmed in interviews with recently-delivered women:

> "I had called for two TBAs (chauni) – one of them was trained. She (trained TBA) said that there was no pain but you could see the head of the baby... it was not good. Then she asked us to take the mother to the hospital."Afsana/Satiachori/Oct.27.2002

In the semi-structured interviews 11 out of 19 TBAs stated that had previously referred a sick baby to a hospital or health provider, of whom 6 had done so one time only, 2 had done it twice, 2 has done it 3-4 times and one had done it more than 20 times. The symptoms of the babies who had been referred were yellow eyes/jaundice (3), difficulty breathing (2), and others mentioned once only: didn't take milk, cold, vomiting, became thin, bad air, low birth weight, white blisters on face and no menstrual canal.

The sickness of the baby eventually referred by the TBA sometimes was noticed during a home visit by the TBA (3/11 referred cases), but more often the family had called the TBA (8/11 referred cases). The decision to refer (according to the TBA) was made by the TBA only in 9 of the 11 cases of referral, and the TBA and family jointly in two cases. Household members who participated in the decision to send the baby for outside care were the paternal grandfather (3), the maternal grandmother (2), the baby's mother (3), the baby's father (3), the baby's uncle (1), the paternal aunt (1), the jal (1) and the paternal aunt (1).

In the 11 cases of referral the baby most often was taken to Kumudini hospital (9/11), but other cases went to the village doctor (1/11), Jamruki hospital (1) and the kobiraj (1). The baby was accompanied by its mother (11/11), the TBA (2), the mother-in-law (2), another senior female from the household (5), the husband (4) or others (3). Transport used was a bus/minibus/van (3), a rickshaw (7), walking (1) and a tempo (1)

8.5 Village doctors – General description

The formative research on the village doctors was conducted in order to partially assess the situation of primary health care provisions and partially to figure out how we could use the existing framework of health care provisions to improve and/or make the intervention plan for PROJAHNMO, Mirzapur more effective. We interviewed ten (10) village doctors from all five study zones in Mirzapur Upazila of Tangail district. Our data collection team was composed of Dilara Afroz, Afsana Akhter, and Nabeel Ashraf Ali. We believe, though our study sample has been small, the results are representative of the current practices of the village doctors in the study area.

Village doctors are the first-line health care providers in the context of rural Bangladesh – Mirzapur is no exception. Villagers usually refer to the village doctors as “gramer daktar.” However, many refer to them by their names i.e. “‘x’ daktar.” This shows how the villagers relate to these providers on a personal note. The VDs on the other hand also refer to their patients by their names and know much about their family situations and other related information.

Most of the providers/village doctors are young males. Six out of ten who were interviewed were below 40, with only one person over 60. Their youth enables them to pay household visits to their patients if they think it is necessary. Most, if not all, VDs are males (neither did we interview a female VD nor did we hear about one). This is another factor that helps them be as mobile as they are. However, home visits are not as common as one would like them to be.

Most of the village doctors gained the necessary experience/training as apprentices of a senior family member or relative who also practiced before them. Some simply sat and learned the “trade” at a pharmacy. A few received semi-formal trainings entitled “Rural Doctor/Village Doctor and Pharmacist Training,” while a few others received a diploma in homeopathic medicine. The prevalence of homeopaths in Mirzapur is relatively less than in Sylhet, though their utilization rate for the treatment of neonates is still significant.

The kind of training courses and diplomas mentioned by the VDs are in no way beyond doubt. Some of the certificates could very well be forged – their hesitancy to show the papers and answer certain questions regarding the training sessions is expressive of that fact. This was undoubtedly more confounded by our presence – people who they did not know what to think of.

The range of illnesses the VDs treat is wider than one would expect from someone with very little training. In fact, one VD mentioned, “We attempt to treat each and every person who come to us for treatment. We refer them elsewhere only when our first attempt fails.” In any case, most common diseases/illnesses they treat among the adults are fever, dysentery, common

cold, and gastric ulcers. Diarrhea, fever, common cold, pneumonia, skin diseases, and dysentery were most commonly mentioned regarding the children for whom they provide treatments.

8.6 Role of village doctors in maternal care

Half of the VDs interviewed mentioned that they regularly treat women who are having problems during labor and delivery. The range of complications that the VDs attempt to treat during labor and after the delivery suggests that women (more appropriately their husbands and/or other family members) seek out help from the village doctors for a variety of reasons. In addition, it testifies to the claim that the village doctors are the first line health care providers for the villagers – in this particular case, the rural pregnant or recently delivered women.

The VDs attempt to treat simple complaints like weakness during and after pregnancies to more complicated situations like irregular positioning of the baby in the womb. Commonest complaints are weakness, absence of labor around the time of delivery, pain (not labor) in the lower abdomen, bleeding or water-breaking, white discharge, and nausea. However, few VDs mentioned that they also treat women who have more complicated problems like prolonged labor, incomplete delivery, post delivery bleeding, and no movements of the baby in the womb.

The treatments VDs offer range from prescribing/selling antibiotics and injecting Oxytocin to simple advices and administration of vitamin tablets. A substantial proportion of VDs mentioned that they refer all the complicated cases (cases they deem are beyond them…) to the hospital. However, the truth of the matter should be weighed in the light of their responses to other questions, i.e. what treatments they do offer, and the fact that they try to treat every patient to the best of their abilities before they refer him/her to the hospital. This would also explain at some length their understanding of the term "complicated."

The issue of referral in the context of village doctors is tricky. On one hand they talk of numerous signs and/or problems for which they refer their patients, but on the other hand, they mention similar signs and/or problems for which they provide treatments as well. Generally speaking, the sequence of events in terms of referral around the time of delivery (during labor, immediately before and during delivery, and after delivery) follows the pattern below:

Home management ➔ Calling TBA (dai, chauni) ➔ TBA management ➔ Calling VD ➔ VD management ➔ Referral

The idea is, be it the TBA/*dai/chauni* or the VD, they first try to manage the situation on their own and refer only when they fail. With regard to the referral site, it goes without saying that Kumudini Hospital is the most trusted and used facility.

The case of Oxytocin usage could be instructive in this matter. All of the VDs said "yes," people do come and ask for injections during labor and delivery. They all know about the injection – most, call it OCIN – a brand name for generic Oxytocin. VDs are aware of the fact that they are not supposed to give these injections. This is part of the reason why they decline to admit its use. However, 5 out of 10 VDs admitted that they have and do (on occasion) prescribe

and administer Oxytocin (mostly due to the family's request). All of them said the same thing about what it does. Plainly put, they believe it increases the pain, and thereby quickens the birthing process. One VD mentioned that they only prescribe/administer upon the advice of the *dai*. Another mentioned that the delivery happens quickly if you give OCIN. Two VDs mentioned that the injection helps to open the mouth of the birth canal. Along with Oxytocin, two VDs mentioned Hysomide and No-Spa as helping agents in labor and delivery.

In most cases, VDs administer the injections themselves, but they also mentioned the *dais* also give the injections, suggesting a more widespread usage of the drug.

All the VDs mentioned that it was the pregnant or recently delivered women's husband who came in to get the treatment for their wives. In some occasions, the mother or some other senior female relative of the husband would accompany them. This is expressive of the husband's role in maternal care, especially when it comes to seeking care from outside. In most of the cases, husbands have the final word in deciding whether treatment would be sought from outside. This is true even when the VD fails and refers the patient to the hospital. Furthermore, majority of the husbands accompany their wives to the hospital. Therefore, it is evident that the role of husbands is vital from buying drugs and seeking advices from the VD to transporting the patients to the hospital.

8.7 Role of village doctors in newborn care

VDs treat most of the illnesses that they believe afflict the neonates. If we place diarrhea, fever, pneumonia, and common cold on one end of the spectrum, then jaundice, bloated stomach, blisters and pimples, and lack of appetite would be on the other end of the spectrum.

The range of treatments the VDs provide for the neonates are as wide as the diseases they treat. They provide antibiotics as well as health advice. They give injections as well as ORS. However, their knowledge on etiologies of different diseases seems to be a mixture of biomedical and traditional knowledge. On one hand they talk about virus and bacteria, while on the other hand they relate mother's milk with just about every neonatal sickness.

Mothers are the primary caretakers who come over to the VDs to seek treatment for the babies. In most cases, they bring their babies with them. However, it should be mentioned here that fathers also accompany the mothers on their trip to the dispensaries where the VDs sit. It seems the husbands play an instrumental role in terms of seeking care for the neonates.

The pattern of referring the neonates follows a similar pattern (like that of the pattern reflected in maternal care). As in the case of most other diseases for people across the age groups and gender, the VDs attempt to treat the neonates themselves first before they refer. However, the situation is different when they find a neonate in dire condition (when they are contacted at a later stage of the sickness). During these situations, the VDs refer the neonates to hospitals right away.

In terms of specific sicknesses for which the VDs refer neonates to the hospital, pneumonia and severe diarrhea top the list. Sometimes when the VDs refer neonates to the hospital for cold, congestion, or something similar, it is because they are afraid that it could be pneumonia.

The decision-making process is almost always a combined effort at the household level. Though the VDs give their opinions, without the family's consent, these opinions are of no value. The typical sequence of events in the decision-making process regarding referrals follow the basic pattern outlined below:

Mother raises the concern ➔ Mother & mother-in-law discuss the matter ➔ Mother & MIL pursue the father ➔ They go see the VD if the father concedes ➔ VD management ➔ VD gives his opinion (in this case, referral) ➔ Mother discusses with husband and MIL ➔ Family visits the referral facility

The general feeling of the referred clientele regarding the referral facility and the care received there is positive.

8.8 Implications for intervention design

In contrast to Sylhet, families in Mirzapur have fairly good knowledge of danger signs during pregnancy, labor, delivery and the newborn period. They are more aware of pneumonia and diarrhea as problems of newborns, perhaps influenced by various nation-wide campaigns on child health. There is limited awareness of other major causes of mortality in newborns such as asphyxia and hypothermia. The main sources of care are Kumudini hospital and village doctors who are unlicensed and have little or no training. Husbands play more active role in careseeking than in Sylhet, and need to be taken into account in any strategy to promote different patterns of careseeking. Van/trolley, rickshaw and tempo are main forms of transport. There is limited access to funds for careseeking and treatment. The village associations (somobay somiti) probably have a limited, if any, role to play in facilitating financial access. Both TBAs and village doctors commonly refer both mothers and newborns, but there are significant delays in referral, and referral may only come after all other avenues have been exhausted including injections of oxytocin.

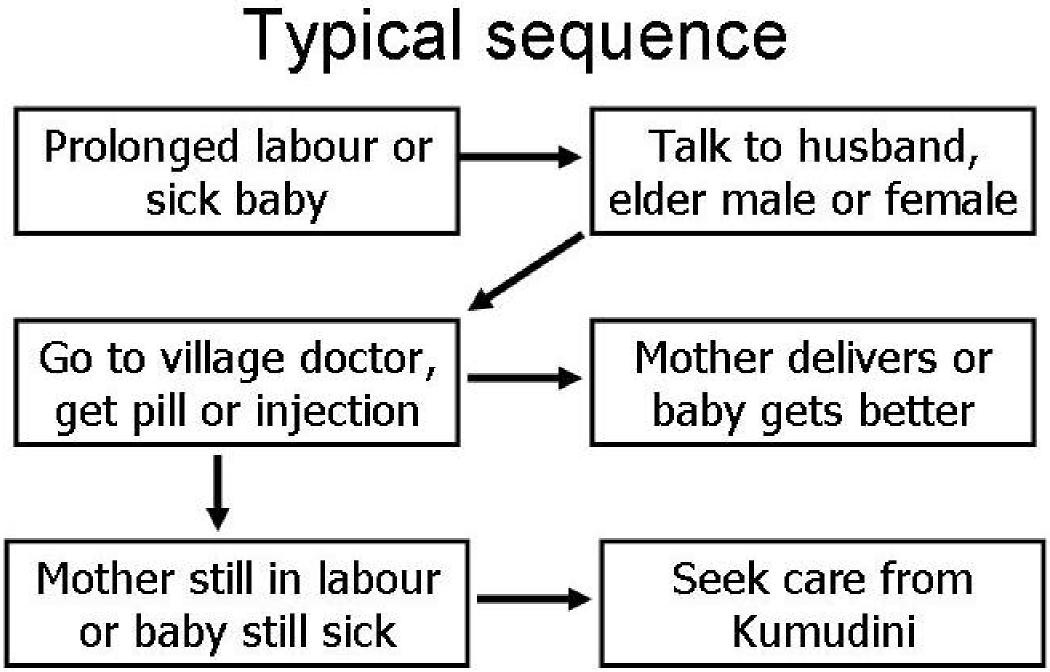

The above diagram summarizes a typical sequence of careseeking. While families commonly seek care from Kumudini Hospital, they only do so after seeking care from elder males or females and village doctors.

The influence of village doctors is pervasive. They need to be taken into account in the design of the intervention. There are some positive aspects of their current practice. They are young, which makes them mobile, and enables them to build a personal relationship with the villagers through home visits. Their youth can also have a negative influence. Young people are desperate to find a way to make money. Being a VD appears to some as a lucrative job. They are in the business not for a desire to heal the sick, but mostly because they thought it would be a good way to earn a little. The demand for their presence is noticeable within the villages. In one of the study zones we observed a market place with six pharmacies. One could call this pharmacists' market, since there were more pharmacies than anything else in that market.

They should definitely be included within the intervention design, for a) their incorrect practices are potentially harmful even lethal for both the baby and the mother, b) their correct practices would/could ensure safer deliveries and healthier newborns, and c) they could be instrumental in effective behavioral changes both at the community and household levels. Some of the specific reasons are as follows: 1) They are the first line health care providers, both for adults and neonates; 2) They provide Oxytocin without having an extensive knowledge of what the drug may or may not do; 3) They should be motivated to refer patients (and then they should in turn motivate the patient/patient's family) without taking the risks on them. Therefore, VDs should know when to refer; 4) If trained well, the VDs could change their own practices, and do

the "right thing" when needed; 5) Fathers have direct access to them… they would be a good way to disseminate our messages to the community, especially the men; 6) Besides the family of the parturient woman, TBAs and VDs are the only care providers with a little experience who would be around or could be reached quickly. We believe, with a little training, these cadres of providers could form a good neonatal team at the household level. They have the potential to provide messages on birth preparedness and newborn care especially to men when they visit the shops (dispensary/pharmacy), and to families when the VDs go out on calls. They could also paly an important role in the referral of mothers and newborns. TBAs could also play a key role in referral system. They need more education on danger signs.

9 References

Ahmed S, Sobhan F, Islam A & Barkat e K (2001) Neonatal morbidity and care-seeking behaviour in rural Bangladesh. *J Trop Pediatr* **47**, 98-105.

Baqui AH, Black RE, Arifeen SE, Hill K, Mitra SN & al Sabir A (1998) Causes of childhood deaths in Bangladesh: results of a nationwide verbal autopsy study. *Bull World Health Organ* **76**, 161-171.

Baqui AH, Sabir AA, Begum N, Arifeen SE, Mitra SN & Black RE (2001) Causes of childhood deaths in Bangladesh: an update. *Acta Paediatr* **90**, 682-690.

Barnes-Josiah D, Myntti C & Augustin A (1998) The "three delays" as a framework for examining maternal mortality in Haiti. *Soc Sci Med* **46**, 981-993.

Black RE, Morris SS & Bryce J (2003) Where and why are 10 million children dying every year? *Lancet* **361**, 2226-2234.

Blanchet T (1984) *Women, Pollution and Marginality: Meanings and Rituals of Birth in Rural Bangladesh*. Dhaka: The University Press Limited.

Chawla J (1994) *Child-Bearing and Culture. Women-Centered Revisioning of the Traditional Midwife: The Dai as a Ritual Practitioner*. New Delhi: Indian Social Institute.

Cosminsky S (1994) Childbirth and change: A Guatemalan study. In *Ethnography of Fertility and Birth*, pp. 195-219 [CP MacCormack, editor]. Prospect Heights, IL: Waveland Press, Inc.

Darmstadt GL, Black RE & Santosham M (2000) Research priorities and postpartum care strategies for the prevention and optimal management of neonatal infections in less developed countries. *Pediatr Infect Dis J* **19**, 739-750.

Darmstadt GL, Lawn JE & Costello A (2003) Advancing the state of the world's newborns. *Bull World Health Organ* **81**, 224-225.

Davis-Floyd RE & Sargent CF (1997) Introduction - The anthropology of birth. In *Childbirth and Authoritative Knowledge: Cross-Cultural Perspectives*, pp. 1-51 [RE Davis-Floyd and CF Sargent, editors]. Berkeley and Los Angeles, CA: University of California Press.

de_Zoysa I, Bhandari N, Akhtari N & Bhan MK (1998) Careseeking for illness in young infants in an urban slum in India. *Soc Sci Med* **47**, 2101-2111.

Hotchkiss DR, Eckert E & Macintyre K (2000) The role of health services research in the Safe Motherhood Initiative. *Am J Public Health* **90**, 810-811.

Islam MS, Rahaman MM, Aziz KM, Munshi MH, Rahman M & Patwari Y (1982a) Birth care practice and neonatal tetanus in a rural area of Bangladesh. *J Trop Pediatr* **28**, 299-302.

Islam MS, Rahaman MM, Aziz KM, Rahman M, Munshi MH & Patwari Y (1982b) Infant mortality in rural Bangladesh: an analysis of causes during neonatal and postneonatal periods. *J Trop Pediatr* **28**, 294-298.

Jeffery PM, Jeffery R & Lyon A (1989) *Labour Pains and Labour Power: Women and Childbearing in India*. London: Zed Press.

Jordan B (1987) The hut and the hospital: Information, power and symbolism in the artifacts of birth. *Birth* **14**, 36-40.

Jordan B (1993) *Birth in Four Cultures,* 4th edition ed. Prospect Heights, IL: Waveland Press, Inc.

Kalter HD, Salgado R, Moulton LH, Nieto P, Contreras A, Egas ML & Black RE (2003) Factors constraining adherence to referral advice for severely ill children managed by the

Integrated Management of Childhood Illness approach in Imbabura Province, Ecuador. *Acta Paediatr* **92**, 103-110.

Kalter HD, Schillinger JA, Hossain M, Burnham G, Saha S, de Wit V, Khan NZ, Schwartz B & Black RE (1997) Identifying sick children requiring referral to hospital in Bangladesh. *Bull World Health Organ* **75**, 65-75.

Kaosar A & Rashid SF (2000) *Discoursing Birthing Care*. Dhaka: The University Press Limited.

Lehmann D, Michael A, Omena M, Clegg A, Lupiwa T, Sanders RC, Marjen B, Wai'in P, Rongap A, Saleu G, Namuigi P, Kakazo M, Lupiwa S, Lewis DJ & Alpers MP (1999) Bacterial and viral etiology of severe infection in children less than three months old in the highlands of Papua New Guinea. *Pediatr Infect Dis J* **18**, S42-49.

Macintyre K & Hotchkiss DR (1999) Referral revisited: community financing schemes and emergency transport in rural Africa. *Soc Sci Med* **49**, 1473-1487.

Marsh DR, Darmstadt GL, Moore J, Daly P, Oot D & Tinker A (2002) Advancing newborn health and survival in developing countries: a conceptual framework. *J Perinatol* **22**, 572-576.

McGilvray DB (1994) Sexual power and fertility in Sri Lanka: Batticaloa Moors and Tamils. In *Ethnography of Fertility and Birth*, pp. 15-63 [CP MacCormack, editor]. Prospect Heights, IL: Waveland Press, Inc.

Mitra SN, Ahmed AS, Cross AR & Jamil K (1998) Bangladesh demographic and health survey, 1996-1997. Dhaka and Calverton MD: National Institute of Population and Training (NIPORT), Mitra and Associates, and ORC Macro.

Moss W, Darmstadt GL, Marsh DR, Black RE & Santosham M (2002) Research priorities for the reduction of perinatal and neonatal morbidity and mortality in developing country communities. *J Perinatol* **22**, 484-495.

Nordberg E, Holmberg S & Kiugu S (1996) Exploring the interface between first and second level of care: referrals in rural Africa. *Trop Med Int Health* **1**, 107-111.

Sutrisna B, Reingold A, Kresno S, Harrison G & Utomo B (1993) Care-seeking for fatal illnesses in young children in Indramayu, west Java, Indonesia. *Lancet* **342**, 787-789.

WHO (1999a) Bacterial etiology of serious infections in young infants in developing countries: results of a multicenter study. The WHO Young Infants Study Group. *Pediatr Infect Dis J* **18**, S17-22.

WHO (1999b) Serious infections in young infants in developing countries: rationale for a multicenter study. The WHO Young Infants Study Group. *Pediatr Infect Dis J* **18**, S4-7.

Winch PJ, Alam MA & PROJAHNMO (Manuscript in preparation) The social and spatial organization of the first forty days of life in Sylhet District, Bangladesh. *Journal of Biosocial Science*.
